# Mlh2 Is an Accessory Factor for DNA Mismatch Repair in *Saccharomyces cerevisiae*

**DOI:** 10.1371/journal.pgen.1004327

**Published:** 2014-05-08

**Authors:** Christopher S. Campbell, Hans Hombauer, Anjana Srivatsan, Nikki Bowen, Kerstin Gries, Arshad Desai, Christopher D. Putnam, Richard D. Kolodner

**Affiliations:** 1 Ludwig Institute for Cancer Research, University of California School of Medicine, San Diego, La Jolla, California, United States of America; 2 German Cancer Research Center (DKFZ), Im Neuenheimer Feld 581, Heidelberg, Germany; 3 Department of Cellular and Molecular Medicine, University of California School of Medicine, San Diego, La Jolla, California, United States of America; 4 Moores-UCSD Cancer Center, University of California School of Medicine, San Diego, La Jolla, California, United States of America; 5 Department of Medicine, University of California School of Medicine, San Diego, La Jolla, California, United States of America; Duke University, United States of America

## Abstract

In *Saccharomyces cerevisiae*, the essential mismatch repair (MMR) endonuclease Mlh1-Pms1 forms foci promoted by Msh2-Msh6 or Msh2-Msh3 in response to mispaired bases. Here we analyzed the Mlh1-Mlh2 complex, whose role in MMR has been unclear. Mlh1-Mlh2 formed foci that often colocalized with and had a longer lifetime than Mlh1-Pms1 foci. Mlh1-Mlh2 foci were similar to Mlh1-Pms1 foci: they required mispair recognition by Msh2-Msh6, increased in response to increased mispairs or downstream defects in MMR, and formed after induction of DNA damage by phleomycin but not double-stranded breaks by I-*Sce*I. Mlh1-Mlh2 could be recruited to mispair-containing DNA *in vitro* by either Msh2-Msh6 or Msh2-Msh3. Deletion of *MLH2* caused a synergistic increase in mutation rate in combination with deletion of *MSH6* or reduced expression of Pms1. Phylogenetic analysis demonstrated that the *S. cerevisiae* Mlh2 protein and the mammalian PMS1 protein are homologs. These results support a hypothesis that Mlh1-Mlh2 is a non-essential accessory factor that acts to enhance the activity of Mlh1-Pms1.

## Author Summary

Lynch syndrome (hereditary nonpolyposis colorectal cancer or HNPCC) is a common cancer predisposition syndrome. In this syndrome, predisposition to cancer results from increased accumulation of mutations due to defective mismatch repair (MMR) caused by a mutation in one of the human mismatch repair genes *MLH1, MSH2, MSH6* or *PMS2*. In addition to these genes, various DNA replication factors and the excision factor *EXO1* function in the repair of damaged DNA by the MMR pathway. In *Saccharomyces cerevisiae*, the *MLH2* gene encodes a MutL homolog protein whose role in DNA mismatch repair has been unclear. Here, we used phylogenetic analysis to demonstrate that the *S. cerevisiae* Mlh2 protein and the mammalian Pms1 protein are homologs. A combination of genetics, biochemistry and imaging studies were used to demonstrate that the Mlh1-Mlh2 complex is recruited to mispair-containing DNA by the Msh2-Msh6 and Msh2-Msh3 mispair recognition complexes where it forms foci that colocalize with Mlh1-Pms1 foci (note that scPms1 is the homolog of hPms2) and augments the function of the Mlh1-Pms1 complex. Thus, this work establishes the Mlh1-Mlh2 complex as a non-essential accessory factor that functions in MMR.

## Introduction

DNA mismatch repair (MMR) recognizes single base and insertion/deletion mispairs generated by errors in DNA replication and some forms of chemically damaged bases [1]–[5]. Both types of errors can lead to mutations if uncorrected. Arguably, the best understood MMR system is the *Escherichia coli* methyl-directed MMR system where mispair excision is targeted to the transiently unmethylated newly synthesized DNA strand before Dam methylase acts on the newly synthesized strand. MMR is initiated by the MutS homodimer, which directly recognizes mispaired bases in DNA. After mispair recognition, MutS recruits the MutL homodimer, which promotes the MutH-mediated cleavage of the unmethylated strand at hemi-methylated GATC sites. The MutH-generated strand discontinuity (nick) functions as the initiation site for an excision reaction that results in the degradation of a stretch of the newly synthesized strand followed by its resynthesis. However, there are other bacteria that lack MutH and do not use DNA methylation for strand discrimination [6]–[8]. In eukaryotes, the early steps of MMR are conserved with those in *E. coli* [1], [3]–[5] with the partially redundant MutS-related complexes, the Msh2-Msh6 and Msh2-Msh3 heterodimers, recognizing mispairs followed by recruitment of MutL-related complexes. Genetic evidence in the yeast *Saccharomyces cerevisiae* indicates that the Mlh1-Pms1 heterodimer (called MLH1-PMS2 in humans) is the primary MutL homolog complex that functions in promoting post-replication MMR [5]. In contrast to *E. coli*, the steps during MMR following the recruitment of the MutL homologs have remained poorly understood in eukaryotes and other organisms that lack methyl-directed MMR. Recent experiments in *S. cerevisiae* have indicated that MMR proteins are coupled to DNA replication and have demonstrated the existence of a short window of time after DNA is replicated during which MMR has to initiate [2]. These and other results suggest that some aspect of the DNA replication intermediates themselves may play a role in mediating strand discrimination in organisms that lack methyl-directed MMR [2], [9].

Most eukaryotes encode multiple MutL homologs that function as heterodimers. Mlh1-Pms1 (called MLH1-PMS2 in humans) possesses an endonuclease activity that can introduce single-stranded nicks into double-stranded DNA, suggesting that Mlh1-Pms1 functions as the equivalent of a combination of both the bacterial MutL and MutH proteins [10]–[12]. This endonuclease activity is required for MMR *in vivo* as well as for suppression of homeologous recombination and responses to DNA methylating agents [10], [13]–[15]. This endonuclease activity is also present in MutL homologs from bacteria lacking methyl-directed MMR [16]–[20].

*S. cerevisiae* encodes two additional MutL complexes, Mlh1-Mlh3 and Mlh1-Mlh2. Mlh1-Mlh3 plays only a minor role during MMR [21]–[23] but plays a major role in the resolution of recombination intermediates during meiosis [24]–[26]. Mlh3 contains the conserved metal-binding motif that is required for the endonuclease activity of Pms1, and mutations affecting this motif in *MLH3* cause defects in *MLH3*-dependent MMR and meiotic crossing over [27]; consistent with this, the Mlh1-Mlh3 complex has recently been directly shown to have endonuclease activity [28], [29]. Mlh1-Mlh2 is more poorly understood than either Mlh1-Pms1 or Mlh1-Mlh3 [22], [25], [30]–[32]. Mlh2 lacks the metal binding motif present in Pms1 and Mlh3, and in most studies reported, deletion of *MLH2* causes a weak or no mutator phenotype, and the results of double mutant analysis have been taken to suggest a partial redundancy between *MLH2* and *MLH3* in *MSH3*-dependent MMR [22]. It has also been reported that deletion of *MLH2* increases the frequency of reversion of the *lys2ΔA746* frameshift mutation reporter due to the formation of large deletions [22]. Deletion of *MLH2* does not affect meiotic crossing over or meiotic MMR, unlike deletion of *MLH3* or *PMS1* [25], [31]. The *mlh2Δ* mutation does increase the frequency of gene conversion events, suggesting a partial role for Mlh2 in preventing heteroduplex formation, but not in subsequent mismatch correction; this property is unique among the *S. cerevisiae* MMR genes [25], [31]. Consistent with the idea that Mlh2 plays a role in recombination, simultaneous deletion of *PMS1*, *MLH2* and *MLH3* was required to cause defects in a mitotic heteroduplex rejection assay equivalent to that caused by an *mlh1Δ* mutation [32]. An *mlh2Δ* mutation as well as deletions of *MSH2, MSH3, MSH6* and *MLH1* but not *PMS1* have also been reported to cause a modest increase in resistance to some DNA damaging agents like cisplatin, reminiscent of that seen in human and mouse cells [30]. However, it is unclear how *MLH2* contributes to either recombination or MMR and why loss of Mlh2 only results in weak phenotypes.

Here, we employ live cell imaging in *S. cerevisiae*, complemented by genetic and biochemical assays, to analyze Mlh2 function in MMR. A similar approach applied to Pms1 previously revealed that the accumulation of Pms1 foci can be used to distinguish between genetic defects that affect events prior to Mlh1-Pms1 loading and those affecting downstream steps [33]. We found that Mlh2 formed nuclear foci similar to Pms1, whereas Mlh3 did not. Mlh2 foci partially colocalized with Pms1 foci and were dependent on *MSH2*, *MSH6*, and *MLH1* but not *MSH3*. Mlh2 foci increased in abundance in strains with increased mispair formation and in strains containing mutations that disrupt downstream steps in MMR similarly to what was previously observed for Pms1 foci [33]. *In vitro*, Mlh1-Mlh2 was recruited to mispair-containing DNA by both Msh2-Msh6 and Msh2-Msh3. Deletion of *MLH2* caused a synergistic increase in mutation rates when combined with a deletion of *MSH6* or a promoter substitution that reduced the expression of Pms1; by contrast, no synergy was observed when deletion of *MLH2* was combined with deletions of *MSH3* or *EXO1*. Together, these data support the hypothesis that *MLH2* encodes a homolog of MutL that functions in conjunction with Mlh1 as a MMR accessory factor whose roles become increasingly important under conditions when other MMR components are limiting.

## Results

### Pms1 and Mlh2, but not Mlh3, form nuclear foci

**Figure 1:**
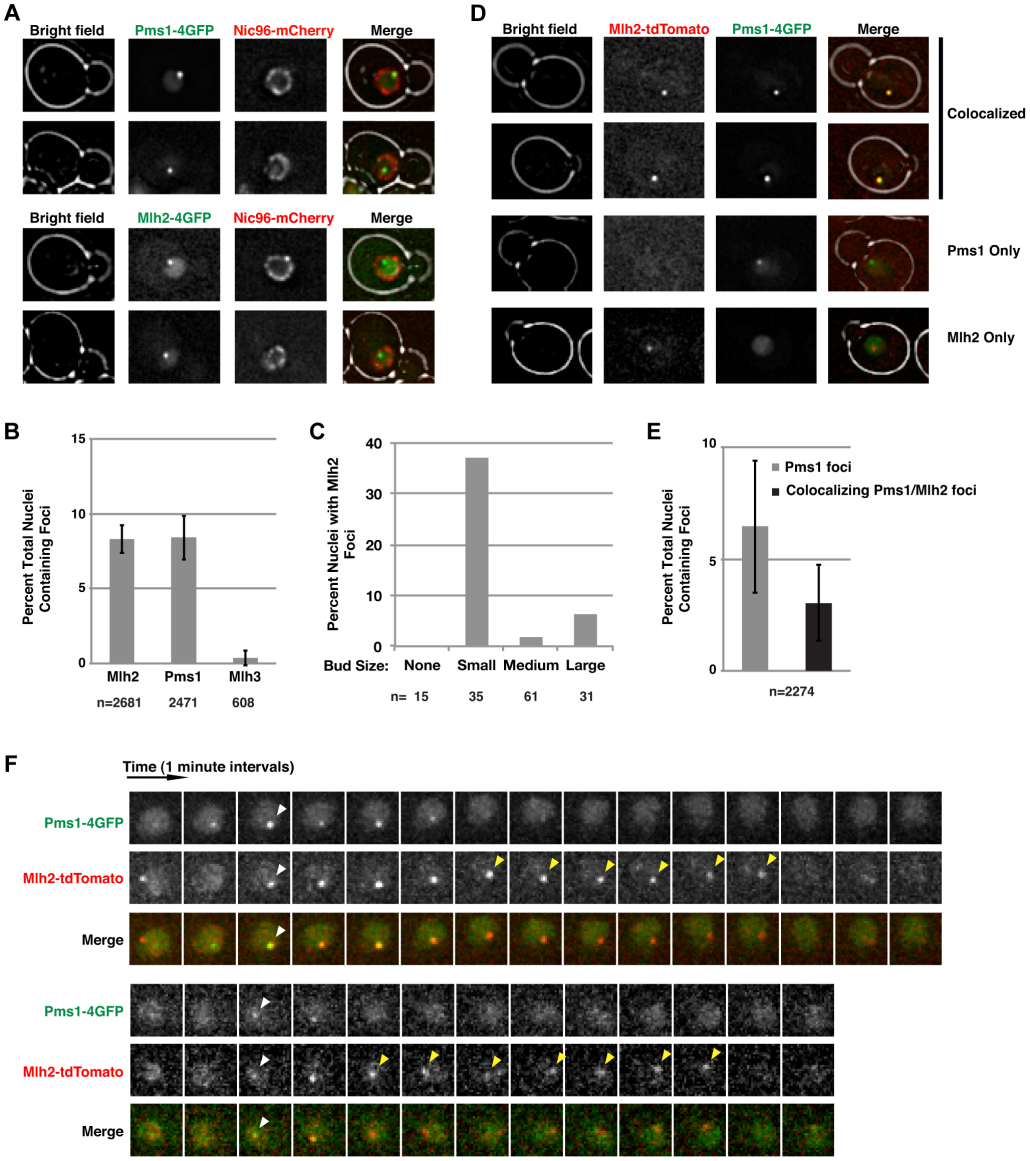
Mlh2 forms foci that partially colocalize with Pms1. (**A**) Images of cells expressing Pms1-4×GFP or Mlh2-4×GFP and Nic96-mCherry as a marker of the nuclear pore complex reveal the presence of nuclear Pms1 and Mlh2 foci. (**B**) Quantitation of Mlh2-4×GFP, Pms1-4×GFP, and Mlh3-4×GFP foci. Error bars indicate the standard error of the mean (SEM), and “n” indicates the number of cells examined. (**C**) Distribution of Mlh2-4×GFP foci according to bud size: no bud or small (<1.5 µm), medium (1.5–3 µm), or large (>3 µm) budded cells. “n” indicates the number of cells examined. (**D**) Images of cells expressing Mlh2-tdTomato and Pms1-4×GFP reveal foci that contain both proteins as well as foci containing only one of them. (**E**) Quantitation of Pms1 foci and Pms1 foci that colocalize with Mlh2. (**F**) Time-lapse images of cells with colocalized Mlh2-tdTomato and Pms1-4×GFP foci at one-minute intervals. White arrowheads indicate the start of colocalization and yellow arrowheads indicate Mlh2-tdTomato foci that persist after loss of the Pms1-4×GFP signal.

We recently visualized Pms1 in live *S. cerevisiae* cells and demonstrated that Mlh1-Pms1 foci are intermediates in MMR [33]. To gain insight into the roles of Mlh1-Mlh2 and Mlh1-Mlh3, we integrated a cassette encoding a 4×GFP tag at the 3′ end of the chromosomal *MLH2* or *MLH3* genes. Live cell imaging of these strains revealed that Mlh2-4×GFP formed nuclear foci similar to those observed for Pms1-4×GFP in ∼8% of unsynchronized cells (Figure 1A and B). Mlh2-4×GFP foci were almost exclusively observed in small budded cells, characteristic of cells in S-phase (Figure 1C). The observation that the Mlh2 foci visualized using different tags (4×GFP or a monomer tdTomato tag) were similar in number and appearance (Figure 1; see below) indicate that the fluorescent tags do not contribute to focus formation. In contrast to Mlh2-4×GFP, few if any cells had Mlh3-4×GFP foci (Figure 1B). The reason for the lack of Mlh3 foci is unclear, but this could indicate a limited role of Mlh3 in MMR or that the levels of Mlh3 at repair sites were too low to visualize.

To test if Mlh2 localizes to the same MMR intermediates as Pms1, we examined colocalization of Mlh2-tdTomato with Pms1-4×GFP by live cell imaging. Interestingly, the Pms1 and Mlh2 foci partially colocalized, with ∼50% of Pms1 foci showing colocalization with Mlh2 foci at the limit of resolution of deconvolution microscopy (Figure 1D and E). To further examine the relationship between the Pms1 and Mlh2 foci, image stacks were taken at one-minute intervals to observe the localization of Pms1 and Mlh2 foci over time. We analyzed 50 cases where we could follow the formation, retention for at least two images, and disappearance of an Mlh2 or Pms1 focus. In agreement with the single images, approximately half (21/50, 42%) of the foci displayed colocalization between Pms1 and Mlh2 at some point during their lifetimes (Figure 1F), 36% (18/50) were Pms1 foci with no colocalization of Mlh2, and 22% (11/50) were Mlh2 foci with no colocalization of Pms1.

For the 21 cases of colocalization observed by time-lapse imaging, Pms1 and Mlh2 first appeared within the same frame for the majority of events (13/21), indicating that both proteins were recruited to the same site within the one minute temporal resolution of the imaging (Figure 1F, bottom). In 3/21 events, a Pms1 focus preceded the colocalized Mlh2 focus, and in the remaining 5/21 events, the Mlh2 focus preceded the colocalized Pms1 focus. Interestingly, Mlh2 foci frequently persisted after the colocalized Pms1 was no longer detectable (11/21 cases); the Pms1 foci were usually visible for 1 to 4 min, whereas the Mlh2 foci were visible for up to 7 min (average = 4 min) after the associated Pms1 focus was no longer detected (11/21 cases, Figure 1F). In the remaining 10 cases, the colocalized Pms1 and Mlh2 foci disappeared at the same time. Strikingly, Pms1 foci were never present after the disappearance of Mlh2, suggesting that Mlh2 frequently marks the site of Pms1 foci and likely the site of MMR after Pms1 has been removed.

### Mlh2 foci behave similarly to Pms1 foci that are MMR intermediates

**Figure 2:**
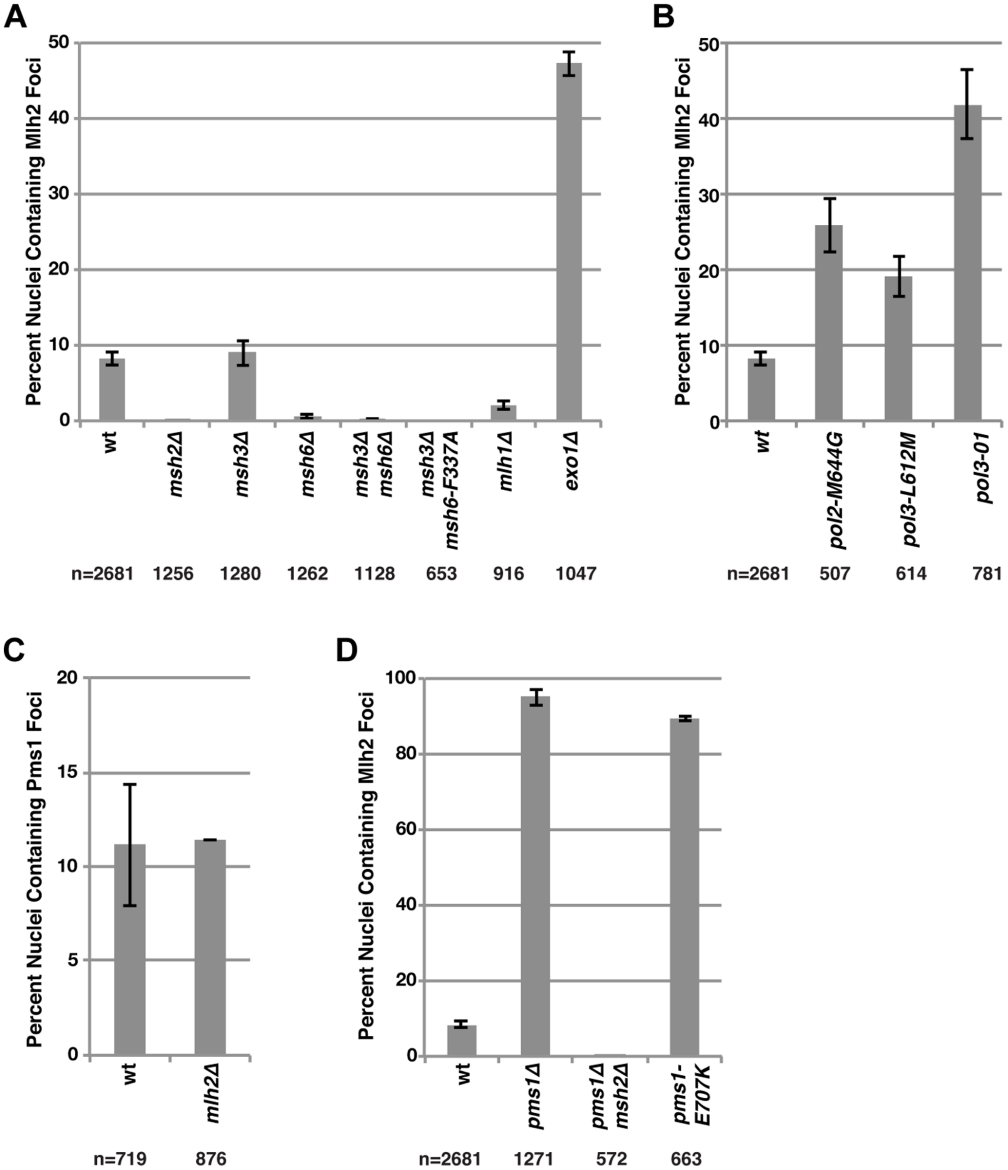
Mutations that perturb the abundance of Pms1 foci also perturb the abundance of Mlh2 foci. (**A**) The percentages of nuclei containing Mlh2 foci were quantified in strains containing mutations in MMR genes or (**B**) genes encoding the catalytic subunits of the DNA polymerases Pol2 and Pol3. (**C**) Quantification of the percentages of nuclei containing Pms1 foci in an *mlh2Δ* strain. (**D**) Quantification of the percentages of nuclei containing Mlh2 foci in strains containing the *pms1Δ* mutation, the *pms1Δ* mutation in combination with an *msh2Δ* mutation or the endonuclease active site *pms1* mutation *pms1-E707K*. Error bars indicate the SEM, and “n” indicates the numbers of cells examined.

In our previous study [33], we identified Pms1 foci as an MMR repair intermediate based on the following observations: (i) the formation of the Pms1 foci depended on mispair recognition by Msh2-Msh6 or Msh2-Msh3; (ii) the abundance of Pms1 foci increased with increasing levels of mispaired bases; and (iii) Pms1 foci increased in abundance in cells defective in MMR at steps that were downstream of recruitment of Mlh1-Pms1 [33]. Given the partial colocalization of Pms1 and Mlh2, we investigated Mlh2 foci by performing the same set of perturbations used to analyze Pms1 foci. Deletion of *MSH2*, which eliminates the Msh2-Msh3 and Msh2-Msh6 mispair recognition complexes, completely abolished Mlh2 foci (Figure 2A). Similarly, other mutations that disrupted mispair recognition also eliminated Mlh2 foci (Figure 2A), including the *msh3Δ msh6Δ* double mutation that eliminates both the Msh2-Msh3 and Msh2-Msh6 complexes and the *msh3Δ msh6-F337A* double mutation that eliminates Msh2-Msh3 and eliminates mispair binding by Msh2-Msh6 [34]. Deletion of *MSH6* alone also greatly reduced the number of Mlh2 foci, whereas deletion of *MSH3* had no effect, suggesting that the majority of Mlh2 foci were dependent upon Msh2-Msh6 but not Msh2-Msh3 (Figure 2A). The DNA polymerase epsilon and delta active site mutations (*pol2-M644G* and *pol3-L612M*, respectively) [35], [36] or a mutation causing a defect in the 3′-5′-exonuclease activity of DNA polymerase delta (*pol3-01*) [37], all of which increase the level of misincorporated bases, greatly increased the abundance of Mlh2 foci (Figure 2B). Deletion of *EXO1*, which encodes the 5′-3′ exonuclease that participates in the mispair excision reaction, increased the percentage of nuclei with Mlh2 foci to ∼50% (Figure 2A). Together, these results mirror what was previously observed for Pms1 foci, with the one notable exception that Pms1 foci were substantially increased and not decreased in an *msh6Δ* mutant suggesting that Pms1 and Mlh2 differ in their ability to be recruited by Msh2-Msh6 and Msh2-Msh3 *in vivo* [33].

We next examined the interdependencies of Pms1 and Mlh2 on their ability to form foci. Mlh2 has been shown to interact with Mlh1, but not with Pms1 or Mlh3, by yeast two-hybrid and affinity-capture mass spectrometry [25], [38]–[40], indicating the existence of an Mlh1-Mlh2 heterodimer that is distinct from Mlh1-Pms1 and Mlh1-Mlh3 heterodimers. Consistent with this, deletion of *MLH1* eliminated the vast majority of Mlh2 foci (Figure 2A). Deletion of *MLH2* had no effect on the number of Pms1 foci (Figure 2C). In contrast, deletion of *PMS1* drastically increased the percentage of cells containing Mlh2 foci to ∼95% (Figure 2D). This increase in Mlh2 foci could either be due to increased formation of the Mlh1-Mlh2 complex because of loss of competition for the Mlh1 partner protein by Pms1 or due to loss of Mlh1-Pms1 endonuclease activity and the consequent inhibition of downstream steps in MMR. To differentiate between these two possibilities, we measured the frequency of Mlh2 foci in cells containing the endonuclease active site *pms1-E707K* mutation that results in expression of an endonuclease-defective Mlh1-Pms1 complex. We observed a similar increase in the number of Mlh2 foci in *pms1Δ* and *pms1-E707K* mutant cells (Figure 2D), suggesting that the increase in Mlh2 foci in *pms1Δ* cells is likely due to the inhibition of downstream steps in MMR. The high levels of Mlh2 foci seen in the *pms1Δ* mutant were completely abolished by an *msh2Δ* mutation (Figure 2D), confirming that the increased recruitment of Mlh1-Mlh2 into foci in cells lacking Pms1 was dependent on Msh2.

### Mlh2 and Pms1 foci are increased by phleomycin treatment but are not present at a double-strand break generated by the I-*Sce*I endonuclease

**Figure 3:**
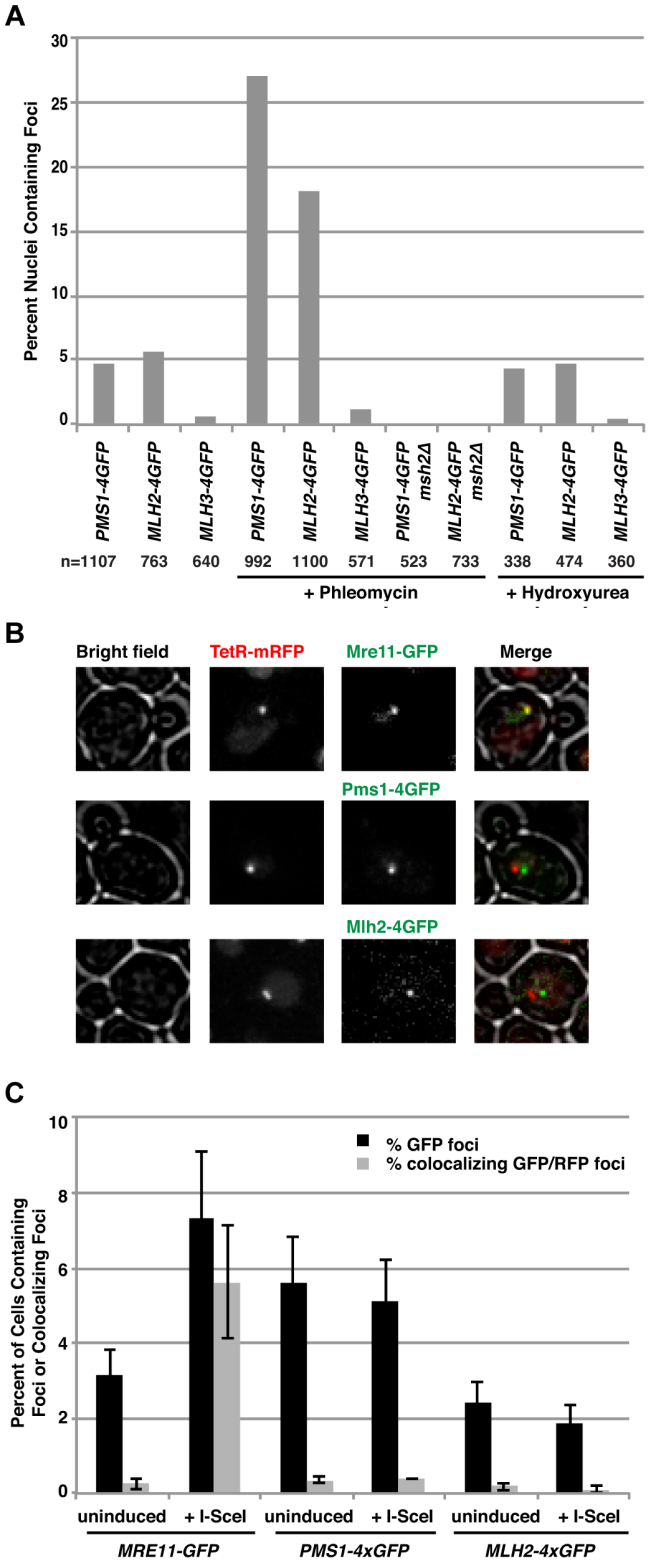
Pms1 and Mlh2 foci are induced upon treatment with phleomycin but not hydroxyurea and do not colocalize with DNA double-strand breaks. (**A**) Phleomycin, but not hydroxyurea, increases the number of Pms1-4×GFP and Mlh2-4×GFP foci in an *MSH2*-dependent manner. “n” indicates the numbers of live cells examined. (**B**) Images of cells expressing Mre11-GFP, Pms1-4×GFP, or Mlh2-4×GFP in a strain expressing TetR-mRFP and containing a tandem array of *tetO* sequences adjacent to an I-*Sce*I restriction site after induction of I-*Sce*I reveal that Mre11, but not Pms1 or Mlh2, colocalizes with the double-strand break. (**C**) Quantitation of cells containing Mre11, Pms1, and Mlh2 foci and their colocalization with the *tetO* array with and without I-*Sce*I induction.

In human cells, Mlh1 and other MMR components are recruited to sites where DNA damage has been induced by UV-laser micro-irradiation [41]–[43]. This has been interpreted as the recruitment of MutL homolog complexes to double-strand breaks (DSBs). Consistent with these observations, treatment of *S. cerevisiae* cells with the radiomimetic drug phleomycin greatly increased the percentage of cells containing Pms1 and Mlh2 foci (∼5-fold and ∼3-fold, respectively) (Figure 3A). This was unlikely to be the result of simply activating the DNA damage response (DDR) because treatment with hydroxyurea, which also activates the DDR by causing replication fork stalling by depleting dNTP pools, did not cause an increase in the abundance of Pms1 or Mlh2 foci (Figure 3A). As with foci formed in untreated cells, foci induced by phleomycin treatment were not observed in *msh2Δ* strains.

To determine if the Msh2-dependent Pms1 and Mlh2 foci were formed at DSBs and not other types of DNA lesions generated during phleomycin treatment, we investigated the recruitment of Pms1 and Mlh2 to a site-specific DSB generated by a galactose-inducible I-*Sce*I endonuclease. The DSB was generated adjacent to a tandem array of *tetO* sequences on chromosome V that was marked by expression of TetR-mRFP1 [44]. Cells expressing Pms1-4×GFP, Mlh2-4×GFP, or Mre11-GFP (as a positive control) were monitored before and after the addition of galactose to induce the DSB. Consistent with published results [45], Mre11 rapidly formed foci that colocalized with the *tetO* array (Figure 3B and C). In contrast, neither Pms1 nor Mlh2 formed foci that colocalized with the *tetO* array. These results suggest that the recruitment of Pms1 and Mlh2 to lesions induced by phleomycin (and the similar recruitment of mammalian MMR proteins to the sites of laser micro-irradiation) may not be due to recognition of DSBs but rather due to recognition of the other types of DNA damage induced by these treatments that might mimic mispaired bases.

### *MLH2* suppresses frameshift mutations when other MMR components are limiting

We next investigated the effects of deleting *MLH2* on MMR using the *hom3-10* and *lys2-10A* frameshift reversion and *CAN1* forward mutation rate assays (Table 1A). Deletion of *MLH2* alone did not cause a significant increase in mutation rate in either the frameshift reversion or forward mutation assays, in agreement with previous work [22]. We next tested if the *mlh2Δ* mutation exacerbated the defects caused by mutations in other MMR genes. The *mlh2Δ msh3Δ* or *mlh2Δ exo1Δ* double mutant strains did not exhibit mutation rates that were higher than the mutation rates of the single mutants. In contrast, the *mlh2Δ msh6Δ* double mutant strain exhibited a synergistic increase in the *hom3-10* and *lys2-10A* frameshift reversion assays but not in the *CAN1* forward mutation assay that, in addition to frameshift mutations, detects base substitution mutations and other kinds of mutations [46]. Although the frameshift reversion rates of the *mlh2Δ msh6Δ* double mutant were higher than that of the respective single mutants, the rates were still substantially lower than caused by the *msh3Δ msh6Δ* double mutation that eliminates mispair recognition and causes a complete MMR defect. The combination of the specificity of *MLH2* for suppressing frameshift mutations and synergy of the *mlh2Δ* mutation with the *msh6Δ* mutation but not with the *msh3Δ* mutation suggests that *MLH2* contributes preferentially to *MSH3*-dependent MMR.

**Table 1:** Mutation rate analysis of *mlh2Δ* in combination with mismatch repair mutations.

			Mutation rate (fold increase)^a^		
	Relevant genotype	RDKY	*Thr^+^*	*Lys^+^*	*Can^R^*
A	wild-type	5964	2.5 [1.8–3.8]×10^−9^ (1)	1.5 [1.0–5.0]×10^−8^ (1)	6.8 [3.9–8.7]×10^−8^ (1)
	*mlh2Δ*	7926	5.5 [3.0–8.4]×10^−9^ (2)	3.1 [1.3–4.0]×10^−8^ (2)	6.1 [4.2–11.7]×10^−8^ (1)
	*msh6Δ*	7965	9.9 [8.7–18.9]×10^−9^ (4)	4.1 [2.9–7.2]×10^−7^ (27)	5.1 [4.3–8.6]×10^−7^ (8)
	*msh3Δ*	6051	3.1 [2.0–4.2]×10^−8^ (12)	1.2 [1.0–3.1]×10^−7^ (8)	1.2 [0.7–4.5]×10^−7^ (2)
	*exo1Δ*	7884	1.2 [0.5–3.0]×10^−8^ (5)	1.0 [0.5–1.7]×10^−8^ (7)	7.7 [5.9–13]×10^−7^ (11)
	*mlh2Δ msh3Δ*	7923	9.0 [7.3–15.1]×10^−9^ (4)	8.3 [3.9–12.8]×10^−8^ (6)	7.5 [3.9–9.6]×10^−8^ (1)
	*mlh2Δ msh6Δ*	7924	8.5 [3.5–12.4]×10^−8^ (34)	2.7 [1.2–3.9]×10^−6^ (178)	7.0 [2.7–12.0]×10^−7^ (10)
	*mlh2Δ exo1Δ*	7925	6.7 [4.0–16.1]×10^−9^ (3)	8.2 [3.4–22.2]×10^−8^ (5)	3.8 [2.0–4.7]×10^−7^ (6)
	*msh3Δ msh6Δ*	6098	5.1 [2.9–13]×10^−6^ (2040)	3.4 [2.0–5.0]×10^−5^ (2267)	2.0 [0.8–5.6]×10^−6^ (29)
B	*tetO_2_-PMS1* (YPD)	8160	3.0 [1.5–5.9]×10^−9^ (1)	3.2 [1.9–4.6]×10^−8^ (2)	4.4 [3.4–8.4]×10^−8^ (1)
	*tetO_2_-PMS1* (+Dox)	8160	4.0 [2.5–5.8]×10^−8^ (16)	1.3 [0.9–1.5]×10^−6^ (89)	1.0 [0.9–1.4]×10^−7^ (2)
	*mlh2Δ* (YPD)	8159	1.0 [0.7–2.4]×10^−8^ (4)	7.9 [3.8–10.1]×10^−8^ (5)	1.0 [0.3–1.5]×10^−7^ (2)
	*mlh2Δ* (+Dox)	8159	6.0 [5.3–14.1]×10^−9^ (2)	6.9 [3.8–11.3]×10^−8^ (5)	1.1 [0.7–1.6]×10^−7^ (2)
	*tetO_2_-PMS1* (YPD) *mlh2Δ*	8161	5.2 [4.0–8.4]×10^−9^ (2)	1.4 [0.7–1.7]×10^−7^ (9)	4.3 [2.7–5.6]×10^−8^ (1)
	*tetO_2_-PMS1* (+Dox) *mlh2Δ*	8161	1.2 [1.0–1.7]×10^−7^ (48)	3.1 [2.5–4.0]×10^−6^ (207)	1.7 [1.4–2.0]×10^−7^ (3)

a Median rates of *hom3-10* and *lys2-10A* reversion and inactivation of the *CAN1* gene with the 95% C.I. in square brackets and fold increase relative to the wild-type in parentheses.

*tetO_2_-PMS1, PMS1* driven by the *tetO_2_* promoter; Dox, doxycycline.

Given the results of the Mlh2 localization studies, we hypothesized that Mlh2 becomes more important for MMR under conditions where the major MutL-related complex Mlh1-Pms1 is limiting. To test this idea, we took advantage of the previously reported tetracycline repressible system [47] to regulate Pms1 expression (*tetO_2_* promoter) in a doxycycline-dependent manner. After titrating doxycycline, we found that 10 µg/ml of doxycycline resulted in partial downregulation of Pms1 protein expression (Figure S1) and a weak MMR defect in the frameshift reversion assays but not in the *CAN1* forward mutation assay (Table 1B). Consistent with the hypothesis, we observed a synergistic increase in the mutation rate in the frameshift reversion assays when the *mlh2Δ* mutation was combined with reduced expression of Pms1. Thus, Mlh2 becomes more important for MMR when the level of Pms1 is reduced, suggesting that Mlh2 normally plays an accessory role in MMR.

### Mlh1-Mlh2 is recruited to mispair-containing DNA by Msh2-Msh6 and by Msh2-Msh3 *in vitro*

**Figure 4:**
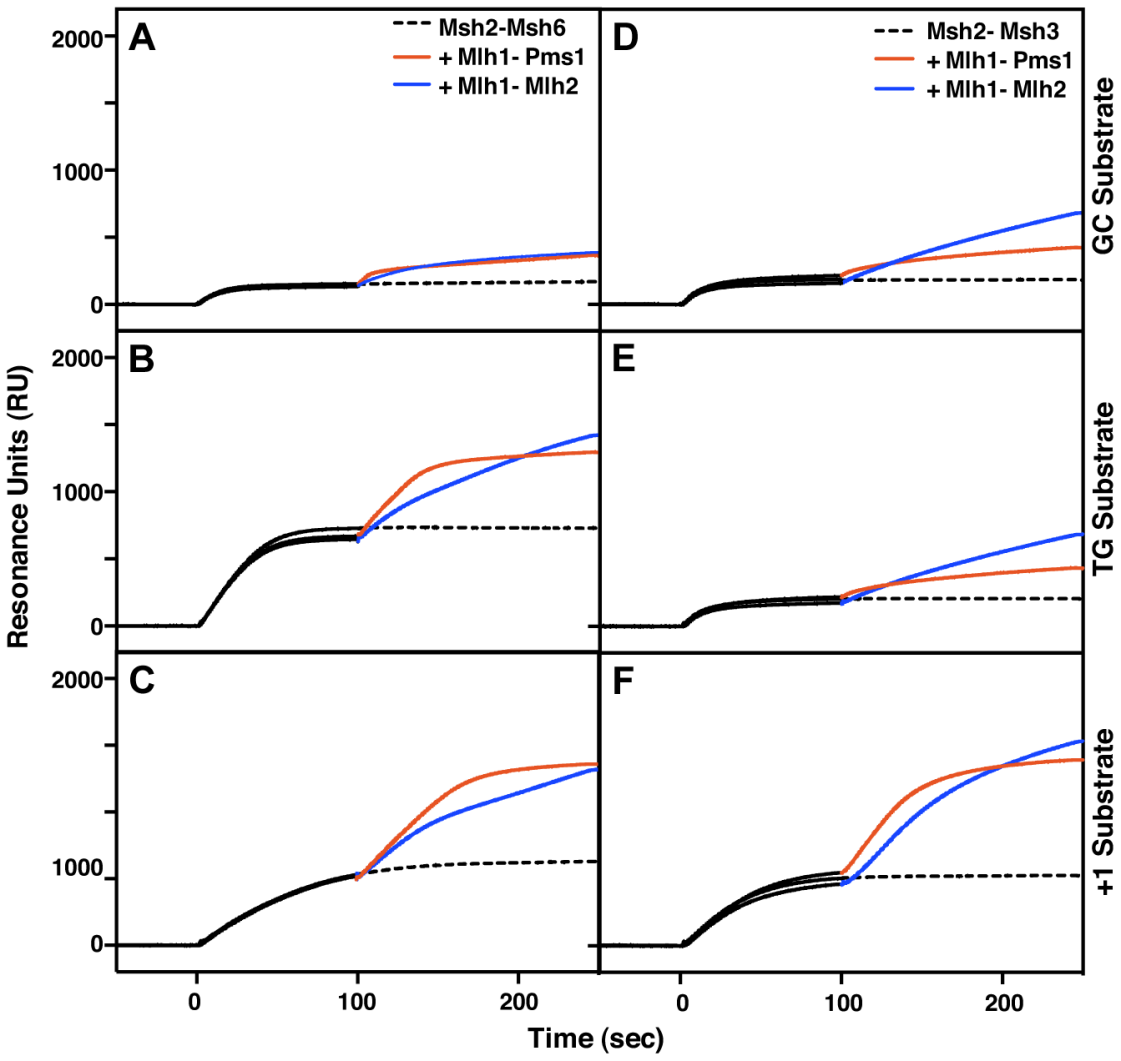
Msh2-Msh6 and Msh2-Msh3 can recruit Mlh1-Pms1 and Mlh1-Mlh2 to mispaired bases. (**A–C**) Recruitment of Mlh1-Pms1 or Mlh1-Mlh2 by Msh2-Msh6 to (**A**) homoduplex DNA, (**B**) DNA containing a central TG mispair, and (**C**) DNA containing a central +T insertion. (**D–F**) Recruitment of Mlh1-Pms1 or Mlh1-Mlh2 by Msh2-Msh3 to (**D**) homoduplex DNA, (**E**) DNA containing a central TG mispair, and (**F**) DNA containing a central +T insertion. Binding of Msh2-Msh6 or Msh2-Msh3 was monitored by surface plasmon resonance for 100 seconds (black lines), following which Mlh1-Pms1 (orange lines) or Mlh1-Mlh2 (blue lines) or no MutL homolog (dashed black line) was included in the binding reaction. Increases in resonance units (RU) indicate the binding of the proteins to the DNA on the chip. The curves shown were obtained after subtraction of the signals from the reference flow cell as well as the signals obtained by Mlh1-Pms1 or Mlh1-Mlh2 binding to the DNA alone, which did not exceed 10 to 20% of the signal attributable to recruitment of Mlh1-Pms1 or Mlh1-Mlh2 by Msh2-Msh6 or Msh2-Msh3.

The genetics of Mlh2 foci formation suggested that Mlh1-Mlh2 is primarily recruited to mispair-containing DNA by Msh2-Msh6 (Figure 2A), whereas the genetics of frameshift mutation reversion suggested that *MLH2* functions primarily in an *MSH3*-dependent pathway (Table 1A), suggesting that Mlh2 can function in conjunction with either Msh2-Msh6 or Msh2-Msh3 depending on the assay tested. To address this possibility, we purified the *S. cerevisiae* Mlh1-Mlh2 complex and tested its ability to be recruited to mispair-bound Msh2-Msh6 or Msh2-Msh3 using a previously developed surface plasmon resonance assay [48]. As previously demonstrated [48], Msh2-Msh6 has low affinity for a substrate lacking a mispair (the ‘GC’ substrate) and a higher affinity for substrates with a central T:G mispair (the ‘TG’ substrate) or a +T insertion (the ‘+1’ substrate) (Figure 4A–C). As expected, Mlh1-Pms1 readily bound Msh2-Msh6 on all of these substrates and the increase in resonance units correlated with the amount of pre-bound Msh2-Msh6. Msh2-Msh6 also recruited Mlh1-Mlh2 and, similar to Mlh1-Pms1, the increase in resonance units correlated with the amount of pre-bound Msh2-Msh6 (Figure 4A–C). However, the kinetics of Mlh1-Mlh2 recruitment differed from Mlh1-Pms1 in that initial binding was slower and the binding failed to saturate. Msh2-Msh3 had a low affinity for both the GC and TG substrates, but bound well to the +1 substrate (Figure 4D–F), consistent with the function of *MSH3* in the repair of insertion/deletion mispairs and an inability to function in the repair of many kinds of base-base mispairs [46], [49], [50]. As seen with Msh2-Msh6, both Mlh1-Pms1 and Mlh1-Mlh2 were recruited to substrates bound by Msh2-Msh3 with the level of recruitment correlating with the amount of Msh2-Msh3 bound (Figure 4D–F). The ability of Msh2-Msh3 to recruit Mlh1-Pms1 was consistent with the fact that Pms1 foci form in *msh6Δ* and *msh3Δ* strains but not in *msh2Δ* and *msh3Δ msh6Δ* strains and with our previous study demonstrating the recruitment of Mlh1-Pms1 to mispair-containing DNA by Msh2-Msh3 *in vitro* [33], [51]. Overall, these results support the idea that Mlh1-Mlh2 can function in conjunction with both Msh2-Msh3 and Msh2-Msh6, although the extent of involvement of Mlh2 may depend on the assay used and hence the exact MMR substrate being acted on.

### Overexpression of Mlh2 and Mlh3, but not Pms1, causes MMR defects

**Figure 5:**
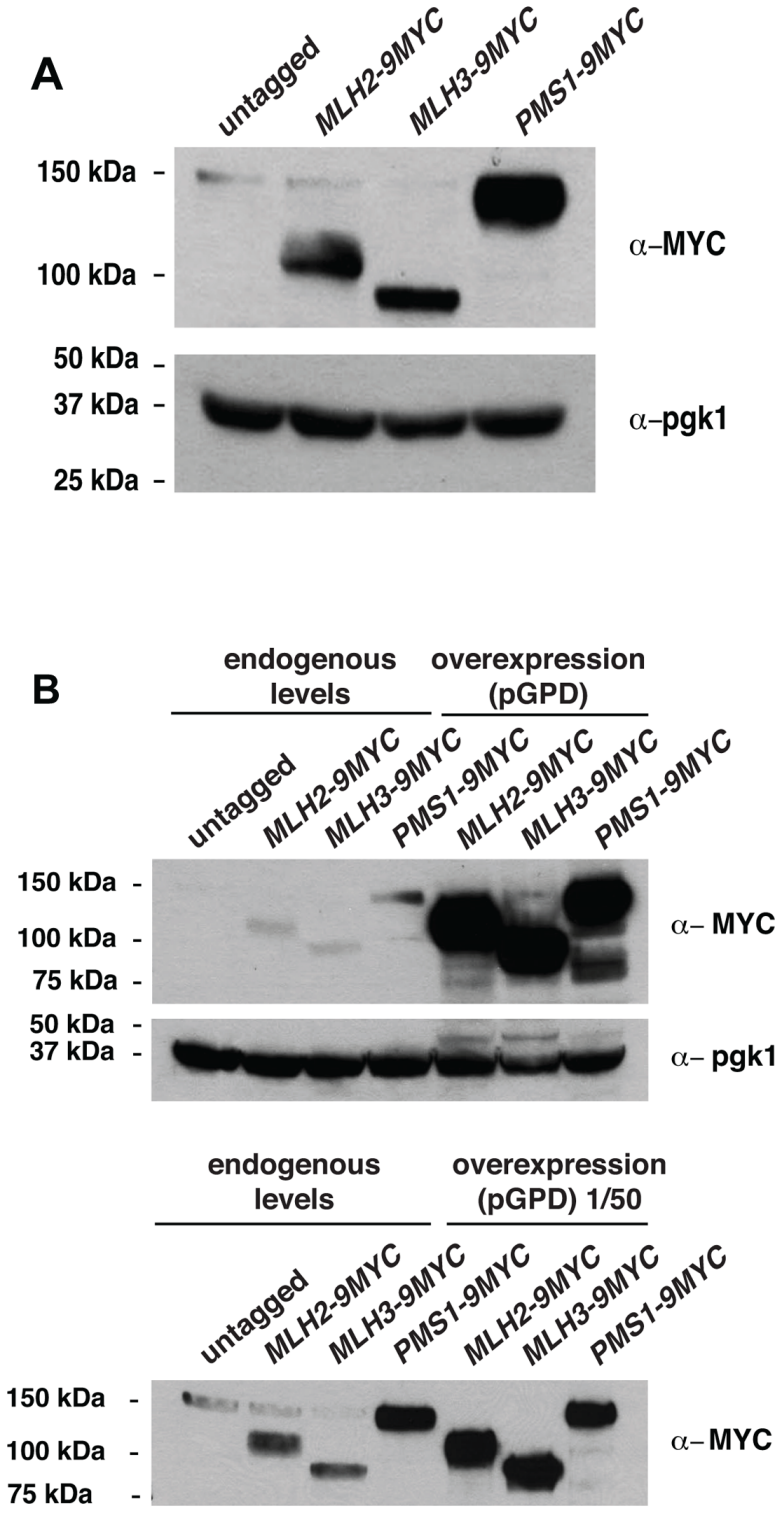
Expression of Pms1, Mlh2 and Mlh3 under control of the endogenous and *pGPD* promoters. (**A**) Expression level of Mlh2-9MYC, Mlh3-9MYC, and Pms1-9MYC driven from their endogenous promoters monitored by immunoblotting with an anti-MYC antibody. Immunoblotting with an anti-Pgk1 antibody was used a loading control. (**B**) Comparison of the levels of Mlh2-9MYC, Mlh3-9MYC, and Pms1-9MYC by immunoblotting with an anti-MYC antibody when expressed from the endogenous promoter or the *pGPD* promoter. Bottom panel, comparison after 50-fold dilution of the *pGPD* samples.

Because *MLH2* lacks conserved endonuclease motifs and mutations abolishing *pms1* endonuclease function cause a weakly dominant MMR defect that is enhanced by overexpression [13], we tested if overexpression of *MLH2* would cause an MMR defect. We therefore engineered *S. cerevisiae* strains in which the endogenous promoters of the *MLH2*, *MLH3* and *PMS1* genes were replaced by the strong promoter of the glyceraldehyde 3-phosphate dehydrogenase gene (*pGPD*) and monitored these strains for mutator phenotypes using the *hom3-10* and *lys2-10A* frameshift reversion assays and the *CAN1* forward mutation assay. Overexpression of *PMS1* did not cause increased mutation rates; however, overexpression of *MLH2* or *MLH3* drastically increased the mutation rates up to levels that were almost indistinguishable from an MMR defective strain (*msh3Δ msh6Δ*) (Table 2). Similar results were obtained upon the expression of these genes driven by their native promoters on high copy number plasmids in wild-type cells (data not shown). The endogenous expression level of Pms1 was roughly 5–10-fold higher than that of either Mlh2 or Mlh3 (Figure 5A), and the *pGPD* promoter increased the expression of each MutL homolog by >50-fold relative to the endogenous level of Pms1 (Figure 5B). The mismatch repair defect caused by the overexpression of *MLH2* was largely suppressed by the simultaneous overexpression of *PMS1* (Table 2). These data suggest that increasing the level of Mlh2 or Mlh3 by overexpression allows Mlh2 or Mlh3 to outcompete Pms1 for binding to the Mlh1 present in the cell, thereby preventing the formation of sufficient levels of Mlh1-Pms1 complex to support MMR, and that neither Mlh2 nor Mlh3 is sufficient to replace Pms1 function in MMR. In the case of Mlh1-Mlh2, this is most likely because Mlh1-Mlh2 lacks endonuclease activity. In the case of Mlh1-Mlh3, it is possible that Mlh1-Mlh3 lacks sufficient endonuclease activity to substitute for Mlh1-Pms1 or it does not function sufficiently in the Msh2-Msh6 pathway to promote MMR [21]. It is also possible that overexpression leads to much higher levels of Mlh1-Mlh2 or Mlh1-Mlh3 complexes, which then outcompete the Mlh1-Pms1 complex for a key substrate.

**Table 2:** Mutation rate analysis of strains overexpressing Pms1, Mlh2 or Mlh3.

Relevant genotype	RDKY	Mutation rate (fold increase)^a^		
		*Thr^+^*	*Lys^+^*	*Can^R^*
wild-type	5964	2.5 [1.8–3.8]×10^−9^ (1)	1.5 [1.0–5.0]×10^−8^ (1)	6.8 [3.9–8.7]×10^−8^ (1)
*pGPD-PMS1*	7897	1.0 [5.3–17.0]×10^−8^ (4)	8.7 [7.1–14.9]×10^−8^ (6)	8.4 [6.1–13.9]×10^−8^ (1)
*pGPD-MLH2*	7895	2.4 [1.6–5.4]×10^−6^ (968)	5.8 [4.5–6.9]×10^−5^ (3848)	1.6 [1.3–2.1]×10^−6^ (23)
*pGPD-MLH3*	7896	1.7 [1.1–2.6]×10^−6^ (667)	5.7 [3.4–8.3]×10^−5^ (3821)	1.3 [1.1–2.4]×10^−6^ (19)
*pGPD-(MLH2 + PMS1)*	7904	2.8 [2.0–5.7]×10^−8^ (11)	7.4 [5.3–15.9]×10^−7^ (49)	9.9 [7.9–12.2]×10^−8^ (1)
*msh3Δmsh6Δ*	6098	5.1 [2.9–13]×10^−6^ (2040)	3.4 [2.0–5.0]×10^−5^ (2267)	2.0 [0.8–5.6]×10^−6^ (29)

a Median rates of *hom3-10* and *lys2-10A* reversion and inactivation of the *CAN1* gene with the 95% C.I. in square brackets and fold increase relative to the wild-type in parentheses.

### Mlh2 is a widely conserved MutL homolog lacking endonuclease motifs

**Figure 6:**
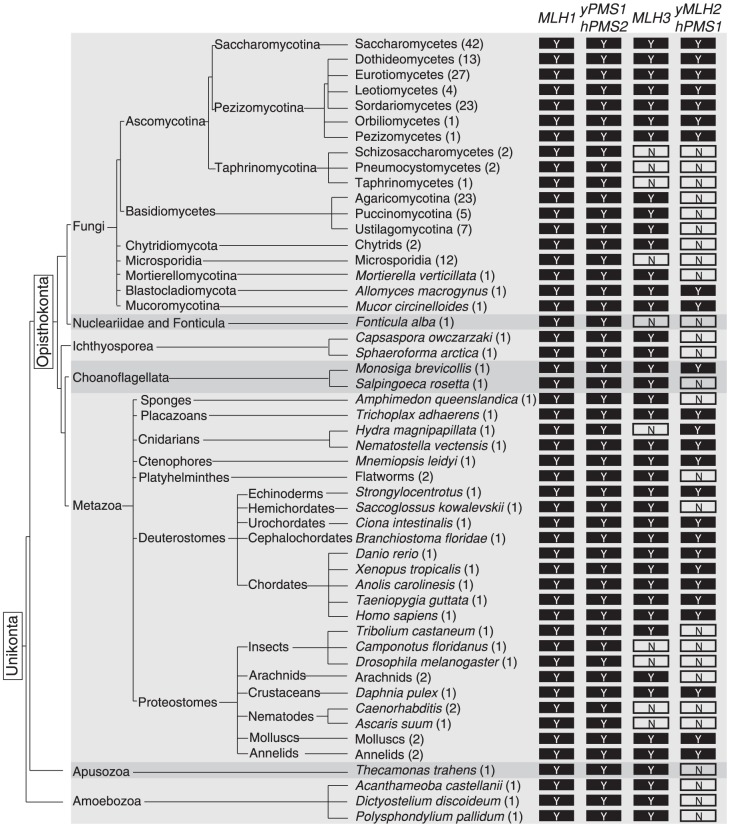
Evolutionary conservation of MutL homologs. The presence of a conserved *MLH1*, *S. cerevisiae PMS1*/human *PMS2*, *MLH3*, and *S. cerevisiae MLH2*/human PMS1 homolog in sequenced unikont genomes is indicated by a ‘Y’. ‘N’ indicates the lack of an identifiable homolog. *MLH1* and *ScPMS1*/*hPMS2* are extensively conserved, whereas *MLH3* and *ScMLH2/hPMS1* are less well conserved. Alternating light and dark grey backgrounds indicate separations between major unikont groups (Fungi, Nucleariidae and Fonticula, Ichthyosporea, Choanoflagellata, Metazoa, Apusozoa, and Amoebozoa).

Because *S. cerevisiae MLH2* plays only an accessory role in MMR, we examined the conservation of *MLH2*. We first identified MutL homologs using BLAST [52] in the Saccharomycotina subphylum of Ascomycota, which includes *S. cerevisiae*. Obvious homologs of *MLH1*, *PMS1*, *MLH3*, and *MLH2* were identified (Figure 6 and S2; Table S1) by reciprocal BLAST, by analysis of conserved synteny [53], and/or by the characteristic C-terminal sequence motifs of *MLH1*, *PMS1*, and *MLH3* involved in endonuclease activity. The origin of *MLH2* predated the whole-genome duplication that occurred ∼100 million years ago and led to *S. cerevisiae* and related yeasts [54], because *MLH2* homologs were present in species that diverged from *S. cerevisiae* prior to the genome duplication and two *MLH2* ohnologs (paralogs produced by the whole-genome duplication [55], [56]) were present in *Vanderwaltozyma polyspora*. A few clades in Saccharomycotina have lost *MLH2*, including the ‘CTG’ yeast that encode serine instead of leucine with the codon CTG [57] and species in the *Lachancea* genus (Figure S2, Table S1).

We also identified MutL homologs in other sequenced fungi (Figure 6, Table S1). *MLH1*, *PMS1*, *MLH3*, and *MLH2* genes were found in the Pezizomycotina subphylum of Ascomycota, but the early diverging Taphrinomycotina subphylum, which includes *Schizosaccharomyces pombe*, only contained *MLH1* and *PMS1*. In a small number of species, the *MLH2* homologs contained stop codons and frameshifts, which could reflect errors in the genome sequences, loss of non-essential portions of *MLH2* or inactivation of the *MLH2* homologs (Table S2). *MLH2* homologs were also not identified in Basidiomycetes but were observed in the basal fungi *Mucor circinelloides* (Mucoromycotina) and *Allomyces macrogynus* (Blastocladiomycota), which was consistent with the loss of *MLH2* in Basidiomycetes rather than the gain of *MLH2* in Ascomycetes. Phylogenetic analysis of the full-length Mlh2 protein sequences was consistent with the major divisions within fungi (Figure S3).

*S. cerevisiae MLH2* has a number of similarities to metazoan *PMS1* (note that *PMS2* in metazoans is the name for the homolog of *S. cerevisiae PMS1*). Like *S. cerevisiae MLH2*, metazoan *PMS1* lacks endonuclease motifs and does not support MMR reactions *in vitro* [58], [59], and deletion of metazoan *PMS1* causes an extremely weak mutator phenotype [60]. To examine the relationship between metazoan Pms1 and fungal Mlh2, we performed phylogenetic analysis on the sequences of the N-terminal domains of MutL homologs from select unikont species (Figure S4). This analysis identified distinct clades with strong support (clade credibility scores of 100%) for the homologous human PMS2 and fungal Pms1 proteins, human MLH3 and fungal Mlh3 proteins, as well as the human PMS1 and fungal Mlh2 proteins. Thus, this analysis suggests that human PMS1 is evolutionarily related to fungal Mlh2 and that the accessory MMR function is conserved across diverse eukaryotes (Figure 6).

## Discussion

In this study, we demonstrated that both Pms1 and Mlh2 form foci that often colocalize and that the formation of these foci depends upon Mlh1, Msh2-Msh6 and, in the case of Pms1, can also be promoted by Msh2-Msh3 [33]. The frequency of both types of foci increase in strains with increased mispair formation or defects in the downstream steps of MMR. In contrast, no Mlh3 foci were detected despite the fact that Mlh3 was expressed at levels similar to Mlh2. Deletion of *MLH2* did not cause a significant increase in the mutation rate in frameshift reversion assays unless Pms1 levels were reduced, and an *MLH2* deletion synergized with a deletion of *MSH6*, but not with a deletion of *MSH3*. *In vitro*, both Msh2-Msh6 and Msh2-Msh3 could recruit Mlh1-Mlh2 to mispair-containing DNA. These results are consistent with a role for Mlh2 in MMR. However, the lack of endonuclease motifs in Mlh2 suggests that its ability to promote MMR must involve mechanisms other than Mlh1-Mlh2-mediated cleavage of DNA. Together, these data suggest that Mlh1-Mlh2 acts as an MMR accessory or stimulatory factor that functions in conjunction with Mlh1-Pms1.

The studies performed here could be taken to present an apparent discrepancy in the placement of *MLH2* in known MMR sub-pathways. The genetics of foci formation suggest that Mlh2 recruitment is primarily mediated by Msh2-Msh6 but not Msh2-Msh3, whereas the frameshift reversion assays indicate the involvement of *MLH2* in *MSH3*-dependent but not *MSH6*-dependent MMR. In contrast, the ability of Mlh1-Mlh2 to be recruited to mispair-containing DNA *in vitro* by both Msh2-Msh6 and Msh2-Msh3 is consistent with a role for Mlh2 in both pathways. One potential explanation for this apparent discrepancy is that Mlh2 plays a role in both pathways, but the recruitment and function of Mlh1-Mlh2 in MMR are highly dependent on the type of mispair that is recognized. The relative contribution of Mlh1-Mlh2 to repair may be influenced by the sequence context of the mispair and whether repair occurs by substitution, deletion or insertion. Thus, the different assay-dependent activities of Mlh1-Mlh2 observed could reflect the fact that the three different assays used in our studies all by necessity use different mispaired substrates. Additionally, the *in vitro* mispair-dependent Mlh1-Mlh2 recruitment assays use different ratios of Msh2-Msh6 and Msh2-Msh3 than present in cells, resulting in different apparent efficiencies of recruitment of Mlh1-Mlh2 by Msh2-Msh6 and Msh2-Msh3 *in vitro* and *in vivo*. These types of differential activities have been clearly documented in the case of the Mlh1-Mlh3 complex [21]–[23], [25], [28].

We propose the following hypothesis for the role of Mlh2 in MMR. The Mlh1-Mlh2 complex may have some functional overlap with the non-endonuclease functions of the Mlh1-Pms1 complex in MMR such as recruitment of downstream MMR components and discrimination of the newly synthesized DNA strand, allowing MMR to occur at lower levels of Mlh1-Pms1 at the sites of repair. This role of Mlh1-Mlh2 in reducing the requirement for Mlh1-Pms1 while not being able to replace the activity of Mlh1-Pms1 suggests that Mlh1-Mlh2 acts as a non-essential accessory or stimulatory factor in MMR. It is also possible that Mlh1-Mlh2 in some way regulates the availability or activity of Mlh1-Pms1. Because the Mlh1-Pms1 endonuclease activity is essential for MMR *in vivo*, this hypothesis suggests that it might be possible to reveal these Mlh2 functions through the isolation of separation-of-function mutations in Pms1 that eliminate MMR in the absence of Mlh2 but leave the endonuclease activity of Mlh1-Pms1 intact. In addition, this hypothesis predicts that Mlh1-Pms1 and Mlh1-Mlh2 might be loaded onto the same DNA substrate, as suggested by the colocalization observed between Pms1 and Mlh2 foci as well as the accumulation of Mlh2 foci in the absence of Mlh1-Pms1 endonuclease activity; future studies will be required to determine if both complexes are recruited to the same mispaired substrate and if this is functionally significant. Our results are reminiscent of bacteriophage Lambda site-specific recombination where the biochemical requirement for the FIS protein during excision *in vitro*, which acts as an accessory factor, could only be revealed at sub-optimal levels of XIS protein [61].

Our studies have provided strong evidence that *S. cerevisiae* Mlh2 is the homolog of mammalian PMS1. Mammalian PMS1 is known to form a complex with MLH1, although the possible role of mammalian PMS1 in MMR is unclear [58], [60]. Consequently, the results described here may also provide new insights into a possible role of the mammalian MLH1-PMS1 complex in MMR. In addition, the ability of *S. cerevisiae* Mlh1-Mlh2 to be recruited by mispairs and the mutator phenotype caused by overexpression of *MLH2* (and *MLH3*) suggests that increased expression of human *PMS1* (or human *MLH3*) might represent a mechanism that could lead to MMR inactivation and promote tumorigenesis, analogous to MMR defects in Lynch Syndrome and other types of sporadic cancer characterized by microsatellite instability [1], [3]–[5].

## Materials and Methods

### Media and strains

*S. cerevisiae* strains were grown at 30°C in yeast extract-peptone-dextrose media (YPD) or appropriate dextrose-containing synthetic dropout media for selection of Lys^+^ or Thr^+^ revertants or canavanine-resistant (Can^R^) mutants. All strains used for mutation analyses in this study (Table S3) were derivatives of the S288c strain RDKY3686 (*MATα ura3-52 leu2Δ1 trp1Δ63 his3Δ200 hom3-10 lys2-10A*) [62]. Strains used for the colocalization experiments with the inducible *I-SceI*-generated double strand break contained the *pGAL-I-SceI* construct, the *I-SceI_cs_* restriction site adjacent to the *3xURA3-tetOx112* array, and *TetR-mRFP* derived from W9561-17A [63] (generously provided by R. Rothstein, Columbia Medical School).

Gene deletion, tagging and promoter replacements for gene overexpression (*pGPD*) were performed using standard PCR-based recombination-mediated gene targeting methods [64] followed by confirmation with PCR. The correct insertion of tags/promoters and the absence of additional mutations were confirmed by sequencing.

Strains expressing Pms1 under the tetracycline repressible promoter (*tetO_2_*) were generated as previously described [47]. The parental strain (RDKY8158) containing the chimeric repressor (*tetR′-SSN6*), the transactivator (tTA) and the MMR reporters (*lys2-10A* and *hom3-10*) was obtained after mating RDKY3686 with the CML476 strain (*MATa ura3-52 leu2Δ1 his3Δ200 GAL2 CMVp(tetR′-SSN6)::LEU2 trp1::tTA* (Euroscarf). Plasmid pCM324 (Euroscarf) was used to introduce the *tetO_2_* promoter immediately upstream of the Pms1 start codon.

Specific point mutations were introduced in chromosomal genes using standard integration/excision methods and the following integrating plasmids: *msh6-F337A* was introduced with pRDK1602 [33]; *pms1-E707K* was introduced with pRDK1583 [33]; and the DNA polymerase alleles *pol2-M644G*, *pol3-01*, and *pol3-L612M* were integrated as previously described [35], [37], [65], [66]. The presence of the desired mutation and absence of additional mutations was confirmed by DNA sequencing.

For visualization of the low abundance proteins Pms1 and Mlh2, we tagged them at the C-terminus at the endogenous gene locus with four tandem copies of GFP (4×GFP) using the pSM1023 plasmid (gift of E. Schiebel, University of Heidelberg). Testing of the tagged strain RDKY7893 (*MLH2-4GFP.KanMX6*) for sensitivity to cisplatin as described previously [30] showed that it had the same sensitivity as the wild-type parental strain RDKY5964 and was more sensitive than the *mlh2Δ* control strain RDKY7926 (*mlh2::KanMX4*), indicating that the tag on the C-terminus of Mlh2 was unlikely to have an effect on Mlh2 function [30]. The nuclear pore protein Nic96 was tagged at the C-terminus with mCherry using the plasmid pBS35 as a template (Yeast Resource Center YRC). The C-terminus of Mlh2 was tagged with tdTomato using a PCR-based strategy and the plasmid pRDK1663. This plasmid was derived from pYM25 *yeGFP.hphNT1* (Ref. [64]; obtained from Euroscarf) by excising the *Hind*III-*Bgl*II fragment containing the *yeGFP* coding sequence, replacing it with a *Hind*III-*Bgl*II fragment (generated by gene synthesis at Integrated DNA Technologies, IDT) encoding an *S. cerevisiae* codon optimized tdTomato gene and a linker (Gly Ala)_5_ immediately upstream of the Met start codon of tdTomato.

### Immunoblotting

*S. cerevisiae* whole-cell extracts were prepared using the Yeast Extract Buffer (1.85 M NaOH, 7.5% beta-mercaptoethanol), and the soluble proteins were precipitated by addition of an equal volume of 50% trichloroacetic acid. The protein extracts were analyzed by SDS-PAGE using 4%–15% gradient gels (BioRad) and immunoblotting. Detection of GFP-tagged proteins was performed using the anti-GFP antibody, clone B34 (Covance). Using the anti-Pgk1 antibody (clone 22C5D8; Invitrogen), Pgk1 expression was monitored as a loading control. MYC-tagged proteins were detected with the monoclonal anti-MYC antibody (clone 4A6; Millipore).

### Genetic assays

Mutation rates were determined using the *hom3-10* and *lys2-10A* frameshift reversion assays and *CAN1* inactivation assay by fluctuation analysis [67] as previously described [46], [62]. The mutation rates presented in Table 1B were determined in the absence or presence of 10 µg/ml of doxycycline (present in liquid cultures as well as in agar plates).

### Protein purification

All the proteins were expressed from plasmid expression vectors in either *E. coli* or *S. cerevisiae* as indicated below. Typical yields ranged from 100 µg to 500 µg per liter of expressing cells. All the protein preparations were confirmed to be greater that 95% pure as judged by SDS-PAGE followed by staining of the resulting gels with Coomassie Blue.

#### Purification of Mlh1-Pms1

*S. cerevisiae* Mlh1-Pms1 was overexpressed in *S. cerevisiae* and purified according to a previously published procedure using an overexpression strain derived by the transformation of RDKY1293 (*MATα, ura3-52, leu2Δ1, trp1, his3Δ200, pep4::HIS3, prb1Δ1.6R, can1*) with the plasmids pRDK573 (*pGAL1-10-MLH1 TRP1*) and pRDK1099 (*pGAL1-10-PMS1-FLAG LEU2*) [13], [68].

#### Purification of Mlh1-Mlh2

*S. cerevisiae* Mlh1-Mlh2 was overexpressed in the *S. cerevisiae* strain RDKY8153 that was generated by transformation of RDKY1293 (*MATα, ura3-52, leu2Δ1, trp1, his3Δ200, pep4::HIS3, prb1Δ1.6R, can1*) with the plasmids pRDK573 (*pGAL1-10-MLH1 TRP1*) and pRDK1664 (*pGAL1-10-Mlh2-FLAG LEU2*). pRDK1664 was generated by replacement of the *PMS1* ORF with *MLH2* using gap-repair. Briefly, pRDK1099 was linearized with *Sph*I and used to co-transform wild-type *S. cerevisiae* with a PCR product containing the *MLH2* ORF and flanking sequences homologous to the *pGAL1-10* promoter and FLAG at the 5′ and 3′ end, respectively. Gap-repaired plasmids were recovered from Leu^+^ transformants and verified by sequencing.

The overexpressing strain RDKY8153 was grown as previously described for the purification of Mlh1-Pms1 [69]. A 100 ml culture was harvested by centrifugation for 10 min at 3,000 rpm in a swinging bucket rotor in a Sorvall Legend RT centrifuge at 20°C and then the cells were resuspended in 25 ml of Buffer A_200_ [50 mM Tris-Cl (pH 8.0), 10% glycerol, 200 mM NaCl, 2 mM β-mercaptoethanol, protease inhibitor mixture PIC D (final concentrations of 1 mM phenylmethanesulfonyl fluoride (PMSF), 1 µg/L chymostatin, and 1 µg/L pepstatin A) and protease inhibitor mixture PIC W (final concentrations of 1 mM benzamidine, 0.5 µg/L bestatin, 1 µg/L aprotinin, and 1 µg/L leupeptin)] at 4°C. Then, 4 ml of Cell Lytic Y Cell Lysis Reagent (Sigma) was added to the resuspended cells, and the cells were distributed into 1 ml aliquots in microcentrifuge tubes. The tubes were rocked for 45 min at 4°C, and then the cells were sonicated for 25 s with a 5-s pulse on and a 1-s pulse off for three cycles. The tubes were then centrifuged at 14,000 rpm in a tabletop Eppendorf centrifuge for 30 min at 4°C, and the supernatants were pooled. Then, 4 ml of FLAG antibody resin equilibrated in Buffer A_200_ was added to the pooled supernatant, and the resulting suspension was rocked for 1 hr at 4°C. The resin was then poured into a column that was washed 5 times with 1 ml of buffer A_200_, and then the protein was washed 7 times with 1 ml of buffer A_200_ containing 200 µg/ml of FLAG peptide. Fractions containing the protein were pooled and concentrated as previously described [13], and then aliquots were frozen in liquid nitrogen and stored at −80°C.

#### Purification of Msh2-Msh6

*S. cerevisiae* wild type Msh2-Msh6 was overexpressed in *E. coli* BL21 (DE) RIL using the pET11 *MSH2-MSH6* plasmid and purified according to a previously published procedure [70], [71].

#### Purification of Msh2-Msh3

*S. cerevisiae* Msh2-Msh3 was expressed in *S. cerevisiae* RDKY2418 *MATα, ura3-52, leu2Δ1, trp1, his3Δ200, pep4::HIS3, prb1Δ1.6R, can1, msh2::hisG, msh6::hisG* [72] transformed with the expression plasmids pRDK354 (p*GAL1-10-MSH2 URA3*) and pRDK1596 (p*GAL1-10 MSH3-FLAG LEU2*) and purified as described previously [51].

### Surface plasmon resonance analysis

The recruitment of Mlh1-Pms1 or Mlh1-Mlh2 by Msh2-Msh6 or Msh2-Msh3 bound to DNA was analyzed using a Biacore T100 instrument essentially as described previously [13], [68]. Biotinylated 236 bp-long double-stranded DNAs containing the terminal *lacO* sequence and a central TG mispair, +T insertion or GC base pair were conjugated to 3 flow cells of a streptavidin-coated Biacore SA chip (GE Healthcare) to obtain a signal of ∼100 Resonance Units (RU). The signal from the unmodified flow cell was used for reference subtraction in all experiments. A constant flow rate of 20 µl/min was maintained and experiments were performed at 25°C. First, 30 nM purified LacI tetramer (a gift from Kathleen Matthews, Rice University) in reaction buffer (25 mM Tris-Cl (pH 8.0), 4 mM MgCl_2_, 110 mM NaCl, 0.01% Igepal, 2 mM DTT and 2% glycerol) was injected over the chip for 60 s. Next, at time t = 0 a sample containing 30 nM LacI, 20 nM Msh2-Msh6 or 20 nM Msh2-Msh3 and 250 µM ATP in reaction buffer was injected for 100 s, followed by the immediate injection of a sample containing the same mixture and in addition 40 nM Mlh1-Pms1 or 40 nM Mlh1-Mlh2, or no MutL homolog for 150 s. The chip surface was regenerated using a 60 s pulse of 2 M NaCl. Control experiments were performed in which the first injection consisted of 30 nM LacI, the sond injection consisted of 30 nM LacI and 250 µM ATP, and the third injection consisted of 30 nM LacI, 250 µM ATP and either 40 nM Mlh1-Pms1 or 40 nM Mlh1-Mlh2. These data were subtracted from the data obtained using Msh2-Msh6/Msh2-Msh3 in the second and third injections, and the subtracted curves are presented. Data were analyzed using the BiaEvaluation 2.0.3 (GE Healthcare) and Prism 6.0 software (GraphPad).

### Live-cell imaging and image analysis

Exponentially growing cultures were washed and resuspended in water and placed on minimal media agar pads, covered with a coverslip, and sealed with valap (a 1∶1∶1 mixture of Vaseline, lanolin, and paraffin by weight). Cells were imaged on a Deltavision (Applied Precision) microscope with an Olympus 100×1.35NA objective. Fourteen 0.5 µm z sections were acquired and deconvolved with softWoRx software. For time-lapse imaging of Pms1 foci, images were collected every min with fewer z sections to minimize photobleaching. Further image processing, including maximum intensity projections and intensity measurements, was performed using ImageJ.

For drug treatments, cells that were growing logarithmically in YPD medium were treated with either 200 mM hydroxyurea or 5 µM phleomycin for 3 hr and prepared for microscopy as described above. Cell cycle arrest was confirmed by examining cell morphology using a microscope. For DSB induction by I-*Sce*I, strains RDKY7906, RDKY7907, and RDKY7908 were grown overnight in medium containing 2% raffinose. The cultures were diluted into the same medium and after 3 hr, pelleted and resuspended in medium containing either 2% raffinose or 2% galactose, incubated at 30°C for 2.5 hr, and prepared for microscopy.

Foci were considered to be colocalized if over half of their diameters overlapped. Colocalization was scored if at least one focus per nucleus displayed colocalization in the same z section. Images with the same fluorescent fusion protein in the same figure have identical contrast adjustment. The data presented here contain representative images and quantitative data from at least two independent experiments, each performed using two independent strain isolates, which gave similar results. The total number of cells/nuclei (n) analyzed for each strain is indicated.

### Phylogenetic analysis

MutL homologs were identified using Protein BLAST [52] against the non-redundant protein sequences (nr) database. For some MutL homologs, alignments of the protein sequences revealed that strongly conserved regions were missing. We analyzed the genomic sequences encoding these genes and discovered that these were often due to incorrect assignment of exons, as the missing regions tended to either be at exon boundaries, suggesting the inappropriate identification of a predicted splice site, or in introns, suggesting the failure to identify an exon. For these genes, we re-annotated the exons, typically merging in-frame introns with the surrounding exons or identifying missing exon sequences, and retranslated the protein sequences (Table S2). The criteria for re-annotation of the exons were to improve homology to the conserved portions of the protein sequence alignment and maintain conservation of the intron/exon structure with closely related species. In some cases, a frameshift or stop codon was present in an exon. In these cases, the surrounding protein sequence was translated to prevent truncations from having an inordinately large effect on the phylogenetic analysis; however, this analysis could not distinguish between sequencing errors or species having mutations that inactivated the gene. The cases in which a protein was translated in spite of frameshifts or stop codons in the reference genomic sequence are explicitly labeled in Table S2. To decipher relationships between MutL homologs, amino acid sequences of the N-terminal domains were aligned with MAFFT [73] to avoid misalignments due to effects of the rapidly evolving and likely unstructured linker between the MutL N- and C-terminal domains. Phylogenetic analyses were performed with MrBayes [74] using the mixed amino acid rate matrices model and 1,000,000 generations. Clade credibility values above 75 were considered significant.

## Supporting Information

Figure S1 Downregulation of *S. cerevisiae* Pms1 expression upon addition of doxycycline. Expression levels of Pms1 (tagged with 9MYC) of strains containing the repressible *tetO_2_-Pms1* promoter (lanes 1+2) or the endogenous Pms1 promoter (lanes 3+4). Cells were grown for 3 hours in YPD in the presence (or absence) of 10 µg/ml of doxycycline, as indicated. Whole-cell extracts were prepared and analyzed by SDS-PAGE and immunoblotting. The anti-Myc antibody was used to detect Pms1 expression and Pgk-1 was used as a loading control. Relates to Table 1.
(EPS)

Figure S2 Conservation of *MLH1*, *MLH2*, *MLH3*, and *PMS1* in species of Saccharomycotina with sequenced genomes. ‘WGD clade’ indicates the group of species that have undergone whole-genome duplication. ‘CTG clade’ indicates the group of species that encode serine instead of leucine with the CTG codon. Phylogenetic relationships between species were derived from previously published analyses [75]–[77].
(EPS)

Figure S3 Clustering of the full-length Mlh2 sequences from fungi. This phylogenetic analysis reconstructed the Saccharomycotina and Pezizomycotina subphyla within Fungi as well as class and family relationships, consistent with homology between fungal *MLH2* genes. Clade support values of 75 or higher are indicated on the phylogenetic tree.
(EPS)

Figure S4 Clustering of sequences of the N-terminal domains of MutL homologs from sequenced unikont species provided clear support for the homology of *S. cerevisiae MLH2* and human *PMS1*, *S. cerevisiae MLH3* and human *MLH3*, as well as *S. cerevisiae PMS1* and human *PMS2*. Clade support values of 75 or higher are indicated on the phylogenetic tree.
(EPS)

Table S1 MutL homologs in sequenced unikont genomes.
(XLS)

Table S2 Re-annotation of MutL genes in fully sequenced genomes.
(DOCX)

Table S3 *S. cerevisiae* strains used in this study.
(DOCX)
