# Supplementary material for: Mlh2 Is an Accessory Factor for DNA Mismatch Repair in Saccharomyces cerevisiae
Source: PLoS Genet. 2014 May 8;10(5):e1004327. doi: 10.1371/journal.pgen.1004327 (PMC4014439; doi:10.1371/journal.pgen.1004327)
Supplement: Table S2 — Re-annotation of MutL genes in fully sequenced genomes. (DOCX) [file pgen.1004327.s006.docx]

**Table S2.** Reannotation of MutL genes in fully sequenced eukaryotic genomes.

| ***Accession*** | ***Field*** | ***Value*** |
| --- | --- | --- |
| *Ajellomyces capsulatus* G186AR EEH05069.1 (Mlh2, HCBG_07020) | | |
|  | old | /coded_by=”join(GG663372.1:1097817..1098175,GG663372.1:1098291..1098697,GG663372.1:1098749..1100712)” |
|  | new | /coded_by=”join(GG663372.1:1097817..1098223,GG663372.1:1098291..1098697,GG663372.1: 1098749..1100712)” |
|  | old | /sequence=”MSIAQLPPDTIRAVRSNSVLSDPCALAKELIDNSLDAGATCISVEVAANTLDTIQVKDNGTGIHPNDRPLVCKRSCTSKLRSIDDLENIGGSTLGFRGEALASAAEMCGNIAITTRVSGDCTKASHPTGTTVRVYEFLRFVPVRKQRALKSASKTLNRMKKVLNAYVLSRPEIRLSFKILKSKHDSSWIYASNKHPTVSTAMLRVIGAEAISQCTTITWPQIQDERDDSLPGSVERSESIHPVIRLVATIPKPCSDFSSINYAGQYVSIDRRPMSTSNGVAKELAELFKSHIRSGSLCDSGRTPPVDPFCFIHLHCRPGIYDVNVEPLKNDVLFEDSKLVMSIAGNIFKAVYGEIKPRHTHQRNCQIENQPSSSRMIRPLDKRSANNQRDHEVEESPLFRHISSSATKDCHSIERICASPSEAKKDNMSAGSFVDTSACPRAAESNLSLLNSSLLTNPWMLAKGQPVTPRKEDTQSRDLNSQLLTPAYEDGRSRLDEKRIKNCEANSVLAGPPYPIPLSTRTLETRLVEGDYDKSSTDVRKPQMSELCNVPQLSASHKSKQRKSPGVLGSLDAWIRNCRDAPSPSTEESQVVGILQQSEPDGNELTETDIAGRFGKEDNAAATSRSHGDFSAGLSPHTSLNALGIHLNSAHTNAKSHAQPLAISQEEDDDPDYRIHGSTDNRLTSPSHEMVAGALDFEYRKKTAIQLHRKRQQQQQQQQQQQQQVSTQSALHPWVGANNAKISQGSPHQNRYRRAKAHLVNQLPELEFPRHEITFSPSKMGSEDAREYLMRQNWPTQIGFKSRRILTNQLPLEVIPSASALHGLALSWTKQDDEASIPNKELLKIDGYVTSGIVPPPSAFFEVNPADINIWTTRLLALIQEQYRSEILDEDWRIQLVFDAIPTSHARAS” |
|  | new | /sequence=”MSIAQLPPDTIRAVRSNSVLSDPCALAKELIDNSLDAGATCISVEVAANTLDTIQVKDNGTGIHPNDRPLVCKRSCTSKLRSIDDLENIGGSTLGFRGEALASAAEMCGNIAITTRVSGELVGETLKYDRTGLLISCTKASHPTGTTVRVYEFLRFVPVRKQRALKSASKTLNRMKKVLNAYVLSRPEIRLSFKILKSKHDSSWIYASNKHPTVSTAMLRVIGAEAISQCTTITWPQIQDERDDSLPGSVERSESIHPVIRLVATIPKPCSDFSSINYAGQYVSIDRRPMSTSNGVAKELAELFKSHIRSGSLCDSGRTPPVDPFCFIHLHCRPGIYDVNVEPLKNDVLFEDSKLVMSIAGNIFKAVYGEIKPRHTHQRNCQIENQPSSSRMIRPLDKRSANNQRDHEVEESPLFRHISSSATKDCHSIERICASPSEAKKDNMSAGSFVDTSACPRAAESNLSLLNSSLLTNPWMLAKGQPVTPRKEDTQSRDLNSQLLTPAYEDGRSRLDEKRIKNCEANSVLAGPPYPIPLSTRTLETRLVEGDYDKSSTDVRKPQMSELCNVPQLSASHKSKQRKSPGVLGSLDAWIRNCRDAPSPSTEESQVVGILQQSEPDGNELTETDIAGRFGKEDNAAATSRSHGDFSAGLSPHTSLNALGIHLNSAHTNAKSHAQPLAISQEEDDDPDYRIHGSTDNRLTSPSHEMVAGALDFEYRKKTAIQLHRKRQQQQQQQQQQQQQVSTQSALHPWVGANNAKISQGSPHQNRYRRAKAHLVNQLPELEFPRHEITFSPSKMGSEDAREYLMRQNWPTQIGFKSRRILTNQLPLEVIPSASALHGLALSWTKQDDEASIPNKELLKIDGYVTSGIVPPPSAFFEVNPADINIWTTRLLALIQEQYRSEILDEDWRIQLVFDAIPTSHARAS” |
| *Ajellomyces capsulatus* G186AR EEH10117.1 (Mlh1, HCBG_01758) | | |
|  | old | /coded_by=”join(GG663364.1:1616903..1617004,GG663364.1:1617449..1617715,GG663364.1:1617784..1697709)” |
|  | new | /coded_by=”join(GG663364.1:1616903..1617004,GG663364.1:1617144..1617323,GG663364.1:1617449..1617715,GG663364.1:1617784..1619709)” |
|  | old | /sequence=”MADEDMDVDSPDPRGTKRTAAEAGLPPQPSRKIQCDDLGILCERFTTSKLKAFEDLSSIGTYGFRGEALASISHIAHLTVTTKTAGSSCAWRAHYSDGKLVPAKPGQTAEPKPTAGRGGTQITVEDLFYNVPTRRRAFRSSSEEYAKILDVVGRYAVHCSGVAFSCKKHGDSGVSISTSTNSTTVDRIRQIHGSAVASELIDFSVDDPRLGFRASGWASNANYHAKRTTILLFINHRSVESSAVRRAIEQAYSNFLPKGGHPFAYLDLEIEPQRVDVNVHPTKREVNFLNEDEIIGSICTTIQQKLATVDSSRTFMVQTLLPVGRTQSASVNSNAEPPFDDRQPTPRTLSGTKRPYENNLVRTDASMRKITSMLPPATTSQARPILNGNSQTLPLEEDGLRYESTGREPTQIRLSSVKSLRAAVRSSMHNNLTELFASLTYVGLVDERRRIAAIQSGVKLFLVDYGMISSEFFYQVGLTDFGNFGKINLESSPRLVDLLSLAAATERDEYRRQQSASAAAAATKEQSAAADIESVDFDRIVTTIATQLIERREMLDEYFSLTISEDGNLLSIPLLLKGYMPSLAKLPRFLLRLGPYVDWTDEEACFRTFLRELAAFYTPEQLPIAPLIVPSSSNKNNNSAPPPTTKPAARKSETETATSSQSTTSQSHPSPSSSPSSSSESHPSNPNEPAQTPPTNSPSNPPKQPSQPQREDEAIVRRRAQLSWTLEHVLFPSLRARLVATNDLVRGVVEVADLKGLYRVFERC” |
|  | new | /sequence=”MADEDMDVDSPDPRGTKRTAAEAGLPPQPSRKIQALDPDVVNKIAAGEIIVAPMHALKELIENSVDAGATSVEILVKDGGLKLLQITDNGHGIDCDDLGILCERFTTSKLKAFEDLSSIGTYGFRGEALASISHIAHLTVTTKTAGSSCAWRAHYSDGKLVPAKPGQTAEPKPTAGRGGTQITVEDLFYNVPTRRRAFRSSSEEYAKILDVVGRYAVHCSGVAFSCKKHGDSGVSISTSTNSTTVDRIRQIHGSAVASELIDFSVDDPRLGFRASGWASNANYHAKRTTILLFINHRSVESSAVRRAIEQAYSNFLPKGGHPFAYLDLEIEPQRVDVNVHPTKREVNFLNEDEIIGSICTTIQQKLATVDSSRTFMVQTLLPVGRTQSASVNSNAEPPFDDRQPTPRTLSGTKRPYENNLVRTDASMRKITSMLPPATTSQARPILNGNSQTLPLEEDGLRYESTGREPTQIRLSSVKSLRAAVRSSMHNNLTELFASLTYVGLVDERRRIAAIQSGVKLFLVDYGMISSEFFYQVGLTDFGNFGKINLESSPRLVDLLSLAAATERDEYRRQQSASAAAAATKEQSAAADIESVDFDRIVTTIATQLIERREMLDEYFSLTISEDGNLLSIPLLLKGYMPSLAKLPRFLLRLGPYVDWTDEEACFRTFLRELAAFYTPEQLPIAPLIVPSSSNKNNNSAPPPTTKPAARKSETETATSSQSTTSQSHPSPSSSPSSSSESHPSNPNEPAQTPPTNSPSNPPKQPSQPQREDEAIVRRRAQLSWTLEHVLFPSLRARLVATNDLVRGVVEVADLKGLYRVFERC” |
| *Ajellomyces capsulatus* NAm1 XP_001538667.1 (Mlh2, HCAG_06272) | | |
|  | old | /coded_by=”complement(join(NW_001813976.1:2165662..2167195,NW_001813976.1:2167370..2167673,NW_001813976.1:2167849..2168173))” |
|  | new | /coded_by=”complement(join(NW_001813976.1:2165662..2167241,NW_001813976.1:2167293..2167669,NW_001813976.1:2167767..2168173))” |
|  | old | /sequence=”MSIAQLPPDTIRAVRSNSVLSDPCALAKELIDNSLDAGATCISVEVAANTLDTIQVKDNGTGIHPNDRHLVCKRSCTSKLRSIDDLENIGGSTLGFRGEALASAAEMCGTTVRVDEFLRFVPVRKRRALKSASKTLNRMKKVLNAYVLSRPEIRLSFKILKRKNDSSWIYASNKHPTVSTAMLRVIGAEAISQCTTITWPQIQDERDDSLRPMSTSNGVAKELVELFKSHIRSGSLCDSGQTPPVDPFCFIHLHCRPGIYDVNVEPLKNDVLFEDSKLVISMAGNLFKTVYGETKPRHTHQRNCQIENQPSSSRMIRPLDSRPANNQRDHEVEESPLFRHISSSATKDCHSIECICASSSKAKKDNMLAGSCVDTYACPRAAESNLSLLNSSLLTNPWLLAKGQHVTPRKEDAQSRDLNSQLLTPAYQDGRSRLDEKRIKNCGANSVFAGPPYPTPVSTRTLETRLVEGDNDKSSTDVRKPQTSELCNVPQLTASHKSKQRKSSGVLGSLDAWIRNCRDAPSHSTEESHAVGILQQSEPDGNELTETDIAGRFGKEDNAVTTSLSHGDFSAGLSPRTSLNALGIHLNSAHTNTKNHCQPLAISQEEDDDPDYRIHRLTDNRPTSRPTSPSHEMIAEALDFEYRKKTAIQLHRKRQQQQQQQQQQQISTQSTLHSWAGANNAKISQGSPHQNRYRRAKAHLVNQLPELELPRHEITSRRPK” |
|  | new | /sequence=”MSIAQLPPDTIRAVRSNSVLSDPCALAKELIDNSLDAGATCISVEVAANTLDTIQVKDNGTGIHPNDRHLVCKRSCTSKLRSIDDLENIGGSTLGFRGEALASAAEMCGNIAITTRVSGELAGETLKYDRTGLLIRCTKASHPTGTTVRVDEFLRFVPVRKRRALKSASKTLNRMKKVLNAYVLSRPEIRLSFKILKRKNDSSWIYASNKHPTVSTAMLRVIGAEAISQCTTITWPQIQDERDDSLSESVESNESIHPVIRLVATIPKPCSDFSAINYAGQYVSIDRRPMSTSNGVAKELVELFKSHIRSGSLCDSGQTPPVDPFCFIHLHCRPGIYDVNVEPLKNDVLFEDSKLVISMAGNLFKTVYGETKPRHTHQRNCQIENQPSSSRMIRPLDSRPANNQRDHEVEESPLFRHISSSATKDCHSIECICASSSKAKKDNMLAGSCVDTYACPRAAESNLSLLNSSLLTNPWLLAKGQHVTPRKEDAQSRDLNSQLLTPAYQDGRSRLDEKRIKNCGANSVFAGPPYPTPVSTRTLETRLVEGDNDKSSTDVRKPQTSELCNVPQLTASHKSKQRKSSGVLGSLDAWIRNCRDAPSHSTEESHAVGILQQSEPDGNELTETDIAGRFGKEDNAVTTSLSHGDFSAGLSPRTSLNALGIHLNSAHTNTKNHCQPLAISQEEDDDPDYRIHRLTDNRPTSRPTSPSHEMIAEALDFEYRKKTAIQLHRKRQQQQQQQQQQQISTQSTLHSWAGANNAKISQGSPHQNRYRRAKAHLVNQLPELELPRHEITSRRPK” |
| *Ajellomyces dermitidis* ER-3 EEQ88053.1 (Mlh2, BDCG_03173) | | |
|  | old | /coded_by=”complement(join(EQ999975.1:4323636..4325353,EQ999975.1:4325646..4326052,EQ999975.1:4326123..4326529))” |
|  | new | /coded_by=”complement(join(EQ999975.1:4323636..4325593,EQ999975.1:4325646..4326052,EQ999975.1:4326123..4326529))” |
|  | old | /sequence=”MSIAHLPPDTARAVRSTSAISDPCSLVKELIDNSLDAGATYISVEVSANTLDTIHVKDNGVGIHPNDRHLVCKRSCTSKLQTIDDLKNIGGSTLGFRGEALASAAEMCGSIAITTKVAGEMVGETLKYDRTGLLVSCTKAAHPTGTAVRISEFLKLVPVRKQIALKSAPKTLNQMKKVLNAYVLSRPEIRLSFKILKTKSDSSWVYASTENATVSTAMLRVVGAEAISQCTTITWPQIQGGDDDFLLESLDINESNPSAIRIVATIPKPGSDSKLVMSTAENLFKTVYGEIESKHTHQNNRQLEDQGLSPQVIRSPRSRSASSRRTHEAEDQPLFLHIPSHAVKHCHSIGNALVSSTQGKKDNVSVASSADTTSCPQLAENNPTLSDSSRLTNPWMLAKRQYVTPPKKNAQSRNFNSHLLTPAYEDESFRSDSTMVDNCTTNSLPASPANLTPIPMQTPETRLAKAGHNISPTGVSKSQMNALSSLSEPSVSHGGTQRVLGGSGSLDVWIRNPRDAANHPADEGQDIGIPRDREDCENRLPEIAIARRFGKEGNGVSTGRSHAYSPAGLSPRALLSSPGIHRNSTLSNTRGYRPPSISFEVDDDQNYRIRESTDNQNSTPEGVIGEALDFEYRKKAATRLYRQLQQQQEQHQMPAQPMLLSWAGANHVKTSQSSPHQNRYSKAKADLVSHSPRPKLLSHEMAFLVPEMDSEDPREYFRRHKDDLSHHASTHIGVKNKRILTNQLPLEVIPNAFALHGLALNWSRKDDVASILGNELFKADEYIRKGIVPPSPAFLEVQPTDISLWTTRLSTLTQKQYRSEHVGGEVGTKLVFDTVPMSHASAP” |
|  | new | /sequence=”MSIAHLPPDTARAVRSTSAISDPCSLVKELIDNSLDAGATYISVEVSANTLDTIHVKDNGVGIHPNDRHLVCKRSCTSKLQTIDDLKNIGGSTLGFRGEALASAAEMCGSIAITTKVAGEMVGETLKYDRTGLLVSCTKAAHPTGTAVRISEFLKLVPVRKQIALKSAPKTLNQMKKVLNAYVLSRPEIRLSFKILKTKSDSSWVYASTENATVSTAMLRVVGAEAISQCTTITWPQIQGGDDDFLLESLDINESNPSAIRIVATIPKPGSDLSKINHAGQYVSIDGRPMSISNGVGKELVKLFKSYLRSGSHCVGSQTSLVNPFLFMHLHCQPGSYDVNVEPSKNDVVFEDSKLVMSTAENLFKTVYGEIESKHTHQNNRQLEDQGLSPQVIRSPRSRSASSRRTHEAEDQPLFLHIPSHAVKHCHSIGNALVSSTQGKKDNVSVASSADTTSCPQLAENNPTLSDSSRLTNPWMLAKRQYVTPPKKNAQSRNFNSHLLTPAYEDESFRSDSTMVDNCTTNSLPASPANLTPIPMQTPETRLAKAGHNISPTGVSKSQMNALSSLSEPSVSHGGTQRVLGGSGSLDVWIRNPRDAANHPADEGQDIGIPRDREDCENRLPEIAIARRFGKEGNGVSTGRSHAYSPAGLSPRALLSSPGIHRNSTLSNTRGYRPPSISFEVDDDQNYRIRESTDNQNSTPEGVIGEALDFEYRKKAATRLYRQLQQQQEQHQMPAQPMLLSWAGANHVKTSQSSPHQNRYSKAKADLVSHSPRPKLLSHEMAFLVPEMDSEDPREYFRRHKDDLSHHASTHIGVKNKRILTNQLPLEVIPNAFALHGLALNWSRKDDVASILGNELFKADEYIRKGIVPPSPAFLEVQPTDISLWTTRLSTLTQKQYRSEHVGGEVGTKLVFDTVPMSHASAP” |
| *Arthroderma benhamiae* CBS 112371 XP_003015580.1 (ARB_05891, Mlh2) +XP_003015579.1 (ARB_05890) | | |
|  | old | XP_003015580.1 /coded_by=”complement(NW_003315110.1:195945..196487)”  XP_003015579.1 /coded_by=”complement(NW_003315110.1:193918..195290,NW_003315110.1:195338..195566)” |
|  | new | /coded_by=”complement(join(NW_003315110.1:193918..195290,NW_003315110.1:195338..195735,NW_003315110.1:195949..196355))” |
|  | old | XP_003015580.1 /sequence=”MATPDLNSSTSSISPSCDDILISLRSQLNLSPFSIDYDIQFKGNMSIVALEDPAVRAIGSTSALPDSSSVVKELLDNALDAGATSIFIEISLNTLDIIQVKDNGSGILPSDRSLACKQNYTSKIQTKEDLKNVGGRSLGFRGQALASIAEMSDAMYITTRVPEEQVARTVKFGRDGEPIR”  XP_003015579.1 /sequence=”MRVLKSKEKDWVYAPSQKPSVPDAILQVIGSTVISSCISSNASYPIPGEKDSPDSMVENYDTPTIHMSIIVPNPEAECMARINHKGQFVIVDARPLLTCMGFGKEVFKLFKSYYKKAVNSNILEDPFMFLSLDCPPGIYDANIEPSKDDILFEDHQAVLQIIEGVFMDIYNLETSTIIPTPDTQQSYLSNQLTIDISSPASTKNRGRFRSNEKLTSTVNPWTLSLAAQRLRDPESQLLTPQREFQDPRNNIPPRSPNIRVKNSRISMRQATLSLHDNGRVSLFNTQSPQRTSNGSPRMVTPPRVPAPNIPRSIEASVQIAEGNSARGKEGMYVIRPTPQTPPPSRAFSAATHRQAPIAVTFSRSNDQIPPNPHSISGRLHCNHRSDHKISGYGEIRPYKLTVPSGSHLQTASTNLKSRFQPDLNQKRNHLTSYRKNNFSTEIILYQSQSTMECMYETTIPNGGEITQLADSIKRLVNTDQYVKSGSLMAAFSPADLTSSISYWTNKLHTLAKNPSFQRQLRQLSFLTPSNSSK” |
|  | new | /sequence=”MSIVALEDPAVRAIGSTSALPDSSSVVKELLDNALDAGATSIFIEISLNTLDIIQVKDNGSGILPSDRSLACKQNYTSKIQTKEDLKNVGGRSLGFRGQALASIAEMSDAMYITTRVPEEQVARTVKFGRDGEPISDTPASHPIGTTVRVCDFLKSLPVRRQEAEKKSTKSILAIKKLLRGYAIARPKTRLSMRVLKSKEKDWVYAPSQKPSVPDAILQVIGSTVISSCISSNASYPIPGEKDSPDSMVENYDTPTIHMSIIVPNPEAECMARINHKGQFVIVDARPLLTCMGFGKEVFKLFKSYYKKAVNSNILEDPFMFLSLDCPPGIYDANIEPSKDDILFEDHQAVLQIIEGVFMDIYNLETSTIIPTPDTQQSYLSNQLTIDISSPASTKNRGRFRSNEKLTSTVNPWTLSLAAQRLRDPESQLLTPQREFQDPRNNIPPRSPNIRVKNSRISMRQATLSLHDNGRVSLFNTQSPQRTSNGSPRMVTPPRVPAPNIPRSIEASVQIAEGNSARGKEGMYVIRPTPQTPPPSRAFSAATHRQAPIAVTFSRSNDQIPPNPHSISGRLHCNHRSDHKISGYGEIRPYKLTVPSGSHLQTASTNLKSRFQPDLNQKRNHLTSYRKNNFSTEIILYQSQSTMECMYETTIPNGGEITQLADSIKRLVNTDQYVKSGSLMAAFSPADLTSSISYWTNKLHTLAKNPSFQRQLRQLSFLTPSNSSK” |
| *Arthroderma otae* (*Microsporum canis*) CBS 113480 XP_002846118.1 (Mlh2, MCYG_05987) | | |
|  | old | /coded_by=”XM_002846072.1:1..2259”  /coded_by=”join(NW_003299165.1:2434782..2435188,NW_003299165.1:2435521..2435599,NW_003299165.1:2435644..2436058,NW_003299165.1:2435644..2436058)” |
|  | new | /coded_by=”join(NW_003299165.1:2434782..2435188,NW_003299165.1:2435661..2436058,NW_003299165.1:2436106..2437463)” |
|  | old | /sequence=”MPIVALGESTIRAIGSASALPDPSSVAKELLDNALDAGATSIFVEISVNTLDIIQVKDNGSGIQPADRSLVCKQNYTSKIQTKEDLNTINGKSLGFRGQALASIAKMSDAVYITTRVIEEQAARIIKFGRDGKPIRGIPVSGNVHELLSSQMWKYELPVGQRISYNISDTLASHPVGTTVRVIGFLKSLPGRRQEAEKKSAKSAFNIKKLLQRYAIARPKTRFSFRLLKSKDNDWVYAPSSEASVRDAISQVISPTVMTSCISRNLSYPIHEERNSPYTEEEVHDVCTIHISTIIPNPSAECLSNVDYKGQYVVIDGRPILTSKGFGKEISRRFKTRLKQAIDESNVIADPFMFLSLDCPPGIYDINIEPSKDDVLFEDHPVILQIIETAFMDVYCLGASTNSHLMDTHRNIPNKNDEQASLSTGVSSPALFKGSGCLPTVIASTPTANPWKLSLSARQLRDPGPHLLTPGREPQLSTNSLPLDSPCYRITHNRNPMKQTTLRLDGYNKSLTLTKQKPEAVSNGSFPRAIPTQEYRINVPHTIGSFLHAPVRNIIRGNEEIHLLRPYPHTAPCSKISSVAKSPQYFKFSVSNSFEEVTLSVGHSILRHAPHNHRIDYGSPGYIQVPPTYKSVVPADISLRISSPDLTSDSQRPSTNGYNNSDGRPVYGGENGMNNISEQGSQSPMECTPSVDSVYEETTHCRHGIQEFSDSIKLMVDTDHYVRSGTIEATFSQADSTSNLSYWTSRLRVLAK” |
|  | new | /sequence=”MPIVALGESTIRAIGSASALPDPSSVAKELLDNALDAGATSIFVEISVNTLDIIQVKDNGSGIQPADRSLVCKQNYTSKIQTKEDLNTINGKSLGFRGQALASIAKMSDAVYITTRVIEEQAARIIKFGRDGKPIRDTLASHPVGTTVRVIGFLKSLPGRRQEAEKKSAKSAFNIKKLLQRYAIARPKTRFSFRLLKSKDNDWVYAPSSEASVRDAISQVISPTVMTSCISRNLSYPIHEERNSPYTEEEVHDVCTIHISTIIPNPSAECLSNVDYKGQYVVIDGRPILTSKGFGKEISRRFKTRLKQAIDESNVIADPFMFLSLDCPPGIYDINIEPSKDDVLFEDHPVILQIIETAFMDVYCLGASTNSHLMDTHRNIPNKNDEQASLSTGVSSPALFKGSGCLPTVIASTPTANPWKLSLSARQLRDPGPHLLTPGREPQLSTNSLPLDSPCYRITHNRNPMKQTTLRLDGYNKSLTLTKQKPEAVSNGSFPRAIPTQEYRINVPHTIGSFLHAPVRNIIRGNEEIHLLRPYPHTAPCSKISSVAKSPQYFKFSVSNSFEEVTLSVGHSILRHAPHNHRIDYGSPGYIQVPPTYKSVVPADISLRISSPDLTSDSQRPSTNGYNNSDGRPVYGGENGMNNISEQGSQSPMECTPSVDSVYEETTHCRHGIQEFSDSIKLMVDTDHYVRSGTIEATFSQADSTSNLSYWTSRLRVLAK” |
| *Aspergillus terreus* NIH2624 XP_001216390.1 (Mlh2, ATEG_07769) | | |
|  | old | /coded_by=”complement(join(NT_165934.1:958497..958717,NT_165934.1:958854..959846,NT_165934.1:959954..960342,NT_165934.1:960393..960799))” |
|  | new | /coded_by=”complement(join(NT_165934.1:958123..959906,NT_165934.1:959954..960342,NT_165934.1:960393..960799))” |
|  | old | /sequence=”MPITALPQDTARAIGSTSVISDPCSVVKELLDNALDASATSVFIEISQNTVDVIQVKDNGCGIPPSDHALVCKRAHTSKIATVEDLKKIGGSSLGFRGEALASTAEVSGGVTVTTRVDTEPVASVIRYGRDGGVISVQRASHPVGTTVRVTDLFKHIPVRRQTTLKNTTKTLARVKRLIQEYAAAQPSKRLSLKVLKAKNENGNWMYAPKPNAGLLDAALKIAGTDVASTCVLKHWPIPEATSDFTCVEGNPDCRMTALVLDPNAASRGIGKNIAKIYKSYVRSGSAARESTTTVTDPFLCLHVYCNEATYDVNIEPAKDDVLFENTPSILEAIEELCRDVYGAKEGDSEKRSTSVKGKEPVRQRDGFELLLASSQSTSQGQQRSVNADEAGNRHPTPVTQTTPQPSRGRVQGLSDERRRDSDIRAYSANRESLNPWTITKIITPVNRPGPASTDTSARRLSVDRTWPSPGQHRNSESASRRSSVASCLPSPSASDSSPTSNSPPGLSSSGTPFPISQTSPSLRTNYAKRAARERDRERYGNGSLDTWFGKTTQVGLSRIAAEDPSSPEEQESLTQLAQERFGSETQPSPSQSRPAGCTSFPNRLPHPAWQKLLILRTASGKLSRRDGNKLGDVKNNPPLLVLLILTDILPRGLHYLRIDLREDNQEPP” |
|  | new | /sequence=”MPITALPQDTARAIGSTSVISDPCSVVKELLDNALDASATSVFIEISQNTVDVIQVKDNGCGIPPSDHALVCKRAHTSKIATVEDLKKIGGSSLGFRGEALASTAEVSGGVTVTTRVDTEPVASVIRYGRDGGVISVQRASHPVGTTVRVTDLFKHIPVRRQTTLKNTTKTLARVKRLIQEYAAAQPSKRLSLKVLKAKNENGNWMYAPKPNAGLLDAALKIAGTDVASTCVLKHWPIPEATSDFTCVEGNPDCRMTALVLDPNADLAKASNLGQYISIDGRPISASRGIGKNIAKIYKSYVRSGSAARESTTTVTDPFLCLHVYCNEATYDVNIEPAKDDVLFENTPSILEAIEELCRDVYGAKEGDSEKRSTSVKGKEPVRQRDGFELLLASSQSTSQGQQRSVNADEAGNRHPTPVTQTTPQPSRGRVQGLSDERRRDSDIRAYSANRESLNPWTITKIITPVNRPGPASTDTSARRLSVDRTWPSPGQHRNSESASRRSSVASCLPSPSASDSSPTSNSPPGLSSSGTPFPISQTSPSLRTNYAKRAARERDRERYGNGSLDTWFGKTTQVGLSRIAAEDPSSPEEQESLTQLAQERFGSETQPSPSQSRPAGRSHVEDSSSRTPNSPLSTRSQHSPSEVPTARPEPGWKRLDQWSSRLHELSQPTATPGLAEALDFENRKREAIQARREQIRRRQEQSTPASSPHLNRYLAARAALSEDRPEGGQSGTAMNPHDPRAYLMRHRAYQENNMTGDERPRRVNTNKLPFENIPDGGLHKTGLVLTQDLSTLAASFSQTSRVDMYTESGCQYEGLTAETEPDIINLWSSRLSGLIKKQFRDEQGSMAHNLQFDFSSIQ” |
| *Bipolaris sorokiniana* ND90Pr (*Cohliobolus sativus* ND90Pr) EMD66996.1 (Mlh2, COCSADRAFT_351575) | | |
|  | old | /coded_by=”complement(join(KB445639.1:333100..333278,KB445639.1:333870..333992,KB445639.1:334041..334824, KB445639.1:334891..335377,KB445639.1:335436..335872))” |
|  | new | /coded_by=”complement(join(KB445639.1:333104..333278,KB445639.1:333870..333992,KB445639.1:334041..335377,KB445639.1:335436..335872))” |
|  | old | /sequence=”MADASNTVTQPGIAALPPPTIRQLGSGQVIVDPSSVVKELIDNALDARAKSIFVDISANTIDSIQVKDDGHGIPSEDRSLVCRRYCTSKIRDLKDLRELGGRWLGFRGEALASMAEMSSAFNVTTRVEGEPVAVKLKYNRDGELESVTHDSHPVGATVKVTGFFDYIPVRKQTVVKNAAKCLAKIRRLMQAYALARPSIRFRLRVLKAKNSNSDFSYAPRAHADVGDAILKIFGRDCAAQCDFTTAEADGFEIRAFLPKPTAVGSDISGRSSFVAVDARLVSSNHGTIKQMVHACKQRLWKANPSLGGPSKDDVMFEDSDIVLGVWEKLLGSFYTEAVTNLDNDEPSSSSQQSYQLEPGNAVDQNRDETTSELHNTSGQNEASVSAPQPGWRHSMYGIDEEDLEFLQDDRTPVIDEEEGRHTAEVSNPWTIARMNAAIKPRGAASNRQLLSLAKSTRNVDSSSSASKIPATPNRVVSVEPLTPQTSSKANTVQSLLDDELERSIQRLPPPSSVVGSISDWVMDRRGGQSIDSTGTYFSGREVPGFDLRSETQAPEAWTSQLSQHRNIAAGPHPPKKTARRKEPKDATAPLFSTDSLQRHPAGEPGQEESARQIVEWVCRIDSKLYGMYERVSDADEISIVIEGGVKRALEGIEWEGEEGGEEEEMLMEL” |
|  | new | /sequence=”MADASNTVTQPGIAALPPPTIRQLGSGQVIVDPSSVVKELIDNALDARAKSIFVDISANTIDSIQVKDDGHGIPSEDRSLVCRRYCTSKIRDLKDLRELGGRWLGFRGEALASMAEMSSAFNVTTRVEGEPVAVKLKYNRDGELESVTHDSHPVGATVKVTGFFDYIPVRKQTVVKNAAKCLAKIRRLMQAYALARPSIRFRLRVLKAKNSNSDFSYAPRAHADVGDAILKIFGRDCAAQCDFTTAEADGFEIRAFLPKPTAVGSDISGRSSFVAVDARLVSSNHGTIKQMVHACKQRLWKANPSLGGVRNPFFCINIICPPGSYDPNIEPSKDDVMFEDSDIVLGVWEKLLGSFYTEAVTNLDNDEPSSSSQQSYQLEPGNAVDQNRDETTSELHNTSGQNEASVSAPQPGWRHSMYGIDEEDLEFLQDDRTPVIDEEEGRHTAEVSNPWTIARMNAAIKPRGAASNRQLLSLAKSTRNVDSSSSASKIPATPNRVVSVEPLTPQTSSKANTVQSLLDDELERSIQRLPPPSSVVGSISDWVMDRRGGQSIDSTGTYFSGREVPGFDLRSETQAPEAWTSQLSQHRNIAAGPHPPKKTARRKEPKDATAPLFSTDSLQRHPAGEPGQEESARQIVEWVCRIDSKLYGMYERVSDADEISIVIEGGVKRALEGIEWEGEEGGEEEEMLMEL” |
| *Botryotinia fuckeliana* B05.10 XP_001550968.1 (Mlh2, BC1G_10527) | | |
|  | old | /coded_by=”complement(NW_001814500.1:70266..72437,NW_001814500.1:72588..73025)” |
|  | new | /coded_by=”complement(NW_001814500.1:70266..73025)” |
|  | old | /sequence=”MAIKPLSDATIHLLGSSQVLTTPTSLIKELIDNALDAKATSIDILISQNTIDKIEVRDNGHGIQPDDLDALGRRGHTSKLTTFTELRNLGGNSLGFRGEALASACQLGDVSIITKMDGQAVATCVKLKALGGIQSQSRSSHPTGTTGTKGAWSYSPRPNISIKDSVSLAVGREAALQCSEASMIFPEPPPDRENDTPEGAEIPTGASPANDVATSHFRLEAFLPRPDAEQGKIGHGQYFSVDSRPVASDKGTMKKITIIFKRHLRSRLASGASPEKLKNPFMRLNIVCPKASYDPNVEPAKEDIIFENETLVLEAVEKMFKNFYGDCGTVMSKVARNNTVTQPTGFDILLARTPQQDSPSPSTVVNASLTPKKTPPASTPNSNSKTMSEVCPNIAGDTLDIAESDNYEISDKATTLGGKRKWGCDMSHDYTEYTDDFNTRNSVNKSQKQLDLNSNRLSVSTELNPWVIAKMTASSNTNSSSAISLDSTETDSVRSMSPRAVTKVISPVQASHLSDVSASITDVLQSHSNFKSHMHCNPLTAADLSDSTTEGGLPVVQPWHDHLEGNELTTATNFVRNSLMSPPSTSPNVQTQKQTRDRGPNKPFVPPSRTPQSSSVSSGHVQTRLGASYQPFRIQREACHQPNATANPVQEVQTQQPNAELEWAMDFEHRKESATRRRRDELQAMREEFQQPPATIQASSRPSPHKNRYNAALASLEMSQESQRENSSSANSEGVCTTLPDSDPRGYLMKQQKSFSVLSSITGGPRKLKRAQTLRLPLETIPADLQIHRLLHTAESDMDEISKLVMETSKQDTYVRRGSQARGLEMTATDRDTVGRELRHVVEAWMKSERGEKCDVEFVFGGLESLSGA” |
|  | new | /sequence=”MAIKPLSDATIHLLGSSQVLTTPTSLIKELIDNALDAKATSIDILISQNTIDKIEVRDNGHGIQPDDLDALGRRGHTSKLTTFTELRNLGGNSLGFRGEALASACQLGDVSIITKMDGQAVATCVKLKALGGIQSQSRSSHPTGTTVCVMNFMSRLPVRKQTALKEAPKTIGKIQELIRRYALARLSVKLTLKIVKGTKGAWSYSPRPNISIKDSVSLAVGREAALQCSEASMIFPEPPPDRENDTPEGAEIPTGASPANDVATSHFRLEAFLPRPDAEQGKIGHGQYFSVDSRPVASDKGTMKKITIIFKRHLRSRLASGASPEKLKNPFMRLNIVCPKASYDPNVEPAKEDIIFENETLVLEAVEKMFKNFYGDCGTVMSKVARNNTVTQPTGFDILLARTPQQDSPSPSTVVNASLTPKKTPPASTPNSNSKTMSEVCPNIAGDTLDIAESDNYEISDKATTLGGKRKWGCDMSHDYTEYTDDFNTRNSVNKSQKQLDLNSNRLSVSTELNPWVIAKMTASSNTNSSSAISLDSTETDSVRSMSPRAVTKVISPVQASHLSDVSASITDVLQSHSNFKSHMHCNPLTAADLSDSTTEGGLPVVQPWHDHLEGNELTTATNFVRNSLMSPPSTSPNVQTQKQTRDRGPNKPFVPPSRTPQSSSVSSGHVQTRLGASYQPFRIQREACHQPNATANPVQEVQTQQPNAELEWAMDFEHRKESATRRRRDELQAMREEFQQPPATIQASSRPSPHKNRYNAALASLEMSQESQRENSSSANSEGVCTTLPDSDPRGYLMKQQKSFSVLSSITGGPRKLKRAQTLRLPLETIPADLQIHRLLHTAESDMDEISKLVMETSKQDTYVRRGSQARGLEMTATDRDTVGRELRHVVEAWMKSERGEKCDVEFVFGGLESLSGA” |
| *Botryotinia fuckeliana* B05.10 XP_001558189.1 (Mlh1, BC1G_03221), gene annotation complicated by a gap in the genome assembly | | |
|  | old | /coded_by=”join(NW_001814560.1:432880..432984,NW_001814560.1:4333039..433218,NW_001814560.1:433829..433900,NW_001814560.1:434124..435395) |
|  | new | /coded_by=”join(NW_001814560.1:432880..432984,NW_001814560.1:4433039..433218,NW_001814560.1:433273..433338,NW_001814560.1:433817..433900,NW_001814560.1:433902..435395)” |
|  | old | /sequence=”MSEPMDMDAGTETSRGTKRKAESELPEVTAPGRIKALDPDVVNKIAAGEIIVAPVHALKELIENAVDAGSTSLEVLVKDGGLKLLQITDNGHGINGVAFSCKKHGEASTTISTQLASSTGGHPFTYLNLEIEPHRLDVNVHPTKREVNFLNEEEIIEKICNDIRIKLADVDKSRNFMTQTLLPGAQVPLVADTLDPVAFAAADRARTTTRPYENNLVRTDAKLRKITTMLPPTTKPTTTANREATPNPSGSTVAPNSQDIEYTHSDREPIICRLMTIKELRTSVRDSMHNELTEIFASHTFVGIVDERRRLAAIQSGVKLFLVDYAAISSAFFYQVGLTDFGNFGEIRFNPPLSLTSLLTLAATHEKATQPPNTSPEDDFEVEDVVEIVSEQLISRREMLQEYFSLSITPDGLVEGIPLLLKNYTPALSKLPQFLLRLGPHVNWNDEKECFSSFLQELARFYVPEQLPPSPGPEHQDVNEPETQTTSISPELKQRRDAVRKMVEDVLFPAFRSRLLATRDLMGGAVLEVANLKGLYRVFERC” |
|  | new | /sequence=”MSEPMDMDAGTETSRGTKRKAESELPEVTAPGRIKALDPDVVNKIAAGEIIVAPVHALKELIENAVDAGSTSLEVLVKDGGLKLLQITDNGHGINERDMAILCERFTTSKLKQFEDLIHCKGVAFSCKKHGEASTTISTQLASSTVDRIRQIHGSGVANELIEFKSADPQWGFTAQGWTTNANYHVKKTTLLLFINHRAVESTAIRKAIEQTYSAFLPKGGHPFTYLNLEIEPHRLDVNVHPTKREVNFLNEEEIIEKICNDIRIKLADVDKSRNFMTQTLLPGAQVPLVADTLDPVAFAAADRARTTTRPYENNLVRTDAKLRKITTMLPPTTKPTTTANREATPNPSGSTVAPNSQDIEYTHSDREPIICRLMTIKELRTSVRDSMHNELTEIFASHTFVGIVDERRRLAAIQSGVKLFLVDYAAISSAFFYQVGLTDFGNFGEIRFNPPLSLTSLLTLAATHEKATQPPNTSPEDDFEVEDVVEIVSEQLISRREMLQEYFSLSITPDGLVEGIPLLLKNYTPALSKLPQFLLRLGPHVNWNDEKECFSSFLQELARFYVPEQLPPSPGPEHQDVNEPETQTTSISPELKQRRDAVRKMVEDVLFPAFRSRLLATRDLMGGAVLEVANLKGLYRVFERC” |
| *Capitella teleta* ELU13965.1 (Mlh1, CAPTEDRAFT_199117) + ELU13964.1 (Mlh1, CAPTEDRAFT_223290) | | |
|  | old | ELU13964.1 /coded_by=”complement(join(KB294881.1:170450..170605,KB294881.1:172263..172466,KB294881.1:172524..172685,KB294881.1:173722..173785,KB294881.1:173838..173946,KB294881.1:174002..174359,KB294881.1:174664..174696,KB294881.1:175645..175798,KB294881.1:175858..175951,KB294881.1:176002..176068))”  ELU13965.1 /coded_by=”complement(join(KB294881.1:<176253..176256,KB294881.1:176498..176589,KB294881.1:176644..176716,KB294881.1:176767..176939,KB294881.1:178947..179219))” |
|  | new | /coded_by=”complement(join(KB294881.1:170450..170605,KB294881.1:172263..172466,KB294881.1:172524..172685,KB294881.1:173722..173785,KB294881.1:173838..173946,KB294881.1:174002..174359,KB294881.1:174664..174696,KB294881.1:175645..175798,KB294881.1:175858..175951,KB294881.1:176002..176114,KB294881.1:176168..176256,KB249881.1:176316..176358,KB249881.1:176498..176589,KB249881.1:176644..176716,KB249881.1:176767..176939,KB249881.1:178947..179219))” |
|  | old | ELU13965.1  /sequence=”MEYIRLFPAPVSRIARQWGTEISTWVQPVNHLEHLGVPSLRHSSSDLSLSLLRGKKIVTIPPTVVFFAPFQAIPPTIGFHVMFRYSPDDRMKEDMSIVCERFTTSKLKTFEDLTQIATYGFRGEALASISHVAHVTITTRTNESKCAYRGAFSDGQLKAPLKPCAGNVGTQILVEDLFYNVATRRKALRSPGEEHSKVVDVVSSM”  ELU13964.1  /sequence=”MNGWVSNANYSIKKCTMLLFINHRLVDSTALRKAIEAVYAVYLPKNMHPFLYISLEIAPQNIDVNVHPTKHEVHFLHEDSVIESVQKCIDARLLGSNASRTYFTQVYKGFLWNACLVNKKLMTINWSEQTAEIRNWMPSSRRRPAKLAQSLTGLLTPRSLHLNHQSMATNSLHHNFLFFIFLTREVEKLEGKQPEAAAQSNPATPSMQETESNRRPVKLSSVLKLQEDIRSNCHEGLRDMLTNHTFVGCVDPDLSLMQHQTKLYLTNTARLSKELFYQLLLFDFGNFGVLRLSEPAPLNELAMMALESSASGWTEADGPKEDLASYIVQFLTSKAEMLDDYFSIEIKDNALCTLPMLLDNYVPALEGLPMYVLRLSTEVNWDSERECFDTFARETSEFYSMKSKWISPAQDSGSAEDGANSWRWSVEHTLFPAFRSLLLPPSEMAADGSILQLANLPDLYKVFERC” |
|  | new | /sequence=”MEYIRLFPAPVSRIARQWGTEISTWVQPVNHLEHLGVPSLRHSSSDLSLSLLRGKKIVTIPPTVVFFAPFQAIPPTIGFHVMFRYSPDDRMKEDMSIVCERFTTSKLKTFEDLTQIATYGFRGEALASISHVAHVTITTRTNESKCAYRGAFSDGQLKAPLKPCAGNVGTQILVEDLFYNVATRRKALRSPGEEHSKVVDVVSRYAVHNSKVAFTLKKHGESLAEVRTPANSTHVDNIRSIYGPAVAKELLEVTHEDSQLGFTMNGWVSNANYSIKKCTMLLFINHRLVDSTALRKAIEAVYAVYLPKNMHPFLYISLEIAPQNIDVNVHPTKHEVHFLHEDSVIESVQKCIDARLLGSNASRTYFTQVYKGFLWNACLVNKKLMTINWSEQTAEIRNWMPSSRRRPAKLAQSLTGLLTPRSLHLNHQSMATNSLHHNFLFFIFLTREVEKLEGKQPEAAAQSNPATPSMQETESNRRPVKLSSVLKLQEDIRSNCHEGLRDMLTNHTFVGCVDPDLSLMQHQTKLYLTNTARLSKELFYQLLLFDFGNFGVLRLSEPAPLNELAMMALESSASGWTEADGPKEDLASYIVQFLTSKAEMLDDYFSIEIKDNALCTLPMLLDNYVPALEGLPMYVLRLSTEVNWDSERECFDTFARETSEFYSMKSKWISPAQDSGSAEDGANSWRWSVEHTLFPAFRSLLLPPSEMAADGSILQLANLPDLYKVFERC” |
| *Coccidioides immitis* RS XP_001241612.1 (Mlh2, CIMG_08775) Frameshift in genome sequence | | |
|  | old | /coded_by=”join(NW_001509358.1:3903541..3903947,NW_001509358.1:3903998..3904383,NW_001509358.1:3904507..3904720,NW_001509358.1:3905048..3906090)” |
|  | new | /coded_by=”join(NW_001509358.1:3903541..3903947,NW_001509358.1:3903998..3904383,NW_001509358.1:3904438..3904841,NW_001509358.1:3904840..3906090)” |
|  | old | /sequence=”MPIAELDRAAAHAIGSASAISDPCSVVRELIDNALDANATIISVELSANLLDTIHVKDNGLGIPPEDRELICRQNCTSKIKTLDDLKHVGGKLLGFRGQALASTTQMSESTVIITRITAETTASILEFGRAGDILSSKGMVAHPVGTSVRVSGFLKYLPVRRQMVLKSSSKIVSRIKKALQNYALSRPWVRLSLKISRQKRGFAWIYAPKQSPSVADAISKIIGAEVAAHRLGQSLTHNDPNPENSDEGKSTAQVHAVIPKLDAGIAKDLVKIYKSYINRTFGRPSTPAITDPFVYLYLSCPPGIYDVNVDPSKDDVLFADSRSILCLAEDLFGKIPQSLIDNVSVPQNGRRSANRTPHLAISPGAIDNVEVSPIQTRTLKIKSYRKRMHRGSRKPEGECGVSIHKHAINRAQSDFGTSPDEAPNSVATSQHLENEALQIALSERFGKRTPGLNIGSLVSPAVTERGGTIPFPVKTNIQNGTLPGTLRPTHSHPEFLYSECHKKLAVHPQCMVEQEMHQSKSKIDSLNAPYPTGSATFIMSPKAQSVVAKDSAMLIRKATKLPFNTPQAHSQTIQYQNQRQIRDERMPGQEETLKVSTIHHLATSQVGTTAEVELLNRQLCGIDSYIQTGNISPALVEPCPASTIEQWRATILDFITGQFRSESTDVCTRLYLDSKSLFPTV” |
|  | new | /sequence=”MPIAELDRAAAHAIGSASAISDPCSVVRELIDNALDANATIISVELSANLLDTIHVKDNGLGIPPEDRELICRQNCTSKIKTLDDLKHVGGKLLGFRGQALASTTQMSESTVIITRITAETTASILEFGRAGDILSSKGMVAHPVGTSVRVSGFLKYLPVRRQMVLKSSSKIVSRIKKALQNYALSRPWVRLSLKISRQKRGFAWIYAPKQSPSVADAISKIIGAEVAAHRLGQSLTHNDPNPENSDEGKSTAQVHAVIPKLDADISKLENKGQFIFVDGRPLSTSKGIAKDLVKIYKSYINRTFGRPSTPAITDPFVYLYLSCPPGIYDVNVDPSKDDVLFADSRSILCLAEDLFGKMYGNEQLGDERQSETYHGSHQQEEMFRGTSDTEGNLFKRPAPTMIFPLPEQRRLRRPSPGKLHAHSKQKRECAHKEKHATDVALNPWTLARRTSTQRHRDPNSHLLTPSRSPQSLIDNVSVPQNGRRSANRTPHLAISPGAIDNVEVSPIQTRTLKIKSYRKRMHRGSRKPEGECGVSIHKHAINRAQSDFGTSPDEAPNSVATSQHLENEALQIALSERFGKRTPGLNIGSLVSPAVTERGGTIPFPVKTNIQNGTLPGTLRPTHSHPEFLYSECHKKLAVHPQCMVEQEMHQSKSKIDSLNAPYPTGSATFIMSPKAQSVVAKDSAMLIRKATKLPFNTPQAHSQTIQYQNQRQIRDERMPGQEETLKVSTIHHLATSQVGTTAEVELLNRQLCGIDSYIQTGNISPALVEPCPASTIEQWRATILDFITGQFRSESTDVCTRLYLDSKSLFPTV” |
| *Coccidioides posadasii* C735 delta SOWgp EER28443.1 (Mlh2, CPC735_063160) | | |
|  | old | /coded_by=”join(ACFW01000015.1:866886..867292,ACFW01000015.1:867346..867728,ACFW01000015.1:867807..869437)” |
|  | new | /coded_by=”join(NW_003316013.1:866886..867292,NW_003316013.1:867346..867728,NW_003316013.1:867783..869437)” |
|  | old | /sequence=”MPIAELDRAAAHAIGSASAISDPCSVVRELIDNALDANATIISVELSANLLDSIHVKDNGLGIPPEDRELICRQNCTSKIKTLDDLKHVGGKLLGFRGQALASTTQMSESTVIITRITAETTASILEFGRAGDILSKGMVAHPVGTSVRVSGFLKYLPVRRQMVLKSLSKIVSRIKKALQNYALSRPCVRLSLKISRQKRGFAWIYAPKQSPSVADAISKVIGAEVAAHCLEQSLTYNDPNPENSDEGKSTAQVHAVIPKLDAGQFIFVDGRPLSTSKGIAKDLVKIYKSYINRTFGRPCTSAITDPFMYLYLSCPPGIYDVNVDPSKDDVLFADSRSILCLAEDLFGKMYDNEQLGDERQSETYRGSHQQEEMFRGTSDTEGNLFKRPEPTMIFPLPEQRRLRRPSPGKLHAHSRQKRECAHKEKHATDVALNPWTLARRTSTQRHRDPNSHLLTPSRSPQSLIDNVSVPQNGRRSANRTPHLAISPGAIDNVEVSPIQTKTLKIKSHRKRMHRGSRKPEGECGVSIYKHAINRAQSDFGTSPDKTPNSVATSQHLENEALQIALSERFGKWIPGLNIGSLVSPAVTERGGTLPFPVKTNIQNGTLPGTLRPTHSHPEFLYSECHKKLAVHPQCMVEQEMHQPKSKIDSLNAPYPTRSATFIMSPKAQSVVAKDSAMLIRKATKLPFNTPQAHSQTIQYQNQREIRDERMPGQEETLKVSTIHHLATSQVDTTAEVELLNRQLCGIDSYIQTGNISPALVEPCPASTIEQWRATILDFITGQFRSESTDVCTRLYLDSNSLFPTV” |
|  | new | /sequence=”MPIAELDRAAAHAIGSASAISDPCSVVRELIDNALDANATIISVELSANLLDSIHVKDNGLGIPPEDRELICRQNCTSKIKTLDDLKHVGGKLLGFRGQALASTTQMSESTVIITRITAETTASILEFGRAGDILSKGMVAHPVGTSVRVSGFLKYLPVRRQMVLKSLSKIVSRIKKALQNYALSRPCVRLSLKISRQKRGFAWIYAPKQSPSVADAISKVIGAEVAAHCLEQSLTYNDPNPENSDEGKSTAQVHAVIPKLDADISKLENKGQFIFVDGRPLSTSKGIAKDLVKIYKSYINRTFGRPCTSAITDPFMYLYLSCPPGIYDVNVDPSKDDVLFADSRSILCLAEDLFGKMYDNEQLGDERQSETYRGSHQQEEMFRGTSDTEGNLFKRPEPTMIFPLPEQRRLRRPSPGKLHAHSRQKRECAHKEKHATDVALNPWTLARRTSTQRHRDPNSHLLTPSRSPQSLIDNVSVPQNGRRSANRTPHLAISPGAIDNVEVSPIQTKTLKIKSHRKRMHRGSRKPEGECGVSIYKHAINRAQSDFGTSPDKTPNSVATSQHLENEALQIALSERFGKWIPGLNIGSLVSPAVTERGGTLPFPVKTNIQNGTLPGTLRPTHSHPEFLYSECHKKLAVHPQCMVEQEMHQPKSKIDSLNAPYPTRSATFIMSPKAQSVVAKDSAMLIRKATKLPFNTPQAHSQTIQYQNQREIRDERMPGQEETLKVSTIHHLATSQVDTTAEVELLNRQLCGIDSYIQTGNISPALVEPCPASTIEQWRATILDFITGQFRSESTDVCTRLYLDSNSLFPTV” |
| *Colletotrichum gloeosporioides* Nara gc5 ELA31540.1 (Mlh2, CGGC5_8372) Stop codon in first exon | | |
|  | old | /coded_by=”complement(join(KB020744.1:115393..117284,KB020744.1:117348..117799,KB020744.1:117962..118503))” |
|  | new | /coded_by=”complement(join(KB020744.1:115393..117284,KB020744.1:117348..118503))” |
|  | old | /sequence=”MPITRLPLTTVRRLGSSVVIVTPVSLVKELVDNSIDAGATSIEITVSANAVDKIQVRDNGLGIDSEDFDALGRRSHTSKLRTFEELQFKGGQTLGFRGDALASTNIMSKLTITTRTAEDPVANLLTLNPNGGGILAQKPVAGPVGTTVDASDIFSGFPVRRQQAIKESKRAHNQIKELLFTGNTLEGGAKRSRPDTPASILRLEAFMPKPGAKPAAIAGKGYFISVDARPMTTARGTMKKLVGIYKQHLRKHVGASSENLKTPFIRLNIECILGSYDPNVARNKDEVLFANEPKLLQLFEDMCQDLYQNCDGQADIPKQNAVVESSSSRQGGHLYNLEATPAEKASHVINRQLASPPSDRTSAVESSAAFANKAGGYHDHPDRSALKYTTHLADDTLSLANEKTSQTNSSEAQISRQDSLESSSDSGLTQSFLRSSWDVDMARSNTASPVEDTREMFLNPLLSDISELLSTQRMEQSQWEKPNPWSLAKKAAKKSGGIHSDHSKNSADIEQSEDNTTVPVLKESTHIPNVENVVHETESMTKLVPEDRENALGQLSQRHQALAMQREHAHETDDFESSRLRTLDAPHGFPQDPSHIGVPRILGDEEQFQRPGLRHAFDDLPDDIARDLGLHRRRAQTPHPSTIRGQDNFFGTPPSSSSPLHKPFRVPARAEVGRQERSSARSNRPRGAQGQPSVGDRKRNGLSQSKLAFNATKSLKSSQPGEFLHAAAGCSPHGDFDDNYDAEPEITWHLRHNSPSPTERDIAGAIERLNALRPRSHSIIHKDLDRSVSLSPTERDISSAVSRMKVLRHQKDAFEPPIEDTTFKERGRPLIRGSTTTTLEISPRGYLRKRLASRSRSRNKTHKRMKSELLPFERPSEELYLQLKSLMIDLSQLRKQATKAFPWDLYVSRDIPESPAFDLEPAEMDDISNRLQEIVAEWAFDKYAVELDLEINLGDLLCGDA” |
|  | new | /sequence=”MPITRLPLTTVRRLGSSVVIVTPVSLVKELVDNSIDAGATSIEITVSANAVDKIQVRDNGLGIDSEDFDALGRRSHTSKLRTFEELQFKGGQTLGFRGDALASTNIMSKLTITTRTAEDPVANLLTLNPNGGGILAQKPVAGPVGTTVDASDIFSGFPVRRQQAIKESKRAHNQIKELLFTYALTIPRIRLSLKIQSNHKLSWSYASTAGADVREAVLQVFGNELVSQCV*KTYSGNTLEGGAKRSRPDTPASILRLEAFMPKPGAKPAAIAGKGYFISVDARPMTTARGTMKKLVGIYKQHLRKHVGASSENLKTPFIRLNIECILGSYDPNVARNKDEVLFANEPKLLQLFEDMCQDLYQNCDGQADIPKQNAVVESSSSRQGGHLYNLEATPAEKASHVINRQLASPPSDRTSAVESSAAFANKAGGYHDHPDRSALKYTTHLADDTLSLANEKTSQTNSSEAQISRQDSLESSSDSGLTQSFLRSSWDVDMARSNTASPVEDTREMFLNPLLSDISELLSTQRMEQSQWEKPNPWSLAKKAAKKSGGIHSDHSKNSADIEQSEDNTTVPVLKESTHIPNVENVVHETESMTKLVPEDRENALGQLSQRHQALAMQREHAHETDDFESSRLRTLDAPHGFPQDPSHIGVPRILGDEEQFQRPGLRHAFDDLPDDIARDLGLHRRRAQTPHPSTIRGQDNFFGTPPSSSSPLHKPFRVPARAEVGRQERSSARSNRPRGAQGQPSVGDRKRNGLSQSKLAFNATKSLKSSQPGEFLHAAAGCSPHGDFDDNYDAEPEITWHLRHNSPSPTERDIAGAIERLNALRPRSHSIIHKDLDRSVSLSPTERDISSAVSRMKVLRHQKDAFEPPIEDTTFKERGRPLIRGSTTTTLEISPRGYLRKRLASRSRSRNKTHKRMKSELLPFERPSEELYLQLKSLMIDLSQLRKQATKAFPWDLYVSRDIPESPAFDLEPAEMDDISNRLQEIVAEWAFDKYAVELDLEINLGDLLCGDA” |
| *Fusarium oxysporum* f. sp. cubense race 1 ENH60712.1 | | |
|  | old | /coded_by=”complement(join(KB731261.1:536404..537013,KB731261.1:537236..538815,KB731261.1:538921..539418))” |
|  | new | /coded_by=”complement(join(KB731261.1:536404..537013,KB731261.1:537236..539418))” |
|  | old | /sequence=”MSISVLPPSLAHLLRSSTSLPDPLSMVKELVDNSIDAGATSIEITVAPNTVDKVQVRDNGCGIQVDDYKSLGRRSHTSKLRNFDELHLKGGKTLGFRGEALASANYLATIKITTRTAQDPIASLLLLNTKSGGISRQQPVSAPVGTTVQALNLFQNLPVRKQNAIKPWVYTPCSAPSTREAITQVFDHALTTQLVAVSSDSRAGATSTEVQSKNLKIVASLPKPDSDANIIKGKGAFISVDSRPISSSKSTGKKLVSIFRSSISAVLNSPENSRAPSNAFMQLSIQCSPGSYDPNVTPSKDELLFVDEPAVLSCFQNLCDSVYTKKFLGDKELQHKLVALENSLSLLPWASKTVEPRRRDGARLDIMEKELLLTDQELVGSLNDKPERVLGGTGVFGSGERASPAAFTVPTSPKKSSRSGNLDETRGPKKAQPVQEMMRTRLTVNLSRRETASTDLDGKEGLIPVHVTPRRAATPPVRDELQSNSRRRGLTLNRRFGNIDDYFRSSRDEPIQIATDETATPEIPHTRSYLTSVSHNRRLPLKELSESLLNLFREYEEEDYEDESDDESSFNLPFAGPNAVPSPRTSPRREAPSFSSQPFRPLLGQQSMRNSMPDLQLTLPANLQTPPSSDPTVAHNSTRRDSRRQSTNNILARAQRSSRGSVVPSVNRRSGDELRQSRLLLGSGPTASQGRRRSFGGMRDSERSQPQGNKQWSHSLQVLLQRTPPPPQATGSDEEATGEYSPAPLDHIHDQHNEGFYSRSFKRQRRCSSVSTSESAELDSRRLLMKRQRIHANGGRLKRIASTKLPLEIISQNDSTVNLSVKVEVQMCELQAAALEFTNSGRATKPDLALQFRNMDEVEVVDRRLRRVTESWLKGNPSVTVEYILRSEVKGKSKA” |
|  | new | /sequence=”MSISVLPPSLAHLLRSSTSLPDPLSMVKELVDNSIDAGATSIEITVAPNTVDKVQVRDNGCGIQVDDYKSLGRRSHTSKLRNFDELHLKGGKTLGFRGEALASANYLATIKITTRTAQDPIASLLLLNTKSGGISRQQPVSAPVGTTVQALNLFQNLPVRKQNAIKVSRKTLADIRRLLERYAIALPHIRLSFKVPGSSIQPWVYTPCSAPSTREAITQVFDHALTTQLVAVSSDSRAGATSTEVQSKNLKIVASLPKPDSDANIIKGKGAFISVDSRPISSSKSTGKKLVSIFRSSISAVLNSPENSRAPSNAFMQLSIQCSPGSYDPNVTPSKDELLFVDEPAVLSCFQNLCDSVYTKKFLGDKELQHKLVALENSLSLLPWASKTVEPRRRDGARLDIMEKELLLTDQELVGSLNDKPERVLGGTGVFGSGERASPAAFTVPTSPKKSSRSGNLDETRGPKKAQPVQEMMRTRLTVNLSRRETASTDLDGKEGLIPVHVTPRRAATPPVRDELQSNSRRRGLTLNRRFGNIDDYFRSSRDEPIQIATDETATPEIPHTRSYLTSVSHNRRLPLKELSESLLNLFREYEEEDYEDESDDESSFNLPFAGPNAVPSPRTSPRREAPSFSSQPFRPLLGQQSMRNSMPDLQLTLPANLQTPPSSDPTVAHNSTRRDSRRQSTNNILARAQRSSRGSVVPSVNRRSGDELRQSRLLLGSGPTASQGRRRSFGGMRDSERSQPQGNKQWSHSLQVLLQRTPPPPQATGSDEEATGEYSPAPLDHIHDQHNEGFYSRSFKRQRRCSSVSTSESAELDSRRLLMKRQRIHANGGRLKRIASTKLPLEIISQNDSTVNLSVKVEVQMCELQAAALEFTNSGRATKPDLALQFRNMDEVEVVDRRLRRVTESWLKGNPSVTVEYILRSEVKGKSKA” |
| *Fusarium oxysporum* f. sp. cubense race 4 EMT62262.1 (Mlh2) | | |
|  | old | /coded_by=”join(KB726996.1:4116252..4116749,KB726996.1:4116855..4118434,KB726996.1:4118657..4119220,KB726996.1:4119350..4119393,KB726996.1:4119612..4119624,KB726996.1:4119819..4119834,KB726996.1:4119965..4120075)” |
|  | new | /coded_by=”join(KB726996.1:4116252..4118434,KB726996.1:4118657..4119220,KB726996.1:4119350..4119393,KB726996.1:4119612..4119624,KB726996.1:4119819..4119834,KB726996.1:4119965..4120075)” |
|  | old | /sequence=”MSISVLPPSLAHLLRSSSSLPDPLSMVKELVDNSIDAGATSIEITVAPNTVDKVQVRDNGCGIQVDDYKSLGRRSHTSKLRNFDELHLKGGKTLGFRGEALASANYLATIKITTRTAQDPIASLLLLNTKSGGISRQQPVSAPVGTTVQALNLFQNLPVRKQNAIKPWVYTPCSAPSTREAITQVFGHALTTQLVAVSSDSRAGATSTEVQSKNLKIVASLPKPDSDANIIKGKGAFISVDSRPISSSKSTGKKLVSIFRSSISAVLNSPENSRAPSNAFMQLSIQCSPGSYDPNVTPSKDELLFVDELAVLSCFQNLCDSVYTKKFLGDKELQHKLVALENSLSLLPCASKTVEPRRRDGARVDSMEKELLLTDQELVGSLNDKPERVLGGTDVFGSGERASPGAFTVPTSPKKSSRSGNLDETRGPKKAQPVQEMMRTRLTVNLSRRETASTDLDGKEGLIPVHVIPRRAATPPVRDELQSSPRRRGLTLNRRFGNIDDYFRSSRDEPIQIATDETATPEIPHTRSYLTSVSHNRRLPLKELSESLLNLFREYEEEDYEDESDDESSFNLPSAGPNAVPSPRTSPRREAPSFSSQPFRPLLGQQSIRNSMPDLQLTLPANLQTPPSSDPTGAHNSTRRDSRRQSTNNILVRAQRSSRGSVVPSVDRRSGDELRQSRLLLGSGPTASQGRRRSCGGMRDSERSQTQGNKQWSHSLQVLLQRTPPPQATGSDEEATGEYSPAPLHHIHDQHNEGFYSRSFKRQRRCSSVSTSESAELDSRRLLMKRQRIHANGGRLKRIASTKLPLEIISQNDSTVNLSVKVEVQMCELQAAALEFTNSGRATKPDLALQFRNMDEVEVVDRRLRRVTESWLKGNPSVTVEEIGEREERLLAEIAFDPETPQGKSDLGNIINGEQDISCSDGGWVVFGWATETVLLARGCS” |
|  | new | /sequence=”MSISVLPPSLAHLLRSSSSLPDPLSMVKELVDNSIDAGATSIEITVAPNTVDKVQVRDNGCGIQVDDYKSLGRRSHTSKLRNFDELHLKGGKTLGFRGEALASANYLATIKITTRTAQDPIASLLLLNTKSGGISRQQPVSAPVGTTVQALNLFQNLPVRKQNAIKVSRKTLADIRRLLERYAIALPHIRLSFKVPGSSIQPWVYTPCSAPSTREAITQVFGHALTTQLVAVSSDSRAGATSTEVQSKNLKIVASLPKPDSDANIIKGKGAFISVDSRPISSSKSTGKKLVSIFRSSISAVLNSPENSRAPSNAFMQLSIQCSPGSYDPNVTPSKDELLFVDELAVLSCFQNLCDSVYTKKFLGDKELQHKLVALENSLSLLPCASKTVEPRRRDGARVDSMEKELLLTDQELVGSLNDKPERVLGGTDVFGSGERASPGAFTVPTSPKKSSRSGNLDETRGPKKAQPVQEMMRTRLTVNLSRRETASTDLDGKEGLIPVHVIPRRAATPPVRDELQSSPRRRGLTLNRRFGNIDDYFRSSRDEPIQIATDETATPEIPHTRSYLTSVSHNRRLPLKELSESLLNLFREYEEEDYEDESDDESSFNLPSAGPNAVPSPRTSPRREAPSFSSQPFRPLLGQQSIRNSMPDLQLTLPANLQTPPSSDPTGAHNSTRRDSRRQSTNNILVRAQRSSRGSVVPSVDRRSGDELRQSRLLLGSGPTASQGRRRSCGGMRDSERSQTQGNKQWSHSLQVLLQRTPPPQATGSDEEATGEYSPAPLHHIHDQHNEGFYSRSFKRQRRCSSVSTSESAELDSRRLLMKRQRIHANGGRLKRIASTKLPLEIISQNDSTVNLSVKVEVQMCELQAAALEFTNSGRATKPDLALQFRNMDEVEVVDRRLRRVTESWLKGNPSVTVEEIGEREERLLAEIAFDPETPQGKSDLGNIINGEQDISCSDGGWVVFGWATETVLLARGCS” |
| *Glarea lozoyensis* 74030 EHL01996.1 (Mlh2, M7I_1946) | | |
|  | old | /coded_by=”complement(join(AGUE01000040.1:273820..276560,AGUE01000040.1:276837..276844,AGUE01000040.1:277197..277220,AGUE01000040.1:277296..277426,AGUE01000040.1:277448..277474))” |
|  | new | /coded_by=”complement(AGUE01000040.1:273820..276558)” |
|  | old | /sequence=”MQASQKNIKVPKLALKISGGPHVRYIDPAVSYGMSPIKNWQCFGELATNSEVFGSVEDRFVYLDMAIAALPQSTIHLLGSAQVLTSTTSLVKELIDNALDAKATSVEVLLSQNTLDKIQVRDNGHGIQHDDLDALGRRGHTSKLRSFEDLRSIGGVSLGFRGEALASAVQLGKVSVTTRTEGEAVATSVTLKAPGGIAKQSKTSHPIGTTVTILNFMSKLPVRKQTALKSASKTLVKTKELLQAYALARPSIRFSLKITKDSKGSWSFAPRPNDGVKEAVSHIIGKEASMQCLSKTLTFSEPRKTGDNEFHVEIYLPKPDADISKVGHGQYLSIDSRPVSHEKGTMKKIVSLFKSYLKVGLGEAREVRNPFIRININCPLSSYDPNVEPAKDDVIFGNESLILDPFESFFKDLYGEKESASNSTSKNKPAKPIDNFELLLARNPVVGVPSLNKGTARSDSNVQLKIIEFEDDSASAEKGVPDMTQDENSLFVPEAGETSPAIGKSRRSWGIDMSRDFDEVVTDPRSAVLNRKAKSSPAAIEDDSSNTLNPWVIAKMTAPLRTNAEPVAPTDQESRTPGAANDQYHIIDGNLIRRKSQEQLFLESPVQKHGSVSRRTPKRRYSNEARLPNPNTPKASLYELDRGTRHRSEDGNIYLEQRAKAFRDTTLETPDIPSLHRRQEHSSSEFGHELPNFNEQSVRRRQNDFVSARSIKESFLLTPPATQRQKPSHQSDKVNKPFAAPRRIADTTSTPTRLQQTRLPTFQGQQDFLGVEEPQSELEWAMQYEQNKESASRTRRDQLRLARKDTETETRTERTKSSPHKNRYNAAIAALEGDAVSKPQEPKTSLPPNDPRAYLMKHQALSSTADGEQTKAIRNTRAKSTKLPLEKIPDHLQTHKLVLNLSTDMEKIKEITEMFTPIDAYISQGRSEPGLELSVAATKDLSRDIKRMVDAWVEKQEDRRDCEVEYLFANLNSVEA” |
|  | new | /sequence=”MAIAALPQSTIHLLGSAQVLTSTTSLVKELIDNALDAKATSVEVLLSQNTLDKIQVRDNGHGIQHDDLDALGRRGHTSKLRSFEDLRSIGGVSLGFRGEALASAVQLGKVSVTTRTEGEAVATSVTLKAPGGIAKQSKTSHPIGTTVTILNFMSKLPVRKQTALKSASKTLVKTKELLQAYALARPSIRFSLKITKDSKGSWSFAPRPNDGVKEAVSHIIGKEASMQCLSKTLTFSEPRKTGDNEFHVEIYLPKPDADISKVGHGQYLSIDSRPVSHEKGTMKKIVSLFKSYLKVGLGEAREVRNPFIRININCPLSSYDPNVEPAKDDVIFGNESLILDPFESFFKDLYGEKESASNSTSKNKPAKPIDNFELLLARNPVVGVPSLNKGTARSDSNVQLKIIEFEDDSASAEKGVPDMTQDENSLFVPEAGETSPAIGKSRRSWGIDMSRDFDEVVTDPRSAVLNRKAKSSPAAIEDDSSNTLNPWVIAKMTAPLRTNAEPVAPTDQESRTPGAANDQYHIIDGNLIRRKSQEQLFLESPVQKHGSVSRRTPKRRYSNEARLPNPNTPKASLYELDRGTRHRSEDGNIYLEQRAKAFRDTTLETPDIPSLHRRQEHSSSEFGHELPNFNEQSVRRRQNDFVSARSIKESFLLTPPATQRQKPSHQSDKVNKPFAAPRRIADTTSTPTRLQQTRLPTFQGQQDFLGVEEPQSELEWAMQYEQNKESASRTRRDQLRLARKDTETETRTERTKSSPHKNRYNAAIAALEGDAVSKPQEPKTSLPPNDPRAYLMKHQALSSTADGEQTKAIRNTRAKSTKLPLEKIPDHLQTHKLVLNLSTDMEKIKEITEMFTPIDAYISQGRSEPGLELSVAATKDLSRDIKRMVDAWVEKQEDRRDCEVEYLFANLNSVEA” |
| *Monosiga brevicollis* MX1 XP_001745742.1 (Mlh1, MONBRDRAFT_25323) | | |
|  | old | /coded_by="XM_001745690.1:1..2949" |
|  | new | /coded_by=”complement(join(NW_001865046.1:1143758..1143775, NW_001865046.1:1143866..1143973, NW_001865046.1:1144031..1144058, NW_001865046.1:1144255..1144317, NW_001865046.1:1144434..1144579, NW_001865046.1:1144662..1144730, NW_001865046.1:1144834..1144885, NW_001865046.1:1145084..1145300, NW_001865046.1:1145412..1145557, NW_001865046.1:1145825..1146036, NW_001865046.1:1146145..1146291, NW_001865046.1:1146397..1146642, NW_001865046.1:1146743..1146863, NW_001865046.1:1146975..1147119, NW_001865046.1:1147242..1147295, NW_001865046.1:1147366..1147456, NW_001865046.1:1147611..1147796, NW_001865046.1:1147903..1148075, NW_001865046.1:1148179..1148269, NW_001865046.1:1148356..1148402, NW_001865046.1:1148488..1148559))” |
|  | old | /sequence=”MWRAVARAKLVLVDAGAVGEFVCVCVCVCVCVCVCVCVCVCVCVCVCVCVCCQFLFGQTQRLQAAARAELYLCADVVEACFWFGPALCRASQIIQRPANAIKEMLENSLDAGSTSVTITVKQGGIKFLQIQDNGHGINKEDMDIVCERFTTSKLSAYEDLQTIATYGFRGEALASISHVAHLSIITKTPTSPCAYSASYRDGKMVAERPDKPAAPKPCAGNTGTQITVEELFYNVQARRRALKNYNDELNRIVDVVSRYAIHNSGAGLTLKKFGEATALVRTTASGSTIDNIRAVYGNAIAQELIEIKREDNDLNFKLEGYMTNPNYNTKKQTLILFINHRSVRSSTIKKAIDEVYSAYLPRGMHSFAYLSLLIKPELVDVNVHPTKHEVHFLNEEEIVQSIAVAVSEALLGSNTSRTFQTQTLLPGAASLSSKPESAPVASQRTRRSQRAVAAEEDRLLYDHDIVRTDSASQSLLKAQPAAAARSSQRASLARRSSDASSATATPSATPARTSQRSARAQRRDNEVQDLLDDLQSQSQSQSQSQSQSQSQSQQIPRRSHAEDDPDEVTMEQAADVESDEDNEVNDEENNTNNEVALPVAQESQATDSTDLSFALTATPDTTANAPIVLDEPESTTADAETIGVEHVDAHISQSDICEGDNAVTAMGHAHSDGNLAGSAKRDVATSDAFRDLLRGHVFVGCIDQSLALAQHQHNLYLIKTRLLSEELFAQLCLRGFAALSPIVLDPAPSVSELLRQALDMPESEWTSEDGPKDELAQNMAVFIQEKSAMLTEYFALEIGEQGQLVSVPCLLEGHLPDFSGLPLFLLNLITDVDWKEEKACFSTVAQQIGQLKTLGKMIIGCDKIMLWQWIASLIIIIPLCPNTWVTPGQVEHVLFPAFRNMYHPSKQVAEKGGALRVANLKELYKLKPIGRERIEDRVLGGFGFPTGPTGLTQSTTHSHSIKNKGGMRKGPGAPLEAAYLRS” |
|  | new | /sequence=”MWRAVARAKLVLVDAGAVGEFVCVCVCVCVCVCVCVCVCVCVCVCVCVCVCCQFLFGQTQRLQAAARAELYLCADVVEACFWFGPALCRASQIIQRPANAIKEMLENSLDAGSTSVTITVKQGGIKFLQIQDNGHGINKEDMDIVCERFTTSKLSAYEDLQTIATYGFRGEALASISHVAHLSIITKTPTSPCAYSASYRDGKMVAERPDKPAAPKPCAGNTGTQITVEELFYNVQARRRALKNYNDELNRIVDVVSRYAIHNSGAGLTLKKFGEATALVRTTASGSTIDNIRAVYGNAIAQELIEIKREDNDLNFKLEGYMTNPNYNTKKQTLILFINHRSVRSSTIKKAIDEVYSAYLPRGMHSFAYLSLLIKPELVDVNVHPTKHEVHFLNEEEIVQSIAVAVSEALLGSNTSRTFQTQTLLPGAASLSSKPESAPVASQRTRRSQRAVAAEEDRLLYDHDIVRTDSASQSLLKAQPAAAARSSQRASLARRSSDASSATATPSATPARTSQRSARAQRRDNEVQDLLDDLQSQSQSQSQSQSQSQSQSQQIPRRSHAEDDPDEVTMEQAADVESDEDNEVNDEENNTNNEVALPVAQESQATDSTDLSFALTATPDTTANAPIVLDEPESTTADAETIGVEHVDAHISQSDICEGDNAVTAMGHAHSDGNLAGSAKRDVATSDAFRDLLRGHVFVGCIDQSLALAQHQHNLYLIKTRLLSEELFAQLCLRGFAALSPIVLDPAPSVSELLRQALDMPESEWTSEDGPKDELAQNMAVFIQEKSAMLTEYFALEIGEQGQLVSVPCLLEGHLPDFSGLPLFLLNLITDVDWKEEKACFSTVAQQIGQLKTLGKMIIGCDKIMLWQWIASLIIIIPLCPNTWVTPGQVEHVLFPAFRNMYHPSKQVAEKGGALRVANLKELYKVFERC” |
| *Mycosphaerella populorum* SO2202 (anamorph: *Septoria musiva* SO2202) EMF10838.1 (Mlh2, SEPMUDRAFT_10186) | | |
|  | old | /coded_by=”KB456267.1:1532781..>1533818” |
|  | new | /coded_by=”KB456267.1:1532781..1535594” |
|  | old | /sequence=”MGIEALPQATVRVLGASQVLTDPAAVVKELLDNAYDANATSIAVEIHNNTIDVIQVRDNGHGVAPQDRPLIARRYCTSKISHDSELKDIGGSSLGFRGEALASAAELSGSLTISTRIEGEQVAAALKISQNGEVVGQEKASLPVGTTVRITDFIKSNPVRRQVVLKGTENCLKKIKRTLQAYAFARPHVRLSLRVLKAKNTKSDWVYAPKLNGNAEDAAFKVVGAACASQCMWSVLESGGFSFHAFLPCLDADPSKISNVGPFISVDSRPVSAARGIFKQVSKIFREALKSAAASLVDLKDPFLYLEIQCPRGSYDANLEPAKDDLLFEDADVVIDTARRLFAMAY” |
|  | new | /sequence=”MGIEALPQATVRVLGASQVLTDPAAVVKELLDNAYDANATSIAVEIHNNTIDVIQVRDNGHGVAPQDRPLIARRYCTSKISHDSELKDIGGSSLGFRGEALASAAELSGSLTISTRIEGEQVAAALKISQNGEVVGQEKASLPVGTTVRITDFIKSNPVRRQVVLKGTENCLKKIKRTLQAYAFARPHVRLSLRVLKAKNTKSDWVYAPKLNGNAEDAAFKVVGAACASQCMWSVLESGGFSFHAFLPCLDADPSKISNVGPFISVDSRPVSAARGIFKQVSKIFREALKSAAASLVDLKDPFLYLEIQCPRGSYDANLEPAKDDLLFEDADVVIDTARRLFAMAYERQSTGAQLLRPETADMPRQQLQQDLDKSASNIPDRTSPGQAQPEDLGAFADLESGNGVHDPASSMDCNEELPTSSTYRSNMYGCDEEDLGVLDQRTSTSHSGADFAELRRLKDDVTVNNPWIAAKLNASVRRPMPVAEDQTYLRNRSINAELTTPVTSSSPLKQGTIASAALPTPRPSSPSPAHNDGFNPSDHVPDIRLARDGRVIGSSALARPEPYTPMSSASTYRHPEYNYAPTSDNSRGTPLEAIPDISSKPRRNPRRGPYQGQTNRPFVSPMTDQSSREKVWFDHLQGADRPRAPSTKRRKNAFNDHGGLVVQGELGDLVEDPRPMTPPLRNRDIRDFVGREEDASVGSMIAHDNRTRAESALRVPRSEREVLDLSGDDAADGRPHQTLDFVPASEILGLSERMGSQDQMFKRVLKRRKTSEKRALQEVDLNAVSHEPSKDRTTFDEEEYQPAAGKRTRSRRKSSSKLGRTKSSKLPLERIPPGKGTHDLAVTCSTSSQNISRMTEKPHIANALLGFNEPALPMADGMDSASSHLLSLAANLHGLLVSAGGDVDSPGPAALLSEVKAAFAERREVHEDEMLMPSGT” |
| *Paracoccidioides brasiliensis* Pb01 XP_002794605.1 (Mlh2, PAAG_03150) | | |
|  | old | /coded_by=”join(NW_003217280.1:631581..631857,NW_003217280.1:631910..633852)” |
|  | new | /coded_by=”join(NW_003217280.1:630983..631389,NW_003217280.1:631457..631857,NW_003217280.1:631910..633852)” |
|  | old | /sequence=”MLIAYALSRPEIRLSLQVLGGKNQSPWIYAPKKTISGAVVAAVGPEVISQCIIVAWPESQDGEANSSSGHKDDSETNPLSIRLVAVVPGPDADCSKLTRAGQFVSIDRRPMSTSHGIAKEIVKLFKCYLRSGSLCNMDLTSPVDPFLFIHLQYPLGSYDANVEPSKDDVLFAASKLVMSLAEDLFKSVYGEIISEDTPENIKAPDSRALSCQAGYIFDRPLANNFQTTKAKEMPFFRRHILRNAMENDHSVDRSFANPLQRNTVNTSLGSSTDTVTFSRPNENNSHVAKNSHQANPWMLTRSRCITLPRENLQSYAANSHLLTPICEDDRFPPDTGLINRGRRNSIQGNQSYPNPLSVQVPDTKFIKYDKSLDHVRKSHMSELGNLTQKSPSKGTIQNRELGGLGCLDAWLRNFRDPKIQPTQENQQSGILQDGEVGEDGLIELVIAGRFGEESSDGPRSYNQKIYSQGPPPRASPNASDVDRESTLIEMHIDDIAPVTCSKLDNNQNGRICEPASRHLSSASQEMVADALDFEYRKRAAIQAQRIMERQQVSSQTAFFSSNGVSRTQTSQSSPYQNRYLRANADLAQQEPDLLRHETAFSLCKTEQHPRTYYKRIRNIVPQNAAISTGLKWKRLLTNQFPLDTTPEAYRLDGFIVTWTSADNEMSIPPKALSETDEYIRTGLVPPSSAFLEVQPTEIAQWTARLLTLIQEQYQSENPGEGIAQLVFDEISTSHATALP” |
|  | new | /sequence=”MVISALHQDTARAVRSAQVLSGPYSVVKELVDNGLDARATSISVELSANTVGIIQVRDNGTGIHPNDRQLVCKRSCTSKLRTVDDLRNVGGSTLGFRGEALASVAEMCGGILVTTKTDEELVGTALRYDRAGLLISCTKSSHPTGTTVRVSEFLKFVPVRKQTALKCASKTISRLKKMLIAYALSRPEIRLSLQVLGGKNQSPWIYAPKKTISGAVVAAVGPEVISQCIIVAWPESQDGEANSSSGHKDDSETNPLSIRLVAVVPGPDADCSKLTRAGQFVSIDRRPMSTSHGIAKEIVKLFKCYLRSGSLCNMDLTSPVDPFLFIHLQYPLGSYDANVEPSKDDVLFAASKLVMSLAEDLFKSVYGEIISEDTPENIKAPDSRALSCQAGYIFDRPLANNFQTTKAKEMPFFRRHILRNAMENDHSVDRSFANPLQRNTVNTSLGSSTDTVTFSRPNENNSHVAKNSHQANPWMLTRSRCITLPRENLQSYAANSHLLTPICEDDRFPPDTGLINRGRRNSIQGNQSYPNPLSVQVPDTKFIKYDKSLDHVRKSHMSELGNLTQKSPSKGTIQNRELGGLGCLDAWLRNFRDPKIQPTQENQQSGILQDGEVGEDGLIELVIAGRFGEESSDGPRSYNQKIYSQGPPPRASPNASDVDRESTLIEMHIDDIAPVTCSKLDNNQNGRICEPASRHLSSASQEMVADALDFEYRKRAAIQAQRIMERQQVSSQTAFFSSNGVSRTQTSQSSPYQNRYLRANADLAQQEPDLLRHETAFSLCKTEQHPRTYYKRIRNIVPQNAAISTGLKWKRLLTNQFPLDTTPEAYRLDGFIVTWTSADNEMSIPPKALSETDEYIRTGLVPPSSAFLEVQPTEIAQWTARLLTLIQEQYQSENPGEGIAQLVFDEISTSHATALP” |
| *Paracoccidioides brasiliensis* Pb18 EEH46931.1 (Mlh2, PADG_03029) Stop codon in first exon. | | |
|  | old | /coded_by=”join(DS572752.1:2140642..2140680,DS572752.1:2140807..2141048,DS572752.1:2141119..2141519,DS572752.1:2141572..2142596,DS572752.1:2142627..2142848,DS572752.1:2142922..2143326)” |
|  | new | /coded_by=”join(DS572752.1:2140642..2141048,DS572752.1:2141119..2141519,DS572752.1:2141572..2142596,DS572752.1:2142627..2142848,DS572752.1:2142922..2143350)” |
|  | old | /sequence=”MAISALRQDTARAVRDNGTGIHPNDRQLVCKRSCTSKLRTVDDLRNVGGSTLGFRGEALASAAEMCGGILVTTKTDEELVGAALKYDRAGLLISCTKSSHPTGTTVRVSEFLKFVPVLKQTALKCASKTISRLKKMLIAYALSRPEIRLSLQVLGGKNQSPWIYAPKKTISDAIMTAVGPEVVSQCVIVAWPESQDSEASSSLGHRDDSETNPLSIRLVAVVPGPDADCSKLTRAGQYVSIDRRPMSTSHGIAKEIVKLFKSYLRSGSLCNGGLTSPVDPFLFIHLQCPLGSYDANVEPSKNDVLFADSKLVMSLAEDLFKSVYGEITPKDTRNTVNTSLGSSADTVTFSRPNENNPHVAKNSHQANPWMLTRSRCITPPRENLQSYAAKSYLLTPICEDDRFPPDTGLINRSRRNSIPGNQSFPNPLSVQGPDTKFIKYDKSLDHVRKSHMSELGNLAQKSPTKGAIQNRELGVLGCLDAWLRNFRDPKIQPTQENQQSGILQDGEVGEDGLIELATAGRFGEESSDGPRSYNQKIYSRGSSPRASPNASDVDRESALIDMHIGDLAPNWRICEPTSRHLSSASQEMVADALDFEYRKRAAIQAQRIMQRQQVSSQTAFFSSNEVNRTQTSQSSPYQNRYLRIKTQGHIISALGMPSRKMQPYRQAYKRKRILTNQFPLETTPEAYRLHRVIVNWTSADNETSIPPKALLETDEYIRTGLVPPSSAFFEVQPTEIAQWTARLLTLIQEQYQSENPGEGITQLVFDEISTSHATALP” |
|  | new | /sequence=”MAISALRQDTARAVRLAQVFSDPYSVVKELVDN*LDAGATSISVELSANTVGIIQVRDNGTGIHPNDRQLVCKRSCTSKLRTVDDLRNVGGSTLGFRGEALASAAEMCGGILVTTKTDEELVGAALKYDRAGLLISCTKSSHPTGTTVRVSEFLKFVPVLKQTALKCASKTISRLKKMLIAYALSRPEIRLSLQVLGGKNQSPWIYAPKKTISDAIMTAVGPEVVSQCVIVAWPESQDSEASSSLGHRDDSETNPLSIRLVAVVPGPDADCSKLTRAGQYVSIDRRPMSTSHGIAKEIVKLFKSYLRSGSLCNGGLTSPVDPFLFIHLQCPLGSYDANVEPSKNDVLFADSKLVMSLAEDLFKSVYGEITPKDTRNTVNTSLGSSADTVTFSRPNENNPHVAKNSHQANPWMLTRSRCITPPRENLQSYAAKSYLLTPICEDDRFPPDTGLINRSRRNSIPGNQSFPNPLSVQGPDTKFIKYDKSLDHVRKSHMSELGNLAQKSPTKGAIQNRELGVLGCLDAWLRNFRDPKIQPTQENQQSGILQDGEVGEDGLIELATAGRFGEESSDGPRSYNQKIYSRGSSPRASPNASDVDRESALIDMHIGDLAPNWRICEPTSRHLSSASQEMVADALDFEYRKRAAIQAQRIMQRQQVSSQTAFFSSNEVNRTQTSQSSPYQNRYLRIKTQGHIISALGMPSRKMQPYRQAYKRKRILTNQFPLETTPEAYRLHRVIVNWTSADNETSIPPKALLETDEYIRTGLVPPSSAFFEVQPTEIAQWTARLLTLIQEQYQSENPGEGITQLVFDEISTSHATALP” |
| *Penicillium digitatum* Pd1 EKV21766.1 (Mlh2, PDIP_03130) Frameshift in final exon | | |
|  | old | /coded_by=”complement(join(AKCU01000026.1:8844..10447,AKCU01000026.1:10497..10885,AKCU01000026.1:10944..11350))” |
|  | new | /coded_by=”complement(join(AKCU01000026.1:8576..8914,AKCU01000026.1:8916..10447,AKCU01000026.1:10497..10885,AKCU01000026.1:10944..11350))” |
|  | old | /sequence=”MPIEALPQKTIRAIGSTSVISDPYSVIKELVDNALDAFATSLQIEISQNTVDVIQLKDNGHGISPEDQQHVCKRAFTSKIRTLDDLKNVGGSSLGFRGEALASVAEMSGVLAVTTRVESEVTGFCLKYGRNGELTGTQRKSHPVGTTVRITDFLKHIPVRRQTAVKSATKDLTRIKKLLQAYAIAQPSKRLSFKVLKAKNENSSWAYAPSADASLSDAALKITGTDVYSSCVMKRIACPRTAENYRGSLNQKEYEVIAFLPKTQFDTSKINNAGQYISVDGRPLSSCRGVGHEIVKIFKVYLRVAASKNESTKSISDPFVCLQIRCPRGTYDVNIEPAKDDLLFEDRDVVLALVEKLFRDHYGKIHGTETGSYNQGKEDACKPGGNLGGFKILMARKPATELSPQPRNSEHSFDQTVPHTPLSQKPLLSENAFSPVAPSSYKDPESLSKSTSARNERSSFVNPWSISRINASLRTPRRGSNSSKQASPAELSSSSLQGLNRWETESRGFQHSPMSDLASPITSRIASKSPVRRLRQKPQDLVESSPETNRISSAQCAERGNDRDRHGNGALDTWFQRTTQVSLQQTPAEEGPDSSLSFLAQQRFGVLTNTLPNILCVGGQNYSCSDSSSRNSIHTKAANHRRSLQDEKEENVPDPLKSGQGFPVLDRWAAQLHESVSHEEPSDLEKALDFERRKKEAIQNSRTRFKMNEKPSSSQSALASHSPHRSRYLAAKAALTSSQTSIDEPISATKLSPHDPRAYLIRQARDLSADKSSTGGWKNRENTHQPTSLRTYTRWLRPA” |
|  | new | /sequence=”MPIEALPQKTIRAIGSTSVISDPYSVIKELVDNALDAFATSLQIEISQNTVDVIQLKDNGHGISPEDQQHVCKRAFTSKIRTLDDLKNVGGSSLGFRGEALASVAEMSGVLAVTTRVESEVTGFCLKYGRNGELTGTQRKSHPVGTTVRITDFLKHIPVRRQTAVKSATKDLTRIKKLLQAYAIAQPSKRLSFKVLKAKNENSSWAYAPSADASLSDAALKITGTDVYSSCVMKRIACPRTAENYRGSLNQKEYEVIAFLPKTQFDTSKINNAGQYISVDGRPLSSCRGVGHEIVKIFKVYLRVAASKNESTKSISDPFVCLQIRCPRGTYDVNIEPAKDDLLFEDRDVVLALVEKLFRDHYGKIHGTETGSYNQGKEDACKPGGNLGGFKILMARKPATELSPQPRNSEHSFDQTVPHTPLSQKPLLSENAFSPVAPSSYKDPESLSKSTSARNERSSFVNPWSISRINASLRTPRRGSNSSKQASPAELSSSSLQGLNRWETESRGFQHSPMSDLASPITSRIASKSPVRRLRQKPQDLVESSPETNRISSAQCAERGNDRDRHGNGALDTWFQRTTQVSLQQTPAEEGPDSSLSFLAQQRFGVLTNTLPNILCVGGQNYSCSDSSSRNSIHTKAANHRRSLQDEKEENVPDPLKSGQGFPVLDRWAAQLHESVSHEEPSDLEKALDFERRKKEAIQNSRTRFKMNEKPSSSQSALASHSPHRSRYLAAKAALTSSQTSIDEPISATKLSPHDPRAYLIRQARDLSADKSSTGGGKTERTPTNRLPFERIPDGSGLHDLGLTCSVDLSPGSNSFRCNMPENLYTESDNETTAFSTSDLETCLPFWNERLMLIMKRQYKNKDESKPFSIQIDLATKISQQVQTAQIN” |
| *Phaeosphaeria nodorum* SN15 XP_001794729.1 (Mlh2, SNOG_04311) | | |
|  | old | /coded_by=”join(NW_001884554.1:472237..472608,NW_001884554.1:472698..474570,NW_001184554.1:474625..475472)” |
|  | new | /coded_by=”join(NW_001884554.1:472168..472608,NW_001884554.1:472662..475472)” |
|  | old | /sequence=”MGSGQVLVDPSSLVKELIDNALDARAKSIFVDITANTIDSIQIKDDGHGIPAEDRALVCRRYCTSKIRYFYDLKEVGGKWLGFRGEALSSMAEMSGTLAVTTRVEGEPVAVKLKYGRDGEIERGVTQFFEYIPVRRQTATKNAAKCLAKIRRLVQAYALARPAVRFRLHVLKAKNNKGDFIYAPKTNSNIEDAVLKVISKDCALQCDWTALESDGFEVHAFLPKPTANESKIANYGAFISIDSRPVSNSRGTIKQVVAAVKERLRKSSQSLTAAKDPFFCMNIICPPDSYDPNIEPAKDNVMFEDGKVVLDAVDKLLRSYYPEAMETEVEPPIPGQQHAQSDFEDSWNPMPNPVHLEPTAELVGKSKLDQQSKDPRWRSSMYGIDEDDLEHLQEDQPMVIEEEEGRRAADISNPWTVARMNSIIKPKVSTTNGQLPSPVKSSGDVTMQPSSPSVAKTPLRKAQPEPLTPQTSSRPSPASLLDNELGNSLEPFSRFDPEDETLNVSRDKYTVTGYAGFQSGLPLSEQTGAEQANNFPGAKQPRGGQFPIQVTSAAPRGRQRKPTQNDEVEGPDDTWFGQPMRGSQPSQPTRRQKRRGEQGPLLFASNMTSSPRRPILATAKRISDGQLYSEDNTDIRSFFGQRENGRTDSDGRPTKGPSFTPINAQPTAASPISQNVQDPFRPLTIRERPTSQPIFSRVSPVGPRETRNESRIHAEDATSARRRNNGEQFDVYDEDRFQSPMPRPSSAGNLYSNDKPTHNSRDMTAYFKAYQDRENAPANGSTSSIHRHEPRVAPPHELTSKTRRQRRRTTDGVQRTKSSKLPLERIPHSYHIQDIVLTIHSSVTCIIQSSRKLDMRCNSLDYNYSAEDAFDAFTEPVSERKIVAWVTELDTTLNECFEQVPCADVRSVLHEAIQRGLDARKVNEDMEIVQAPAQTLGVVQDSGDNDRRVAEPNRRVATPPDKPDLAEAVTNESGTTHSTSLKAEDEGMSDFDMSQFVDFDIDAVENDAGPSKNARKEFGEDVEDDMLLDL” |
|  | new | /sequence=”MAENPPPTPVPSIAALPPTTARQMGSGQVLVDPSSLVKELIDNALDARAKSIFVDITANTIDSIQIKDDGHGIPAEDRALVCRRYCTSKIRYFYDLKEVGGKWLGFRGEALSSMAEMSGTLAVTTRVEGEPVAVKLKYGRDGEIERGERDSHPVGTTVKVTQFFEYIPVRRQTATKNAAKCLAKIRRLVQAYALARPAVRFRLHVLKAKNNKGDFIYAPKTNSNIEDAVLKVISKDCALQCDWTALESDGFEVHAFLPKPTANESKIANYGAFISIDSRPVSNSRGTIKQVVAAVKERLRKSSQSLTAAKDPFFCMNIICPPDSYDPNIEPAKDNVMFEDGKVVLDAVDKLLRSYYPEAMETEVEPPIPGQQHAQSDFEDSWNPMPNPVHLEPTAELVGKSKLDQQSKDPRWRSSMYGIDEDDLEHLQEDQPMVIEEEEGRRAADISNPWTVARMNSIIKPKVSTTNGQLPSPVKSSGDVTMQPSSPSVAKTPLRKAQPEPLTPQTSSRPSPASLLDNELGNSLEPFSRFDPEDETLNVSRDKYTVTGYAGFQSGLPLSEQTGAEQANNFPGAKQPRGGQFPIQVTSAAPRGRQRKPTQNDEVEGPDDTWFGQPMRGSQPSQPTRRQKRRGEQGPLLFASNMTSSPRRPILATAKRISDGQLYSEDNTDIRSFFGQRENGRTDSDGRPTKGPSFTPINAQPTAASPISQNVQDPFRPLTIRERPTSQPIFSRVSPVGPRETRNESRIHAEDATSARRRNNGEQFDVYDEDRFQSPMPRPSSAGSERPPLMPIRSNGVRTALDLYSNDKPTHNSRDMTAYFKAYQDRENAPANGSTSSIHRHEPRVAPPHELTSKTRRQRRRTTDGVQRTKSSKLPLERIPHSYHIQDIVLTIHSSVTCIIQSSRKLDMRCNSLDYNYSAEDAFDAFTEPVSERKIVAWVTELDTTLNECFEQVPCADVRSVLHEAIQRGLDARKVNEDMEIVQAPAQTLGVVQDSGDNDRRVAEPNRRVATPPDKPDLAEAVTNESGTTHSTSLKAEDEGMSDFDMSQFVDFDIDAVENDAGPSKNARKEFGEDVEDDMLLDL” |
| *Pyrenophora tritici-repentis* Pt-1C-BFP XP_001938078.1 (Mlh2, PTRG_07746) 2 frameshifts in final exon compared to *Pyrenophora teres* | | |
|  | old | /coded_by=”join(NW_001932951.1:669590..670026,NW_001939251.1:670083..670567,NW_001939251.1:670635..671481,NW_001939251.1:672114..672534)” |
|  | new | /coded_by=”join(NW_001939251.1:669590..670026,NW_001939251.1:670083..670618,NW_001939251.1:670617..672099,NW_00193925.1:672099..672534)” |
|  | old | /sequence=”MTECPETIPLPGIAALPPTTTRQIGSGQVLVDTSPVVQELIDNALDARAKSMFVDITPNTIDPIQVKDDGHGIPAEDRPLVCRRYCTSKIRDFHDLRNVGGKWLGFRGEALSSMAEMSASLDVTTRVEGELVAVKTKYNRNGELASTVHDSHPVGTTVKVTKFFDYIPVRKQTALKNSSRCLAKIRRLMQAYALARPTTRFRLHVLKAKNSNSDFVYAPKANANIEDAALKVVDKECALQCEWTAMEADGFEVHAFLPKPTANGLKISGQGAFVSVDGRPLSNSRGTIKQIVMAFKELLRKSNSSLAEPAKDDVMFENSEVVLGVFDRLLTSYYPEAVDNVSEEEPPMSAQQHLDPSPEALQRSDRTSIAAKEYAIQGPDDGPKSRAQPRWRSTMYGIDEDDMEFLEESQAPAIDQEEGIRAVEVSNPWTIARMNAAIKPKPVADNGQLLSPAKSQNGVAARSSSPNPSVTPNRPSSARPLTPQTSLRSTVARPPLDEELERSFQRLSQGPSEADAFDDGREERLAEQRHNNLMLPSVGTQRSNHAAPADFQRASLMFPQSSPASQDMSLPAPPAPPKTQRKRQAYANKPVIDGDFETFLDGVDEKTVMRWVIQVDACLESMYERVGGADVRGVLHEGVQRGFDSVVGSMGEGSVEMESMGGSVVRDADSPSLYGGEVGVGGGGGMRGDGYGVERLGSGMSGGMRVSVDDQEVGEEYDDGVEDEMLMDF” |
|  | new | /sequence=”MTECPETIPLPGIAALPPTTTRQIGSGQVLVDTSPVVQELIDNALDARAKSMFVDITPNTIDPIQVKDDGHGIPAEDRPLVCRRYCTSKIRDFHDLRNVGGKWLGFRGEALSSMAEMSASLDVTTRVEGELVAVKTKYNRNGELASTVHDSHPVGTTVKVTKFFDYIPVRKQTALKNSSRCLAKIRRLMQAYALARPTTRFRLHVLKAKNSNSDFVYAPKANANIEDAALKVVDKECALQCEWTAMEADGFEVHAFLPKPTANGLKISGQGAFVSVDGRPLSNSRGTIKQIVMAFKELLRKSNSSLAGVKEPFFCMNIICPPDSFTTLNIEPAKDDVMFENSEVVLGVFDRLLTSYYPEAVDNVSEEEPPMSAQQHLDPSPEALQRSDRTSIAAKEYAIQGPDDGPKSRAQPRWRSTMYGIDEDDMEFLEESQAPAIDQEEGIRAVEVSNPWTIARMNAAIKPKPVADNGQLLSPAKSQNGVAARSSSPNPSVTPNRPSSARPLTPQTSLRSTVARPPLDEELERSFQRLSQGPSEADAFDDGREERLAEQRHNNLMLPSVGTQRSNHAAPADFQRASLMFPQSSPASQDMSLPAPPAPPKTQRKRQAYANKPFAPPPMQSSDGWPGQQFPGSQPPKLSGRQKRTNDPGRSSQALYTQHPSRLEQTERMTGPRIYSEDESDIRNFFGQSRQPRGNSYTQNEELPYPDSQAPPNQPRARISLPSNNANRSLPLPSLKTRKPDTRDQTSAKEIEAYFLSHDHPHPSLPVRTQGKQYKYHPTLERTKSSRLPLERIPLASTPTTLPSTCKSTSTPSPAVFSLLDMESVIDGDFETFLDGVDEKTVMRWVIQVDACLESMYERVGGADVRGVLHEGVQRGFDSVVGSMGEGSVEMESMGGSVVRDADSPSLYGGEVGVGGGGGMRGDGYGVERLGSGMSGGMRVSVDDQEVGEEYDDGVEDEMLMDF” |
| *Setosphaeria turcica* Et28A EOA89057.1 (Mlh2, SETTUDRAFT_83450) | | |
|  | old | /coded_by=”complement(join(KB908526.1:<1229643..1230325,KB908526.1:1230371..1230855,KB908526.1:1230912..1231348))” |
|  | new | /coded_by=”complement(join(KB908526.1:<1229643..1230855,KB908526.1:1230912..1231348))” |
|  | old | /sequence=”MAEASVTITVPGIAALPPTTVRQIGSGQVLVDPSSVVKELIDNALDARAKSIFVDITANTIDSIQVKDDGHGIPGEDRPLVCRRYCTSKIRDLQDLRDVGGKWLGFRGEALSSMADMSGAISVTTRVEGEPVAVKVKYGRNGELDSTEHDSHPVGTTVKVTGFFQHIPVRKQAAPKNATKCLAGIRRLMQAYALARPGTRLRLRVLKAKNANSDFVYAPKADANVEDAVLKIMGRDCAHQCDWTVTESNGFEIHACLPKPTATGPKIANQGSFVSIDARPVASSRGTIKQMISAYKERLRKVNPSLAGSYDPNIEPAKDDVMFDDGKAVLGVFERLLQSYYPEIVVNHDVDSQSTLQNEPQPDNIPEQYQEDTSSQSQSLNEETENPASGISQGQPKWRSSMYGIDEDDLEFLQHHRTPVIEEEEEQGRLAIEVSNPWTIARMNAPVKPKQSISHVQLPSPAKSNRDITLRSSSPGRPATPNPSISADPLTPRTCPNSDAARRALDEVLERSIQRLPRASSVASASIDDSTEEQL” |
|  | new | /sequence=”MAEASVTITVPGIAALPPTTVRQIGSGQVLVDPSSVVKELIDNALDARAKSIFVDITANTIDSIQVKDDGHGIPGEDRPLVCRRYCTSKIRDLQDLRDVGGKWLGFRGEALSSMADMSGAISVTTRVEGEPVAVKVKYGRNGELDSTEHDSHPVGTTVKVTGFFQHIPVRKQAAPKNATKCLAGIRRLMQAYALARPGTRLRLRVLKAKNANSDFVYAPKADANVEDAVLKIMGRDCAHQCDWTVTESNGFEIHACLPKPTATGPKIANQGSFVSIDARPVASSRGTIKQMISAYKERLRKVNPSLAGIKDPFVCVNIICPPGSYDPNIEPAKDDVMFDDGKAVLGVFERLLQSYYPEIVVNHDVDSQSTLQNEPQPDNIPEQYQEDTSSQSQSLNEETENPASGISQGQPKWRSSMYGIDEDDLEFLQHHRTPVIEEEEEQGRLAIEVSNPWTIARMNAPVKPKQSISHVQLPSPAKSNRDITLRSSSPGRPATPNPSISADPLTPRTCPNSDAARRALDEVLERSIQRLPRASSVASASIDDSTEEQL” |
| *Trichophyton verrucosum* HKI 0517 XP_003024108.1 (Mlh1, TRV_01743) | | |
|  | old | /coded_by=”join(NW_003315546.1:599..1036,NW_003315546.1:1098..1432,NW_003315546.1:1488..3179,NW_003315546.1:3265..3600)” |
|  | new | /coded_by=”join(NW_003315546.1:599..1036,NW_003315546.1:1098..1432,NW_003315546.1:1488..3264)” |
|  | old | /sequence=”MDGDRMDLDSTSQGPRGLKRPAPGADAGADTANASSSPAGASGASAAAASAASAASARPRKIQVSHVIQSRVERERERADLTDGQKALDPDVINKIAAGEIIVAPMHALKELIENSVDAGSTSVEILVREGGLKLLQITDNGHGIDHDDLSILCERFTTSKLQAFEDLSSIATYGFRGEALASISHVAHLTVTTKTAGSSCAWRAHYSDGKLVPAKPGQNASPKPIAGRKGTQITVSTALENPSIIFSLSIYMHMLTRSASEEYAKILDIVGRYAVHCSGTAFSCKKHGEAGVSLSTSINSSILDRIRQLHGGAVANELVSLEVDGKRWGCRASAWVTNANYHAKKTTLLIFINHRAVESTAIKRAVEQTYSTFLPKGGHPFVYLDLEIEPQRLDVNVHPTKREVNFLNEDEIIESICSAIRTKLAAVDSSRTFMTQTLLPGIRPPEPATLAGDASSGAEGERLALRTVAGTKRPYENNLVRTDAKLRKITSMLPPAGSETVHGDKPSGNQGLAYQKVNREPVNIRLTSVKNLRAAVRSSMHNNLTEIFSSNTYVGLVDERRRVAAIQSGVKLYLVDYGMVCNEFFYQLGLTNFGNFGSINLESSPKLVDLLSLAVEVERDEYYRNNPPDGDAASVASDASRSIDEGIVVDFTSVAATVAKHLIDRREMLKEYFSLSISEDGCLLSIPLLLKGYMPSLVKLPRFLLRLGPYVDWSGEEACFRTFLTELAAFYTPEQLPTPYSSSTPQGGCGRQPGPGARESSPHSIVSDISRENGVSATESPQADQSSSHDEAESEDESVTRRREQLSWMLEHTLFPAIRSRVLIAFKHPPSCLYSPIPSTTSLQKENSLSASYIDDTVTTYTVPFYGLTYLPARSMI” |
|  | new | /sequence=”MDGDRMDLDSTSQGPRGLKRPAPGADAGADTANASSSPAGASGASAAAASAASAASARPRKIQVSHVIQSRVERERERADLTDGQKALDPDVINKIAAGEIIVAPMHALKELIENSVDAGSTSVEILVREGGLKLLQITDNGHGIDHDDLSILCERFTTSKLQAFEDLSSIATYGFRGEALASISHVAHLTVTTKTAGSSCAWRAHYSDGKLVPAKPGQNASPKPIAGRKGTQITVSTALENPSIIFSLSIYMHMLTRSASEEYAKILDIVGRYAVHCSGTAFSCKKHGEAGVSLSTSINSSILDRIRQLHGGAVANELVSLEVDGKRWGCRASAWVTNANYHAKKTTLLIFINHRAVESTAIKRAVEQTYSTFLPKGGHPFVYLDLEIEPQRLDVNVHPTKREVNFLNEDEIIESICSAIRTKLAAVDSSRTFMTQTLLPGIRPPEPATLAGDASSGAEGERLALRTVAGTKRPYENNLVRTDAKLRKITSMLPPAGSETVHGDKPSGNQGLAYQKVNREPVNIRLTSVKNLRAAVRSSMHNNLTEIFSSNTYVGLVDERRRVAAIQSGVKLYLVDYGMVCNEFFYQLGLTNFGNFGSINLESSPKLVDLLSLAVEVERDEYYRNNPPDGDAASVASDASRSIDEGIVVDFTSVAATVAKHLIDRREMLKEYFSLSISEDGCLLSIPLLLKGYMPSLVKLPRFLLRLGPYVDWSGEEACFRTFLTELAAFYTPEQLPTPYSSSTPQGGCGRQPGPGARESSPHSIVSDISRENGVSATESPQADQSSSHDEAESEDESVTRRREQLSWMLEHTLFPAIRSRLVATNDLVRGVIEVADLKGLYRVFERC” |
| *Trichophyton verrucosum* HKI 0517 XP_003019186.1 (TRV_06792, Mlh2) + XP_003019185.1 (TRV_06791) | | |
|  | old | XP_003019186.1 /coded_by=”complement(ACYE010000389.1:38736..39278)”  XP_003019185.1 /coded_by=”complement(ACYE01000389.1:38129..38357)” |
|  | new | /coded_by=”complement(join(ACYE01000389.1:36709..38081,ACYE01000389.1:38129..38526,ACYE01000389.1:38740..39146))” |
|  | old | XP_003019186.1 /sequence=”MATPDLNSSTSSISPSCDDILISLRSQLNLSPFSIDYDIQFKGNMSIVALEDPAVRAIGSTSALPDSSSVVKELLDNALDAGATSIFIEISLNTLDIIQVKDNGSGILPSDRSLACKQNYTSKIQTTEDLKNVGGRSLGFRGQALASIAEMSDAMYITTRVPEEQVARTVKFGRDGEPIR”  XP_003019185.1 /sequence=”MRVLKSKEKDWVYAPSQKPSVPDAILQVIGSTVISSCISSNASYPIPGEKDSPDSMVENYDTPTIHMSIIVPNPEAECMARINHKGQFVIVDARPLLTCMGFGKEVFKLFKSYYKKAVNSNILEDPFMFLSLDCPPGIYDANIEPSKDDILFEDHQAVLQIIEGVFMDIYNLETSTIIPTPDTQQSYLSNQLTIDISSPASTKNRGRFRSNEKLTSTVNPWTLSLAAQRLRDPESQLLTPQREFQDPRNNIPPRSPNIRVKNSRISMRQATLSLHDNGRVSLFNTQSPQRTSNGSPRMVTPPRVPAPNIPRSIEASVQIAEGNSARGKEGMYVIRPTPQTPPPSRAFSAATHRQAPIAVTFSRSNDQIPPNPHSISGRLHCNHRSDHKISGYGEIRPYKLTVPSGSHLQTASTNLKSRFQPDLNQKRNHLTSYRKNNFSTEIILYQSQSTMECMYETTIPNGGETTQLADSIKRLVNTDQYVKSGSLMAAFSPADLTSSISYWTNKLHTLAKNPSFQRQLRQLSFLTPSNSSK” |
|  | new | /sequence=”MSIVALEDPAVRAIGSTSALPDSSSVVKELLDNALDAGATSIFIEISLNTLDIIQVKDNGSGILPSDRSLACKQNYTSKIQTTEDLKNVGGRSLGFRGQALASIAEMSDAMYITTRVPEEQVARTVKFGRDGEPISDTPASHPIGTTVRVCDFLKSLPVRRQEAEKKSTKSILAIKKLLRGYAIARPKTRLSMRVLKSKEKDWVYAPSQKPSVPDAILQVIGSTVISSCISSNASYPIPGEKDSPDSMVENYDTPTIHMSIIVPNPEAECMARINHKGQFVIVDARPLLTCMGFGKEVFKLFKSYYKKAVNSNILEDPFMFLSLDCPPGIYDANIEPSKDDILFEDHQAVLQIIEGVFMDIYNLETSTIIPTPDTQQSYLSNQLTIDISSPASTKNRGRFRSNEKLTSTVNPWTLSLAAQRLRDPESQLLTPQREFQDPRNNIPPRSPNIRVKNSRISMRQATLSLHDNGRVSLFNTQSPQRTSNGSPRMVTPPRVPAPNIPRSIEASVQIAEGNSARGKEGMYVIRPTPQTPPPSRAFSAATHRQAPIAVTFSRSNDQIPPNPHSISGRLHCNHRSDHKISGYGEIRPYKLTVPSGSHLQTASTNLKSRFQPDLNQKRNHLTSYRKNNFSTEIILYQSQSTMECMYETTIPNGGETTQLADSIKRLVNTDQYVKSGSLMAAFSPADLTSSISYWTNKLHTLAKNPSFQRQLRQLSFLTPSNSSK” |
| *Uncinocarpus reesi* 1704 XP_002543201.1 (Mlh2, UREG_02717) | | |
|  | old | /coded_by=”join(NW_003052500.1:7240488..7240807,NW_003052500.1:7241028..7241338,NW_003052500.1:7241478..7242892)” |
|  | new | /coded_by=”join(NW_003052500.1:7240488..7240898,NW_003052500.1:7240957..7241338,NW_003052500.1:7241391..7242892)” |
|  | old | /sequence=”MPIAALDEAAAKAIGSASALYDPCCVVKELVDNALDANATIVSIELSSNTLDTIQVKDNGLGIPLEDRDLVCKRNHTSKIRSLEDLKNVGGSSLGFRGQALASTAEMQVMLKSSTQNISRIRRTLQEYALSRVHVRFSLKILKHQQDSAWVYAPKQNPSVTDAISKIIGVDVMAQCLSQTTDLVNNSIPNRENESHSGIRLHAILPKLDAVKIYKSYIKYTFERLPSSTVANPFLCLYIFCAEGSYDVNIEPSKDDVLFENQALVSEAVEGLFEGIYGKLQSRGENQVHSQMGASHNLHNPDQNLFLGLTPSKPLNKEQTRLATPFGPTAQQLECNSTSRTNRSTGRIPNTNPWSLAKRSSAQRCRASNSSLLTPSPDSSSPIVNNVNPQTNYSSINGYLYLGPSPSSRPDSKNSPVKRTLPAIETQHAKRQGILSQGSKNDGGPRPERFVEGMSTGPLDRIPKRTAGGDSVQQPGDADLGIPLAARFGDPINTPRISCPTPKPSIETGKGDPRSLGPSRASSNPLHALHPPPFSLEKSIDFERKHATINLAARATNNNHLDLSLISKGKRSGAEEALLVENDGPCDDVNRMAIRYPSPMPDAGAVHHLAVSCNTRITDIEPIMRQLWGVDSFIQTGKSTPKLVLSCPAKVIEHWKATTLNLVRKSWHTETAKPCITLNLP” |
|  | new | /sequence=”MPIAALDEAAAKAIGSASALYDPCCVVKELVDNALDANATIVSIELSSNTLDTIQVKDNGLGIPLEDRDLVCKRNHTSKIRSLEDLKNVGGSSLGFRGQALASTAEMCDCLLITTRSLGEIAASALTFSRTGDLLRQAIVSHPLGTSVRVSNFLNNLPVRRQVMLKSSTQNISRIRRTLQEYALSRVHVRFSLKILKHQQDSAWVYAPKQNPSVTDAISKIIGVDVMAQCLSQTTDLVNNSIPNRENESHSGIRLHAILPKLDADISKIGNKGQFIFVDGRPLSPAKGLAKDIVKIYKSYIKYTFERLPSSTVANPFLCLYIFCAEGSYDVNIEPSKDDVLFENQALVSEAVEGLFEGIYGKLQSRGENQVHSQMGASHNLHNPDQNLFLGLTPSKPLNKEQTRLATPFGPTAQQLECNSTSRTNRSTGRIPNTNPWSLAKRSSAQRCRASNSSLLTPSPDSSSPIVNNVNPQTNYSSINGYLYLGPSPSSRPDSKNSPVKRTLPAIETQHAKRQGILSQGSKNDGGPRPERFVEGMSTGPLDRIPKRTAGGDSVQQPGDADLGIPLAARFGDPINTPRISCPTPKPSIETGKGDPRSLGPSRASSNPLHALHPPPFSLEKSIDFERKHATINLAARATNNNHLDLSLISKGKRSGAEEALLVENDGPCDDVNRMAIRYPSPMPDAGAVHHLAVSCNTRITDIEPIMRQLWGVDSFIQTGKSTPKLVLSCPAKVIEHWKATTLNLVRKSWHTETAKPCITLNLP” |
| *Wickerhamomyces ciferrii* CCH43072.1 (Pms1, BN7_2619) | | |
|  | old | /coded_by=”complement(CAIF01000068.1:5725..8502)” |
|  | new | /coded_by=”complement(join(CAIF01000068.1:5627..5710,CAIF01000068.1:5773..8502))” |
|  | old | /sequence=”MSKITSIGKEDVHRITSGQVIIDLSTAVKELLENSIDANSKKIEIIFKNYGLDSIEIIDDGDGIDELDFLNIALKHTTSKLSNFEDLDNVDTLGFRGEAINSLCSISNLKIITTKTPPKAHAIEYLPSGEIHKKTICSRSKGTSIIITNLFNNLPVRRKDFQKNFKKEFSKCLQNLYSYALISLNLKIIVANITINGKKNIVLQTQGNSILKNNIINVFGSNGMYGLIPLDFNLDLNSNKSKLKILNHSIDYNIKINGFISKCSFGFGRSSIDRQYFFINNRPVSLTHFGKAINEVYKSFNHLQYPVFILNFEINPQFLDLNVTPDKKIILIHNEDIILEKLKQELINFYNLQDLSLVRNSTNQQSLDLRSSSMIDDEEDEDVKPIQSSLMTSSFSYNGEEVGNDNDVNESKRKMVNEISPLSKRRKIEEEEEEEGDEEEEVEEVEEENNEFEEEEFDDIISENDQDNNDENKNHSINNTSSKPKTRPSIRQNLPNLKEKFDLNNLSSFRNNQTSSTSQPQPSNITKKSKQSTLEPIFMKIGDKEIIENGYIDKKGTLSFEKCHCGSGHSKSEHPDEEQEGEDHEEEDGVEHIDQDHEENESEILEIENSSKNKLFVPEDYDEIKEPNSISQELIDGNTGLDSNHSIDVDQDLIISDPSTIKSEFNNRFLNSNDRQKNASRNYELLVDSIVNPPKIINEDEEQNRLNTLKNLSIDNIDDQEESENKLTLTVSKKDFLEMKIIGQFNLGFILVIRQNDLKQDLFIVDQHASDEKFNFETLQKITIFDSQPLVVPKKIELNALDELTIIENQQVFVKNGFKFEIDEDGEPGSRIKLISLPLSKKTVFDENDFNELIHLIKENQGNTDSIRCSKIRSMFAMRACRKSIMVGKSLNTKTMTKVIRNLGQLDKPWVSNQQKRKSFESFKY” |
|  | new | /sequence=”MSKITSIGKEDVHRITSGQVIIDLSTAVKELLENSIDANSKKIEIIFKNYGLDSIEIIDDGDGIDELDFLNIALKHTTSKLSNFEDLDNVDTLGFRGEAINSLCSISNLKIITTKTPPKAHAIEYLPSGEIHKKTICSRSKGTSIIITNLFNNLPVRRKDFQKNFKKEFSKCLQNLYSYALISLNLKIIVANITINGKKNIVLQTQGNSILKNNIINVFGSNGMYGLIPLDFNLDLNSNKSKLKILNHSIDYNIKINGFISKCSFGFGRSSIDRQYFFINNRPVSLTHFGKAINEVYKSFNHLQYPVFILNFEINPQFLDLNVTPDKKIILIHNEDIILEKLKQELINFYNLQDLSLVRNSTNQQSLDLRSSSMIDDEEDEDVKPIQSSLMTSSFSYNGEEVGNDNDVNESKRKMVNEISPLSKRRKIEEEEEEEGDEEEEVEEVEEENNEFEEEEFDDIISENDQDNNDENKNHSINNTSSKPKTRPSIRQNLPNLKEKFDLNNLSSFRNNQTSSTSQPQPSNITKKSKQSTLEPIFMKIGDKEIIENGYIDKKGTLSFEKCHCGSGHSKSEHPDEEQEGEDHEEEDGVEHIDQDHEENESEILEIENSSKNKLFVPEDYDEIKEPNSISQELIDGNTGLDSNHSIDVDQDLIISDPSTIKSEFNNRFLNSNDRQKNASRNYELLVDSIVNPPKIINEDEEQNRLNTLKNLSIDNIDDQEESENKLTLTVSKKDFLEMKIIGQFNLGFILVIRQNDLKQDLFIVDQHASDEKFNFETLQKITIFDSQPLVVPKKIELNALDELTIIENQQVFVKNGFKFEIDEDGEPGSRIKLISLPLSKKTVFDENDFNELIHLIKENQGNTDSIRCSKIRSMFAMRACRKSIMVGKSLNTKTMTKVIRNLGQLDKPWNCPHGRPTMRHLMELEQWKPFDDDYRL” |
