# Supplementary material for: Mlh2 Is an Accessory Factor for DNA Mismatch Repair in Saccharomyces cerevisiae
Source: PLoS Genet. 2014 May 8;10(5):e1004327. doi: 10.1371/journal.pgen.1004327 (PMC4014439; doi:10.1371/journal.pgen.1004327)
Supplement: Table S3 — S. cerevisiae strains used in this study. (DOCX) [file pgen.1004327.s007.docx]

| Table S3. Yeast Strains | | |
| --- | --- | --- |
| **Name** | **Relevant genotype*** | **Reference** |
| RDKY1293 | *MATα ura3-52 leu2Δ1 trp1 his3Δ200 pep4::HIS3 prb1Δ1.6R can1* | [68] |
| RDKY2418 | *MATα ura3-52 leu2Δ1 trp1 his3Δ200 pep4::HIS3 prb1Δ1.6R can1 msh2::hisG msh6::hisG* | [72] |
| RDKY5964 | *MATa ura3-52 leu2Δ1 trp1Δ63 his3Δ200 hom3-10 lys2::InsE-A10* | [33] |
| RDKY6051 | RDKY5964 *msh3::HIS3* | [33] |
| RDKY6098 | RDKY5964 *msh3::HIS3 msh6::hphNT1* | this study |
| RDKY7588 | RDKY5964 *PMS1-4GFP.KanMX6* | [33] |
| RDKY7884 | RDKY5964 *exo1::hphNT1* | this study |
| RDKY7893 | RDKY5964 *MLH2-4GFP.KanMX6* | this study |
| RDKY7894 | RDKY5964 *MLH3-4GFP.KanMX6* | this study |
| RDKY7895 | RDKY5964 *KanMX4.pGPD-MLH2* | this study |
| RDKY7896 | RDKY5964 *natNT2.pGPD-MLH3* | this study |
| RDKY7897 | RDKY5964 *natNT2.pGPD-PMS1* | this study |
| RDKY7898 | RDKY5964 *MLH2-9MYC.hphNT1* | this study |
| RDKY7899 | RDKY5964 *MLH3-9MYC.hphNT1* | this study |
| RDKY7900 | RDKY5964 *PMS1-9MYC.hphNT1* | this study |
| RDKY7901 | RDKY5964 *KanMX4.pGPD-MLH2-9MYC.HIS3* | this study |
| RDKY7902 | RDKY5964 *natNT2.pGPD-MLH3-9MYC.HIS3* | this study |
| RDKY7903 | RDKY5964 *natNT2.pGPD-PMS1-9MYC.HIS3* | this study |
| RDKY7904 | RDKY5964 *KanMX4.pGPD-MLH2 natNT2.pGPD-PMS1* | this study |
| RDKY7905 | RDKY5964 *MLH2-4GFP.KanMX6 Nic96-mCherry.hphNT1* | this study |
| RDKY7909 | RDKY5964 *MLH2-4GFP.KanMX6 msh2::HIS3* | this study |
| RDKY7910 | RDKY5964 *PMS1-4GFP.KanMX6 MLH2-tdTomato.hphNT1* | this study |
| RDKY7911 | RDKY5964 *MLH2-4GFP.KanMX6 pol3-01* | this study |
| RDKY7912 | RDKY5964 *MLH2-4GFP.KanMX6 pol3-L612M.natNT2* | this study |
| RDKY7913 | RDKY5964 *MLH2-4GFP.KanMX6 pol2-M644G.natNT2* | this study |
| RDKY7914 | RDKY5964 *MLH2-4GFP.KanMX6 exo1::hphNT1* | this study |
| RDKY7915 | RDKY5964 *MLH2-4GFP.KanMX6 msh3::HIS3* | this study |
| RDKY7916 | RDKY5964 *MLH2-4GFP.KanMX6 msh6::hphNT1* | this study |
| RDKY7917 | RDKY5964 *MLH2-4GFP.KanMX6 msh3::HIS3 msh6::hphNT1* | this study |
| RDKY7918 | RDKY5964 *MLH2-4GFP.KanMX6 pms1::natNT2* | this study |
| RDKY7919 | RDKY5964 *MLH2-4GFP.KanMX6 pms1-E707K* | this study |
| RDKY7921 | RDKY5964 *MLH2-4GFP.KanMX6 msh3::HIS3 msh6-F337A* | this study |
| RDKY7923 | RDKY5964 *mlh2::KanMX4 msh3::HIS3* | this study |
| RDKY7924 | RDKY5964 *mlh2::KanMX4 msh6::hphNT1* | this study |
| RDKY7925 | RDKY5964 *mlh2::KanMX4 exo1::hphNT1* | this study |
| RDKY7926 | RDKY5964 *mlh2::KanMX4* | this study |
| RDKY7965 | RDKY5964 *msh6::hphNT1* | this study |
| RDKY8158 | *MATa ura3-52 leu2Δ1 his3Δ200 hom3-10 lys2::InsE-A10 CMVp(tetR’-SSN6)::LEU2 trp1::tTA* | this study |
| RDKY8159 | RDKY8158 *mlh2::hphNT1* | this study |
| RDKY8160 | RDKY8158 *KanMX4.tetO_2_-Pms1* | this study |
| RDKY8161 | RDKY8158 *KanMX4.tetO_2_-Pms1 mlh2::hphNT1* | this study |
| RDKY8162 | RDKY8158 *Pms1-9MYC.HIS3* | this study |
| RDKY8163 | RDKY8158 *KanMX4.tetO_2_-9MYC.HIS3 mlh2::hphNT1* | this study |
| W9561-17A | *MAT*a *ADE2 lys2::GAL-I-SceI ura3-1::3xURA3-tetOx112 I-SceI_cs_ (ura3-1) TetR mRFP1(iYGL119W) trp1-1 leu2-3,112 his3-11,15 RAD5 can1-100* | [63] |
| RDKY7906 | W9561-17A *MRE11-GFP::hphNT1* | this study |
| RDKY7907 | W9561-17A *PMS1-4GFP.KanMX6* | this study |
| RDKY7908 | W9561-17A *MLH2-4GFP.KanMX6* | this study |
| * All strains are isogenic to S288C, with exception of W9561-17A and indicated derived strains, which are W303 background. The genotype corresponds to the listed strain with the indicated modifications. | | |
|  |  |  |
|  | | |
